# Preferences of patients with chronic low back pain about nonsurgical treatments: Results of a discrete choice experiment

**DOI:** 10.1111/hex.13685

**Published:** 2022-12-08

**Authors:** Gabin F. Morillon, Maria Benkhalti, Pierre Dagenais, Thomas G. Poder

**Affiliations:** ^1^ Montpellier Recherche en Economie University of Montpellier Montpellier France; ^2^ CIUSSS de l'Estrie—CHUS Sherbrooke Quebec Canada; ^3^ Department of Medicine, Faculty of Medicine and Health Science University of Sherbrooke Sherbrooke Quebec Canada; ^4^ Department of Management, Evaluation and Health Policy, School of Public Health University of Montreal Montreal Quebec Canada; ^5^ Centre de recherche de l'Institut Universitaire en Santé Mentale de Montréal CIUSSS de l'Est de l'île de Montréal Montreal Quebec Canada

**Keywords:** chronic low back pain, discrete choice experiment, preferences

## Abstract

**Introduction:**

This study aimed to assess patients' preferences of nonsurgical treatments for chronic low back pain (CLBP).

**Method:**

We conducted a discrete choice experiment (DCE) in Quebec, Canada, in 2018. Seven attributes were included: treatment modality, pain reduction, the onset of treatment efficacy, duration effectiveness, difficulties with daily activities, sleep problems, and knowledge of the patient's body and pain location. Treatment modalities were corticosteroid injections, supervised body‐mind physical activities, supervised sports physical activities, physical manipulations, self‐management courses, and psychotherapy. Utility levels were estimated using a logit model, a latent class model and a Bayesian hierarchical model.

**Results:**

Analyses were conducted on 424 individuals. According to the Bayesian hierarchical model, the conditional relative importance weights of attributes were as follows: (1) treatment modality (34.79%), (2) pain reduction (18.73%), (3) difficulties with daily activities (11.71%), (4) duration effectiveness (10.06%), (5) sleep problems (10.05%), (6) onset of treatment efficacy (8.60%) and (7) knowledge of the patient's body and pain location (6.06%). According to the latent class model that found six classes of respondents with different behaviours (using Akaike and Bayesian criteria), the treatment modality was the most important attribute for all classes, except for class 4 for which pain reduction was the most important. In addition, classes 2 and 5 refused corticosteroid injections, while psychotherapy was preferred only in class 3.

**Conclusion:**

Given the preference heterogeneity found in the analysis, it is important that patient preferences are discussed and considered by the physicians. This will help to improve the patient care pathway in a context of a patient‐centred model for a disease with growing prevalence.

**Patient or Public Contribution:**

A small group of patients was involved in the conception, design and interpretation of data. Participants in the DCE were all CLBP patients.

## 1 INTRODUCTION

Low back pain (LBP) refers to pain occurring in the lumbar region (vertebrae L1–L5) and affects individuals of all ages, genders and conditions.^1^ LBP is classified according to its duration^2^: acute or short‐term back pain (lasting less than 6 weeks), subchronic back pain (lasting between 6 and 12 weeks) and chronic back pain (lasting 12 weeks or longer). As reported by Nieminen et al.,^3^ risk factors of chronic low back pain (CLBP) are physical (e.g., overweighting, hypertension, smoking, congenital problems), social (e.g., work environment) and psychological (e.g., depression, anxiety, behaviour). Besides its cost to society (e.g., USD 100 billion in the United States^4^), LPB impairs many of the various aspects of individuals' daily lives. Its lifetime prevalence is estimated between 60% and 70% in industrialized countries with a peak between the ages of 35 and 55, ^5^ and according to the literature review conducted by Meucci et al.,^6^ the average prevalence of CLBP is 11.84% (from 1% in China to 25.4% in Brazil). Moreover, four‐fifths of individuals will experience back pain in their lifetime, and one‐fifth experiencing acute back pain will develop CLBP.^7^

Individuals with CLBP face several treatment alternatives. As recommended by the guideline of the Institute of Health Economics (Alberta, Canada),^8^ treatments facing CLBP include exercise (e.g., physical, therapeutic, aquatic exercises), yoga therapy, active rehabilitation and self‐management programmes, massage therapy, acupuncture, medication (i.e., nonsteroidal anti‐inflammatory drugs, muscle relaxants, antidepressants and opioids), herbal medicine, behavioural therapy, injection therapy and surgery. Note that nonpharmacologic treatments should be used as first‐line treatments.^9^ However, the lack of certainty about recovery and adverse events is a major issue in the choice of treatment.^10^

Patients' decisions and beliefs are taking a growing place in their care pathways.^11^, ^12^ Patients' preferences are driven by a trade‐off between the perceived risks and the perceived benefits, and considering their preferences, especially when these are strong and linked to social, environmental or psychological factors, and when the outcomes are uncertain, is beneficial to promote shared decisions. In a qualitative survey, Dima et al.^11^ underlined four treatment beliefs expressed by patients in their research of care. The treatment must be credible (i.e., making sense, being in adequation with pain and delivering by the right practitioner), effective, safe and affordable and adequate to the individuals. Better comprehension and shared decisions between the practitioner and the patient often led to greater satisfaction and better results. From their side, Poder and Beffarat^13^ conducted a mixed studies review on LBP to highlight treatments and attributes (i.e., treatments' characteristics), and out of the 13 articles included, they found that patients granted priority to effectiveness, followed by the capacity to realize daily life activities, fit to patient's life and the credibility of the treatment, among others. These studies indicate that knowing patients' preferences could have the potential to improve treatments' adherence and effectiveness.

However, patients' preferences on CLBP treatments are not well known. The present study was the result of a request from an academic healthcare institution in Quebec (i.e., the CIUSSS de l'Estrie—CHUS) to improve the organization of care and provision of CLBP treatments, especially nonsurgical treatments. Consequently, we conducted a discrete choice experiment (DCE) to assess patients' preferences for nonsurgical treatments for CLBP. DCE is a stated preference method that aims to elicit preferences throughout a set of choice tasks on the basis of a finite set of alternatives. This method relies on the random utility theory and on the ability of individuals to make choices between treatments with the same characteristics (attributes) but different modalities (levels).

To our knowledge, only 7 DCEs on patients' preferences specifically refer to back pain treatments with mean samples of about 337 individuals using between 5 and 8 attributes. Different treatments were studied such as pharmaceutical treatments^14^ or both pharmaceutical and surgical treatments.^15^ Moreover, acupuncture and low infrared treatments were studied by Chen et al.,^16^ physical exercises by Aboagye et al.^17^ and Ferreira et al.,^18^ and other pain management programmes by Yi et al.^19^ Finally, Walsh et al.^20^ made focus on treatments' adverse events. The most recurrent attributes were cost, time to effect (i.e., the onset of treatment effectiveness), type of treatment (i.e., modality, type of training, the content of the programme), frequency (i.e., the number of sessions), design (i.e., individually or in the group, the size of the group) and travel time (i.e., from the clinic or the gym). A total of 21 different attributes were used in the studies we found, and all the authors were assessing pain and exercise activities experienced by patients using a set of questions or specific questionnaires such as the Chronic Pain Grade,^21^ the Self‐Efficacy to Exercise Scale^22^ or the Form‐C of the Multidimensional Health Locus of Control,^23^ which examines perceived health status as being dependent from internality, chance and powerful others items.

The main difference in our study is that we only considered first‐line treatments (i.e., nonsurgical treatments) and put emphasis on corticosteroid injections (which was the treatment focus in the evaluation request made by our institution) versus five other first‐line treatments that have shown effectiveness in the scientific literature.

## 2 METHODOLOGY

### 2.1 Survey design

An online questionnaire was conducted in Quebec, Canada, at the end of 2018 among members of a provincial support group for patients with chronic pain, the Association Québécoise de la Douleur Chronique (AQDC). Respondents had to suffer from LBP for at least 3 months to be included in the survey (i.e., to have CLBP). The study was conducted in both French and English, using the online survey platform provided by Sawtooth Software (Sawtooth Software Inc.).

Among the members of the AQDC, it was estimated that about 1,500 suffered from CLBP and it was expected to recruit 15%–20% of them through an invitation letter sent by the president of the AQDC. A minimal sample of 200–300 respondents was thus expected, which is in line with the usual recommendation for a DCE^24^, ^25^, ^26^, ^27^, ^28^ and with the usual empirical findings.^29^, ^30^, ^31^ The invitation letter was sent through an email to all members of the AQDC, specifying that only members with CLBP were invited to participate.

The survey included a first part on health status and sociodemographic data, including specific questions about LBP such as the medical diagnosis, the duration of pain, the frequency of painkillers medication use, other treatments (apart from painkillers) and numeric pain rating scales (NPRS)^32^ (i.e., current pain, worst and average pain in last 2 weeks) ranging from 0 (no pain) to 10 (worst pain). Three scales ranging from 0 to 10 measured personal health and life satisfaction and willingness to take risks. The second part was the DCE itself followed by choice certainty scales from 0 (not certain at all) to 10 (absolutely certain). The third part included follow‐up questions (i.e., choice tasks difficulty, number of dimensions taken into account, self‐ranking of dimensions, responses quality, irritation/boredom) and four health‐related quality of life questionnaires (i.e., EQ‐5D‐5L^33^ with its Visual Analogue Scale [VAS], SF‐6Dv2,^34^ Oswestry Disability Index [ODI]^35^ and Rolland‐Morris Disability Questionnaire [RMDQ]^36^). A Quality‐Adjusted Life‐Year (QALY) derived from the EQ‐5D‐5L and the SF‐6Dv2 equal to 1 indicates a perfect health state while a QALY equal to 0 indicates a state of death. Cronbach's *α* were equal to .783, .801, .842 and .853, respectively. Finally, to compare the relative importance of attributes elicited from the choice exercise with respondents' personal perception and to assess the validity of their choices, we asked them to rank the seven attributes and then the six treatments proposed in the DCE, as well as to complete the four‐item questionnaire from Dima et al.^37^ adjusted for the treatments presented in the DCE. The latter is a validated psychometric questionnaire used to assess patients' beliefs regarding their LBP treatments and to examine the determinants of treatment uptake. Cronbach's *α* ranged from .651 to .818.

### 2.2 DCE design, attributes and levels

The DCE was constructed following the ISPOR recommendations.^24^ We used a mixed‐methods design including a systematic literature review, a patients' focus group (*n* = 4) and discussions with two patients and experts (i.e., two economists specialized in preference‐based studies, one rheumatologist, one ethicist and one public health professional with expertise in equity) as well as with a health technology assessment (HTA) consultative committee.^38^ After discussing a list of 40 attributes with the patients' group, 7 attributes with 3–6 levels were retained and then agreed by the HTA committee: treatment modality, pain reduction, the onset of treatment efficacy, duration effectiveness, difficulties with daily activities, sleep problems, knowledge of his/her body and pain location (Table 1). Based on this, the survey was developed by the research team. The survey was pretested for appropriateness and univocity by all patients from the focus group, as well as by one medical doctor, two patient representatives and one public health expert. This led to minimal changes in the final survey.^38^ The online field survey was also pretested before the launch. This was carried out with the same patients from the focus group and the two patient representatives, as well as with four additional patients who provided their insights about the field survey, including an understanding of the DCE tasks, ease of completion and conviviality of the format used. All levels in the DCE were dummy‐coded. The treatment modality had six levels: corticosteroid injections, supervised body‐mind physical activities, supervised sports physical activities, physical manipulations, self‐management courses, and psychotherapy. Examples of such activities were provided to respondents (Table 1) and were presented as such to participants before the start of the DCE. When selected, the two treatment modalities presented in the DCE worked as a label since they were presented first. For more details, see Poder et al.^38^ and Poder and Beffarat.^13^

**Table 1:** DCE attributes and levels

Attributes	Levels
Treatment modality	Corticosteroid injections
	Supervised body‐mind physical activities (e.g., Yoga, Tai‐Chi, Pilates)
	Supervised sports and physical activities (e.g., active walking, swimming, bike riding, weight training, CrossFit)
	Physical manipulations (e.g., chiropractic, physiotherapy, occupational therapy, kinesiotherapy)
	Self‐management courses (e.g., medication, self‐hypnosis, breathing techniques, relaxation)
	Psychotherapy (e.g., cognitive‐behavioural therapy, posttraumatic shock, progressive muscular relaxation, motivational approach)
Pain reduction	None to very slight
	Slight
	Reduced by half
	No pain
Onset of treatment efficacy	1 month
	6 months
	12 months
Duration effectiveness	Effective for 2 months
	Effective for 6 months
	Effective for 12 months
Difficulties with daily activities	As many difficulties as before
	Fewer difficulties as before
	No difficulties
Sleep problems	As many problems as before
	Fewer problems than before
	No problems
Knowledge of his/her body and pain location	Same knowledge as before
	Knowledge somewhat better than before
	Knowledge much better than before

Abbreviation: DCE, discrete choice experiment.

About 5832 different scenarios were possible resulting from all combinations of attributes and levels, yielding a possibility of 34,006,392 pairs. An orthogonal selection procedure allowed us to generate 600 scenarios combined into 300 pairs. These pairs were divided into 30 blocks. Each respondent had to answer to one block of 10 pairs plus one pair for the rationality test (i.e., one scenario dominating the other one and was presented first in the DCE) and one pair for the temporal consistency test (i.e., choice cards 2 and 12 were identical). The blocks' distribution was run randomly among participants. An illustration of a choice card is given in Supporting Information: Appendix 1.

### 2.3 Models

The Conditional Logit (CL) model^39^ is easy to perform but suffers from two limitations: (1) The independence of irrelevant alternatives (IIA) hypotheses that states that the relative probability of selecting alternatives should not change if we introduce or eliminate another alternative^40^ and (2) it does not consider preference heterogeneity (i.e., that individuals have different preferences). Thus, we used two other models for the analysis of the DCE: a Hierarchical Bayesian (HB) model and a Latent Class Logit (LC) model. The results of the CL model are however presented in Supporting Information: Appendix 2.

A dual‐response none opt‐out option (named ‘none’) was allowed in all models (i.e., respondents could choose neither the first nor the second scenario in each pair). The LC model considers interclass homogeneity and intraclass heterogeneity while the HB model considers that all individuals have different preferences. In the HB model, all parameters were specified as normally distributed. We also performed cross‐tabulation between latent classes clusters (Supporting Information: Appendix 3.1) and segmentation via a hierarchical clustering method using the Ward's minimum variance method and L2 squared dissimilarity measure (Supporting Information: Appendix 3.2). It allowed us to provide a comparison with the LC model. For the latter, we used Akaike and Bayesian information criteria, the log‐likelihood function and the interpretation of the results to determine the number of latent classes (Supporting Information: Appendix 4). Subject preferences were expressed using part‐worth utilities calculated using algorithms in Sawtooth Software Lighthouse Studio version 9.7.2. Utility values were zero‐centred, and the value of the reference modality was calculated as the negative of the sum of the other modalities' values. A high positive level of utility thus indicates a high preference for the level concerned. For comparison purpose, attributes' conditional relative importance are presented for each model with standard deviation and 95% confidence intervals.^41^

### 2.4 Descriptive statistics

Two ways of considering the individuals' sociodemographic characteristics in such models can be used. First, the LC model does not assign individuals to a class, hence the term ‘latent’. However, it generates class membership probabilities for each individual. To assign individuals to each latent class, we can consider the maximum class membership probability (Supporting Information: Appendix 5). Second, the effects of variables of interest can directly be estimated by integrating them into the model specification as being continuous or dummy‐coded (see Boeri et al.^42^ for more details). Although we chose the primer option which consists in assigning an individual relative to his/her maximum class membership probability, we must be cautious because this individual does not really belong to this class.

For purposes of descriptive analysis, relevant tests (i.e., one‐way analysis of variance, Kruskal–Wallis *H*‐test, Bartlett's test for equality of variances, Fisher's exact test and *χ* ^2^ test of independence) were performed to assess differences between classes. The bivariate results are presented in terms of column frequencies. A *p*‐value <.1 was considered significant.

### 2.5 Inclusion criteria

To be included in the analysis, participants had to have LBP for at least 3 months, to have completed some of the choice tasks, to have responded ‘yes’ to the question ‘Did you try to answer the choice exercises as well as possible?’, to have not declared ‘poor’ or ‘very poor’ quality of answers at the question ‘How do you judge the quality of your responses to the choice exercises?’, to do not always chose the first treatment or the second treatment.

## 3 RESULTS

### 3.1 Survey uptake

Among a total of 610 respondents who began the survey, 134 (21.97%) did not achieve any of the choice tasks, 6 (0.98%) declared not responding as well as possible to the choice exercises, 6 (0.98%) declared ‘poor’ or ‘very poor’ quality of answers, 6 (0.98%) had low back pain for less than 3 months, 31 (5.08%) never chose a treatment (opt‐out), 2 (0.33%) always chose the first treatment and 1 (0.16%) always chose the second treatment. This yielded a total of 424 (69.51%) individuals included for analysis.

### 3.2 Descriptive analysis

All descriptive statistics presented here are available in Table 2 (Supporting Information: Appendix 6 for 4 classes). The sample had a mean age of 55 years (±12.62) and was in majority composed of women (F/M ratio of 4.15). About 36.08% were married, 36.56% were retired and 43.40% had a higher education with an average household income of 57,883 CAD (±37,619). Almost all individuals reported having LBP for more than 1 year (98.11%) and the principal diagnoses were lumbar disc herniation (37.74%), facet arthritis (34.91%) and fibromyalgia (35.14%). When answering the survey, individuals had an average level of CLBP of 5.39 (±2.03) over 10. The worst pain and the average level of CLBP in the past 2 weeks were 7.53 (±1.85) over 10 and 5.53 (±1.88) over 10, respectively. Almost one‐third used painkillers every day. Other treatments used were massage therapy sessions (41.98%), infiltrations of corticosteroid products (36.08%) and physiotherapy sessions (34.20%). The average amount of treatment expenditure was 1542 CAD (±3,519) per year and 10.38% were not covered by insurance (public or private one). More than two third of respondents reported having a health problem affecting their quality of life (e.g., pain, 60.38%; tiredness, 50.47%; insomnia, 40.33%; anxiety and stress, 37.50%) and half declared having a fair or poor health status. Both satisfactions with health and life, and the willingness to take risks were 4.12 (±2.42), 5.53 (±2.42) and 4.31 (±2.50) over 10, respectively. Finally, the RMDQ, the ODI, the health utilities of the SF‐6Dv2 and the EQ‐5D‐5L and the EQ‐5D VAS were, respectively, 10.02 (±5.18) over 24 (14 or more indicates a poor outcome), 42.49 (±15.36) over 100 (41–60 indicates severe disability), 0.33 (±0.23) and 0.58 (±0.23) over 1, and 52 (±22.39) over 100.

**Table 2:** Sociodemographic characteristics by latent classes (c=6) and full sample

Sociodemographic characteristics	Class 1	Class 2	Class 3	Class 4	Class 5	Class 6	Total	*p* Value^a^
Observations	48	57	43	67	159	50	424	
Absolute share	11.32%	13.44%	10.14%	15.80%	37.50%	11.79%	100.00%	
Class share	12.60%	14.10%	10.60%	15.70%	34.90%	12.00%	‐	
Gender								
Male	18.75%	19.30%	25.58%	14.93%	15.09%	34.00%	19.34%	**.031**
Female	81.25%	80.70%	72.09%	85.07%	84.91%	64.00%	80.19%	
Intersex	0.00%	0.00%	2.33%	0.00%	0.00%	2.00%	0.47%	
Female/male ratio	4.33	4.18	2.82	5.70	5.63	1.88	4.15	‐
Age (years)								
Mean	54.69	56.53	56.67	54.73	53.11	61.66	55.38	**.002**
Standard deviation	14.14	12.07	11.82	13.95	12	10.42	12.62	
Range	(28–85)	(26–87)	(32–78)	(25–75)	(20–85)	(32–82)	(20–87)	
Less than 35	8.33%	3.51%	2.33%	7.46%	5.03%	2.00%	4.95%	**.004**
35–39	12.50%	3.51%	2.33%	7.46%	6.92%	0.00%	5.90%	
40–44	6.25%	12.28%	11.63%	13.43%	16.98%	4.00%	12.50%	
45–49	6.25%	5.26%	13.95%	2.99%	8.81%	10.00%	7.78%	
50–54	10.42%	14.04%	13.95%	19.40%	15.09%	6.00%	13.92%	
55–59	16.67%	17.54%	18.60%	8.96%	16.35%	12.00%	15.09%	
60–64	10.42%	21.05%	4.65%	2.99%	14.47%	22.00%	12.97%	
65–69	16.67%	12.28%	13.95%	17.91%	9.43%	18.00%	13.44%	
70–74	6.25%	3.51%	11.63%	16.42%	3.14%	16.00%	8.02%	
75 or more	6.25%	7.02%	6.98%	2.99%	3.77%	10.00%	5.42%	
Body mass index (BMI)								
Mean	28	30.97	29.13	29.56	29.05	29.08	29.28	.316
Standard Deviation	5.78	6.92	7.16	6.61	6.66	6.22	6.6	
Range	(18.42–54.69)	(19.44–58.37)	(18.42–62.75)	(19.96–47.18)	(13.71–50.81)	(19.07–49.94)	(13.71–62.75)	
Marital status								
Married	47.92%	35.09%	32.56%	32.84%	34.59%	38.00%	36.08%	.808
Living with a partner	25.00%	28.07%	23.26%	22.39%	24.53%	18.00%	23.82%	
Single	8.33%	14.04%	20.93%	20.90%	19.50%	12.00%	16.98%	
Separated	6.25%	5.26%	6.98%	5.97%	6.92%	6.00%	6.37%	
Divorced	10.42%	10.53%	9.30%	16.42%	11.32%	24.00%	13.21%	
Widowed	2.08%	7.02%	6.98%	1.49%	3.14%	2.00%	3.54%	
Occupational status								
Employed	18.75%	17.54%	23.26%	19.40%	25.79%	4.00%	20.05%	**.083**
Self‐employed	0.00%	1.75%	9.30%	5.97%	6.92%	8.00%	5.66%	
Retired	39.58%	45.61%	34.88%	38.81%	27.04%	52.00%	36.56%	
At home	4.17%	5.26%	4.65%	4.48%	5.03%	6.00%	4.95%	
Student	0.00%	1.75%	0.00%	4.48%	3.77%	0.00%	2.36%	
Unemployed	2.08%	7.02%	2.33%	2.99%	1.26%	4.00%	2.83%	
Sick leave	20.83%	8.77%	11.63%	8.96%	13.21%	18.00%	13.21%	
Parental leave	0.00%	0.00%	0.00%	1.49%	0.00%	0.00%	0.24%	
Other (e.g., disability)	14.58%	12.28%	13.95%	13.43%	16.98%	8.00%	14.15%	
Educational level								
Secondary or less and Diploma of professional studies	25.00%	22.81%	30.23%	26.87%	25.79%	36.00%	27.12%	**.046**
College and CEGEP	35.42%	35.09%	34.88%	29.85%	18.87%	36.00%	28.30%	
Baccalaureate, Masters and PhD	39.58%	42.11%	32.56%	43.28%	53.46%	26.00%	43.40%	
Other	0.00%	0.00%	2.33%	0.00%	1.89%	2.00%	1.18%	
Annual household income (CAD)								
Mean	72,604	63,158	52,965	52,985	56,509	52,900	57,883	**.042**
Standard deviation	36,796	36,421	38,704	34,395	39,345	34,683	37,619	
Range	(40,000–102,500)	(40,000–85,000)	(17,500–75,000)	(30,000–75,000)	(22,500–75,000)	(22,500–75,000)	(30,000–75,000)	
Living with an adult								
Yes	68.75%	71.93%	58.14%	65.67%	66.04%	64.00%	66.04%	.802
No	31.25%	28.07%	41.86%	34.33%	33.96%	36.00%	33.96%	
Underage dependent children (at least one)								
Yes	16.67%	8.77%	18.60%	13.43%	19.50%	12.00%	15.80%	.434
No	83.33%	91.23%	81.40%	86.57%	80.50%	88.00%	84.20%	
Type of residence								
Rural	20.83%	38.60%	20.93%	34.33%	26.42%	34.00%	29.01%	.185
Urban	79.17%	61.40%	79.07%	65.67%	73.58%	66.00%	70.99%	
Owning a home								
Yes	77.08%	68.42%	55.81%	59.70%	66.04%	58.00%	64.62%	.229
No	22.92%	31.58%	44.19%	40.30%	33.96%	42.00%	35.38%	
Smoking								
Yes	14.58%	10.53%	16.28%	10.45%	12.58%	18.00%	13.21%	.807
No	85.42%	89.47%	83.72%	89.55%	87.42%	82.00%	86.79%	
Diagnosis given by a medical doctor								
No diagnosis	0.00%	1.75%	4.65%	5.97%	4.40%	8.00%	4.25%	.364
Muscle and/or ligament sprain	12.50%	7.02%	4.65%	10.45%	7.55%	16.00%	9.20%	.381
Sciatica	27.08%	24.56%	18.60%	31.34%	23.90%	36.00%	26.42%	.394
Lumbar disc herniation	39.58%	42.11%	39.53%	37.31%	33.96%	42.00%	37.74%	.856
Degenerative disc disease	18.75%	24.56%	13.95%	16.42%	16.98%	14.00%	17.45%	.714
Facet arthritis	47.92%	21.05%	44.19%	34.33%	33.33%	36.00%	34.91%	**.069**
Vertebral arthritis or spondylarthrosis	18.75%	21.05%	9.30%	14.93%	8.81%	22.00%	14.15%	**.069**
Spondylolisthesis	8.33%	5.26%	6.98%	4.48%	7.55%	4.00%	6.37%	.914
Deformation (e.g., scoliosis, kyphosis)	14.58%	3.51%	9.30%	13.43%	11.32%	8.00%	10.38%	.381
Osteoporosis	2.08%	8.77%	6.98%	8.96%	7.55%	8.00%	7.31%	.758
Osteoporosis with spinal fracture	0.00%	0.00%	2.33%	1.49%	0.00%	4.00%	0.94%	**.077**
Fracture or dislocation of the spine	0.00%	0.00%	6.98%	1.49%	1.26%	6.00%	2.12%	**.046**
Autoimmune inflammatory disease	10.42%	1.75%	6.98%	2.99%	3.77%	2.00%	4.25%	.266
Fibromyalgia	35.42%	42.11%	27.91%	32.84%	35.22%	36.00%	35.14%	.795
Other diagnosis	27.08%	24.56%	32.56%	22.39%	24.53%	24.00%	25.24%	.890
How long are you suffering from low back pain?								
Between 3 months and 1 year	0.00%	1.75%	6.98%	0.00%	2.52%	0.00%	1.89%	.133
More than 1 year	100.00%	98.25%	93.02%	100.00%	97.48%	100.00%	98.11%	
Today low back pain (ranging from 0 to 10)								
Mean	5.71	5.60	5.65	5.00	5.17	5.86	5.39	**.090**
Standard deviation	1.83	2.11	2.11	2.06	1.98	2.06	2.03	
Range	(2–9)	(0–10)	(2–10)	(0–9)	(0–10)	(1–10)	(0–10)	
Worst level of low back pain in the past 2 weeks (ranging from 0 to 10)								
Mean	7.83	7.56	7.49	7.34	7.45	7.78	7.53	.666
Standard deviation	1.73	2.07	1.82	2.13	1.69	1.87	1.85	
Range	(4–10)	(2–10)	(2–10)	(0–10)	(4–10)	(3–10)	(0–10)	
Average level of low back pain in the past 2 weeks (ranging from 0 to 10)								
Mean	5.75	5.46	5.72	5.4	5.35	6.02	5.53	.267
Standard deviation	1.54	1.88	1.92	2.02	1.83	2.07	1.88	
Range	(2–10)	(1–10)	(1–10)	(0–10)	(1–10)	(1–10)	(0–10)	
Frequency of use of painkillers								
Several times a day	33.33%	29.82%	51.16%	22.39%	22.64%	40.00%	29.72%	.196
Every day	33.33%	33.33%	23.26%	31.34%	31.45%	32.00%	31.13%	
Several times a week	18.75%	5.26%	9.30%	16.42%	18.24%	10.00%	14.39%	
Once a week	2.08%	3.51%	4.65%	2.99%	1.26%	2.00%	2.36%	
Several times a month	4.17%	8.77%	4.65%	5.97%	9.43%	2.00%	6.84%	
Once a month	2.08%	5.26%	4.65%	0.00%	3.77%	2.00%	3.07%	
Several times a year	2.08%	7.02%	2.33%	7.46%	6.92%	8.00%	6.13%	
Once a year	0.00%	1.75%	0.00%	1.49%	0.63%	0.00%	0.71%	
Never	4.17%	5.26%	0.00%	11.94%	5.66%	4.00%	5.66%	
Treatments for reducing pain other than painkillers								
Homoeopathic products	2.08%	8.77%	2.33%	2.99%	10.69%	10.00%	7.31%	.124
Infiltration of corticosteroid products	62.50%	12.28%	53.49%	34.33%	31.45%	40.00%	36.08%	**<.001**
Chiropractic sessions	16.67%	15.79%	4.65%	17.91%	13.84%	12.00%	13.92%	.467
Physiotherapy sessions	43.75%	29.82%	27.91%	26.87%	39.62%	28.00%	34.20%	.172
Osteopathy sessions	12.50%	26.32%	23.26%	23.88%	28.93%	24.00%	24.76%	.355
Occupational therapy sessions	10.42%	3.51%	4.65%	1.49%	5.03%	16.00%	6.13%	**.027**
Psychotherapy sessions	18.75%	5.26%	13.95%	5.97%	13.21%	10.00%	11.32%	.176
Reflexology sessions	0.00%	1.75%	2.33%	2.99%	3.77%	6.00%	3.07%	.675
Massage therapy sessions	50.00%	35.09%	44.19%	46.27%	41.51%	36.00%	41.98%	.590
Yoga sessions	12.50%	19.30%	6.98%	10.45%	21.38%	12.00%	15.80%	.103
Stretching sessions	16.67%	12.28%	23.26%	14.93%	20.75%	20.00%	18.40%	.638
Acupuncture sessions	10.42%	7.02%	13.95%	11.94%	15.72%	18.00%	13.44%	.529
Cupping sessions	0.00%	1.75%	0.00%	2.99%	5.03%	0.00%	2.59%	.291
Infrared frequency sessions	4.17%	0.00%	0.00%	1.49%	2.52%	0.00%	1.65%	.522
Bodybuilding	6.25%	14.04%	4.65%	7.46%	12.58%	16.00%	10.85%	.322
Endurance activities (aerobic)	10.42%	7.02%	6.98%	5.97%	11.32%	6.00%	8.73%	.774
Consumption of medical cannabis	12.50%	15.79%	13.95%	20.90%	16.98%	10.00%	15.80%	.663
Others	37.50%	28.07%	34.88%	29.85%	32.08%	36.00%	32.55%	.892
Treatment expenditure per year (CAD)								
Mean	2107.29	961.93	1770.93	1905.82	1433.45	1323.60	1542.26	.547
Standard deviation	4821.23	1245.30	3602.08	6679.35	1648.68	1364.97	3519.43	
Range	(0–34,000)	(0–7000)	(0–20,000)	(0–55,000)	(0–10,000)	(20–8000)	(0–55,000)	
Insurance								
RAMQ (carte soleil)	39.58%	36.84%	39.53%	44.78%	33.96%	46.00%	38.68%	**.091**
Private insurance	60.42%	57.89%	46.51%	44.78%	52.20%	42.00%	50.94%	
No insurance	0.00%	5.26%	13.95%	10.45%	13.84%	12.00%	10.38%	
Do you suffer from a disease or a physical or mental problem that reduces your quality of life (e.g., diabetes, cancer, osteoarthritis)?								
Yes	75.00%	68.42%	83.72%	65.67%	64.78%	60.00%	67.92%	.138
No	25.00%	31.58%	16.28%	34.33%	35.22%	40.00%	32.08%	
Tiredness	52.08%	49.12%	60.47%	50.75%	49.69%	44.00%	50.47%	.947
Insomnia	52.08%	38.60%	55.81%	26.87%	37.74%	44.00%	40.33%	**.049**
Pain	70.83%	57.89%	72.09%	61.19%	57.23%	52.00%	60.38%	.667
Anxiety/stress	39.58%	31.58%	51.16%	40.30%	35.22%	34.00%	37.50%	.759
Depression	27.08%	19.30%	30.23%	26.87%	24.53%	18.00%	24.29%	.833
Other mental disorder	6.25%	3.51%	6.98%	4.48%	3.14%	4.00%	4.25%	.925
Osteoarthritis	52.08%	47.37%	58.14%	38.81%	40.88%	34.00%	43.63%	.763
Arthritis	10.42%	8.77%	18.60%	11.94%	8.81%	14.00%	11.08%	.673
Unintentional injury	2.08%	3.51%	4.65%	4.48%	5.03%	2.00%	4.01%	.949
Musculoskeletal problem	25.00%	17.54%	18.60%	20.90%	21.38%	18.00%	20.52%	.838
Disease of the central nervous system	6.25%	5.26%	13.95%	4.48%	6.92%	6.00%	6.84%	.793
Thyroid problem	8.33%	14.04%	9.30%	20.90%	13.84%	4.00%	12.74%	**.057**
Other endocrine problem	6.25%	1.75%	2.33%	5.97%	3.77%	0.00%	3.54%	.505
Genital‐urinary problem	6.25%	7.02%	9.30%	5.97%	5.66%	4.00%	6.13%	.991
Hypertension	22.92%	31.58%	37.21%	25.37%	17.61%	28.00%	24.53%	.136
Cardiac disease	4.17%	3.51%	13.95%	5.97%	1.26%	6.00%	4.48%	**.033**
Stroke	2.08%	0.00%	2.33%	0.00%	0.63%	0.00%	0.71%	.473
Digestive disorder	10.42%	15.79%	27.91%	26.87%	16.98%	12.00%	18.16%	**.097**
Other gastrointestinal problem	16.67%	19.30%	16.28%	14.93%	11.32%	10.00%	13.92%	.777
Diabetes	8.33%	19.30%	27.91%	19.40%	8.18%	22.00%	15.09%	**.006**
Cancer/tumour	0.00%	1.75%	0.00%	7.46%	0.63%	2.00%	1.89%	**.021**
Chronic obstructive pulmonary disease (COPD)	0.00%	0.00%	0.00%	1.49%	1.89%	2.00%	1.18%	.790
Other breathing problems (asthma, emphysema)	6.25%	5.26%	4.65%	17.91%	11.95%	14.00%	10.85%	**.031**
Other medical disorder	27.08%	31.58%	20.93%	20.90%	31.45%	34.00%	28.54%	**.044**
Health status								
Mean	3.58	3.42	3.81	3.51	3.39	3.62	3.50	**.093**
Standard deviation	0.90	1.03	0.82	0.84	0.89	0.83	0.90	
Range	(2–5)	(2–5)	(2–5)	(1–5)	(1–5)	(2–5)	(1–5)	
Excellent	0.00%	0.00%	0.00%	1.49%	1.26%	0.00%	0.71%	**.066**
Very good	6.25%	21.05%	6.98%	7.46%	13.21%	8.00%	11.32%	
Good	50.00%	35.09%	23.26%	40.30%	41.51%	36.00%	38.92%	
Fair	22.92%	24.56%	51.16%	40.30%	33.33%	42.00%	34.91%	
Poor	20.83%	19.30%	18.60%	10.45%	10.69%	14.00%	14.15%	
Satisfaction with health								
Mean	3.96	4.26	3.26	4.27	4.30	4.10	4.12	.215
Standard deviation	2.18	2.61	2.32	2.44	2.4	2.44	2.42	
Range	(0–8)	(0–9)	(0–8)	(0–9)	(0–10)	(0–9)	(0–10)	
Satisfaction with life								
Mean	5.73	5.63	5.33	5.55	5.74	4.72	5.53	.187
Standard deviation	2.01	2.44	2.84	2.26	2.38	2.62	2.42	
Range	(1–9)	(0–10)	(0–10)	(0–10)	(0–10)	(0–9)	(0–10)	
Willingness to take risks								
Mean	4.42	4.00	4.07	4.33	4.38	4.54	4.31	.863
Standard deviation	2.43	2.51	2.54	2.34	2.53	2.67	2.5	
Range	(0–9)	(0–9)	(0–8)	(0–8)	(0–9)	(0–10)	(0–10)	
Roland‐Morris Disability Questionnaire^b^								
Mean	9.76	9.69	10.46	10.32	9.58	11.38	10.02	.475
Standard deviation	4.94	4.65	4.92	5.43	5.32	5.37	5.18	
Range	(1–22)	(1–19)	(1–20)	(1–23)	(1–23)	(1–22)	(1–23)	
Oswestry Disability Index^c^								
Mean	44.45	43.57	45.74	42.65	38.89	48.39	42.49	**.005**
Standard deviation	12.21	16.20	13.68	15.38	16.27	13.14	15.36	
Range	(20–66)	(6–80)	(14–92)	(6–78)	(6–76)	(24–86)	(6–92)	
Minimal disability (0–20)	2.27%	14.89%	2.63%	8.06%	16.90%	0.00%	10.16%	**<.001**
Moderate disability (21–40)	38.64%	25.53%	31.58%	35.48%	40.85%	24.39%	35.03%	
Severe disability (41–60)	43.18%	48.94%	60.53%	43.55%	31.69%	63.41%	43.58%	
Crippled (61–80)	15.91%	10.64%	2.63%	12.90%	10.56%	9.76%	10.70%	
Bed‐bound (81–100)	0.00%	0.00%	2.63%	0.00%	0.00%	2.44%	0.53%	
SF‐6Dv2^d^								
Mean	0.337	0.343	0.246	0.332	0.356	0.306	0.332	.200
Standard deviation	0.218	0.220	0.271	0.234	0.223	0.234	0.231	
Range	(−0.245–0.703)	(−0.191–0.840)	(−0.373–0.759)	(−0.317–0.843)	(−0.320–0.882)	(−0.320–0.799)	(−0.373–0.882)	
EQ‐5D‐5L^e^								
Mean	0.563	0.565	0.519	0.613	0.597	0.515	0.575	.137
Standard deviation	0.200	0.230	0.257	0.226	0.227	0.230	0.229	
Range	(0.088–0.885)	(0.129–0.885)	(−0.072–0.860)	(0.045–0.904)	(−0.044–0.904)	(−0.064–0.828)	(−0.072–0.904)	
EQ‐5D‐5L‐VAS								
Mean	46.42	53.79	51.57	55.21	54.99	41.27	52.00	**.006**
Standard deviation	18.81	21.63	23.70	24.33	21.77	21.51	22.39	
First quartile	33	36	31	36	40	24	34	
Median	46	56	51	59	54	40	53	
Third quartile	59	68	68	72	71	58	69	
Range	(11–89)	(0–88)	(0–100)	(5–100)	(0–100)	(2–83)	(0–100)	

*Note*: Bold values are statistiscally significant at *p* < .1 level.

Abbreviations: CEGEP, Collège d'enseignement général et professionnel; QALY, Quality‐Adjusted Life‐Year; RAMQ, Régie de l'assurance maladie du Québec.

^a^ The *p*‐values refer to tests between classes using the one‐way analysis of variance, Kruskal–Wallis *H*‐test, Bartlett's test for equality of variances, Fisher's exact test and *χ* ^2^ test of independence.

^b^ The Roland‐Morris Disability Questionnaire is a 24‐item questionnaire measuring self‐assessed back pain with a yes/no format and ranging from 0 (no back pain) to 24 (worst back pain).

^c^ The Oswestry Disability Index is a 10‐item questionnaire with a 6‐point Likert scaling and rescaled from 0 to 100.

^d^ The Short Form 6‐Dimension version 2 (SF‐6Dv2) is a 6‐dimension generic health‐related quality of life questionnaire.

^e^ The EuroQol 5‐Dimension 5‐Level (EQ‐5D‐5L) is a 5‐dimension generic health‐related quality of life questionnaire.

### 3.3 Preference analysis

The ranking of the attributes' conditional relative importance was the same in two models (HB and LC). It was as follows: (1) treatment modality, (2) pain reduction, (3) difficulties with daily activities, (4) duration effectiveness, (5) sleep problems, (6) onset of treatment effectiveness and (7) knowledge of his/her body and pain location (Table 3).

**Table 3:** Hierarchical Bayesian and Latent Class Logit models with the conditional relative importance of attributes

Part‐worth utilities								
Attribute	Bayesian Hierarchical model				Latent Class model			
	Standardized utilities^a^	Standard deviation	Lower 95% CI	Upper 95% CI	Standardized utilities^a^	Standard deviation	Lower 95% CI	Upper 95% CI
None^b^	−45.63	159.19	−60.78	−30.48	−129.97	254.71	−154.22	−105.73
Treatment modality								
Corticosteroid injections	−52.18	121.81	−63.78	−40.59	−63.54	147.25	−77.56	−49.53
Supervised body‐mind physical activities	23.30	61.09	17.48	29.11	24.94	57.25	19.49	30.39
Supervised sports physical activities	6.15	89.92	−2.41	14.71	20.84	68.64	14.30	27.37
Physical manipulations	52.57	49.66	47.85	57.30	59.39	28.24	56.70	62.08
Self‐management courses	21.47	59.66	15.80	27.15	20.62	47.74	16.07	25.16
Psychotherapy	−51.31	76.19	−58.56	−44.06	−62.24	60.73	−68.02	−56.46
Pain reduction								
None to very slight	−61.18	40.95	−65.07	−57.28	−69.92	50.02	−74.69	−65.16
Slight	−16.69	25.55	−19.12	−14.25	−18.81	21.77	−20.89	−16.74
Reduced by half	33.67	23.75	31.41	35.93	40.25	18.55	38.49	42.02
No pain	44.19	44.36	39.97	48.41	48.48	47.76	43.94	53.03
Onset of treatment efficacy								
1 month	12.97	30.37	10.08	15.87	16.29	18.57	14.53	18.06
6 months	7.18	16.08	5.65	8.71	3.76	10.77	2.73	4.78
12 months	−20.15	28.58	−22.87	−17.43	−20.05	20.07	−21.96	−18.14
Effectiveness duration								
Effective for 2 months	−26.61	28.10	−29.28	−23.93	−24.75	28.68	−27.48	−22.02
Effective for 6 months	1.83	25.50	−0.59	4.26	−2.43	11.80	−3.55	−1.30
Effective for 12 months	24.78	23.89	22.50	27.05	27.17	20.31	25.24	29.11
Difficulties with daily activities								
As many difficulties as before	−42.64	29.23	−45.42	−39.85	−45.61	29.69	−48.44	−42.78
Fewer difficulties as before	17.30	19.41	15.45	19.15	18.97	19.98	17.07	20.87
No difficulties	25.33	23.58	23.09	27.58	26.64	16.49	25.07	28.21
Sleep problems								
As many problems as before	−25.25	32.14	−28.31	−22.19	−24.71	30.48	−27.61	−21.81
Fewer problems than before	14.45	18.99	12.65	16.26	18.70	10.62	17.69	19.71
No problems	10.79	30.61	7.88	13.71	6.00	36.97	2.48	9.52
Knowledge of his/her body and pain location								
Same knowledge as before	−8.74	19.77	−10.62	−6.86	−11.36	10.94	−12.40	−10.32
Knowledge somewhat better than before	−1.86	17.41	−3.51	−0.20	1.86	15.63	0.37	3.35
Knowledge much better than before	10.59	19.34	8.75	12.44	9.50	20.82	7.52	11.48
Observations	424				424			
McFadden *R* ^2^	30.01				26.04			
*χ* ^2^	2830.41				2730.48			
Log‐likelihood	−3701.45				−3876.09			
Log‐likelihood (null)	−5233.01				−5243.68			
AIC	7901.67				7994.88			
BIC	8567.87				8764.89			

Relative weight of attributes								
Attribute	Bayesian Hierarchical model				Latent Class model			
	Weight (%)	Standard deviation	Lower 95% CI	Upper 95% CI	Weight (%)	Standard deviation	Lower 95% CI	Upper 95% CI
Treatment modality	34.79	11.94	33.65	35.93	36.06	12.74	34.85	37.28
Pain reduction	18.73	7.13	18.05	19.41	20.43	8.32	19.63	21.22
Onset of treatment efficacy	8.60	5.12	8.11	9.09	6.65	3.59	6.31	6.99
Effectiveness duration	10.06	4.76	9.61	10.52	9.96	2.02	9.77	10.15
Difficulties with daily activities	11.71	5.41	11.20	12.23	11.40	5.16	10.91	11.89
Sleep problems	10.05	4.60	9.61	10.48	10.07	4.34	9.66	10.48
Knowledge of his/her body and pain location	6.06	3.17	5.76	6.36	5.43	3.06	5.14	5.72

Abbreviations: AIC, Akaike Information Criterion; BIC, Bayesian Information Criterion.

^a^ A value close to zero indicates an absence of preference: the further the value is from zero, the greater the preference.

^b^ Coefficient of the opt‐out option.

According to the HB model, the conditional relative importance of these attributes ranged from 34.79% (±11.94) to 6.06% (±3.17) whereas it was from 36.06% to 5.43% in the LC model, showing little difference (Table 3). For treatments in the HB model, the standardized utility values (i.e., the personal satisfaction provided by chosen treatments) were as follows: −52.18 (±121.81) for corticosteroid injections, 23.30 (±61.09) for supervised body‐mind physical activities, 6.15 (±89.92) for supervised sports physical activities, 52.57 (±49.66) for physical manipulations, 21.47 (±59.66) self‐management courses and −51.31 (±76.19) for psychotherapy, thus showing on average a high disutility for corticosteroid injections and psychotherapy.

However, in the LC model, classes 1, 3 and 6 (i.e., one‐third of the full sample) expressed a high utility for corticosteroid injections, and only class 3 (i.e., one‐tenth of the full sample) expressed a utility for psychotherapy (Table 4) (Supporting Information: Appendix 7 for 4 classes). Some discrepancies were identified in the utility levels, particularly in class 6 (e.g., they expressed a high disutility as the level of treatment effectiveness increased or when the level of sleep problems decreased), which was considered as a limit.

**Table 4:** Zero‐centred utility values produced by the latent class analysis (c=6) and conditional relative importance of attributes

Latent class logit						
Attributes (standardized utilities)^a^	Class 1	Class 2	Class 3	Class 4	Class 5	Class 6
None^b^	−266.03	30.16	−88.43	101.02	−400.98	333.90
Treatment modality						
Corticosteroid injections	98.32	−322.34	148.46	−3.90	−159.76	99.49
Supervised body‐mind physical activities	−35.28	141.11	−43.22	−12.99	54.71	−31.82
Supervised sports physical activities	75.37	46.56	−175.12	10.47	39.84	83.49
Physical manipulations	42.70	82.24	−5.58	93.21	60.74	21.29
Self‐management courses	−52.13	124.36	29.80	−39.60	29.61	7.70
Psychotherapy	−129.00	−71.93	45.66	−47.20	−25.16	−180.16
Pain reduction						
None to very slight	−0.78	−20.59	−104.51	−93.70	−109.22	33.74
Slight	14.81	−41.14	4.84	−36.75	−29.80	19.97
Reduced by half	15.39	26.07	44.25	64.08	46.12	−1.47
No pain	−29.43	35.67	55.42	66.38	92.91	−52.25
Onset of treatment efficacy						
1 month	43.08	14.38	−24.28	40.57	10.89	1.35
6 months	12.57	−7.24	−1.08	−12.09	15.59	−1.43
12 months	−55.65	−7.14	25.36	−28.48	−26.47	0.08
Duration effectiveness						
Effective for 2 months	−24.95	−31.63	−14.28	−44.98	−36.30	55.20
Effective for 6 months	−15.04	11.67	−3.18	10.90	−1.21	−26.97
Effective for 12 months	39.99	19.96	17.46	34.08	37.50	−28.24
Difficulties with daily activities						
As many difficulties as before	−78.14	−8.70	−18.41	−77.50	−52.76	16.43
Fewer difficulties as before	30.38	8.42	−22.07	42.03	28.83	−13.53
No difficulties	47.76	0.28	40.48	35.47	23.92	−2.90
Sleep problems						
As many problems as before	−8.47	−24.80	−27.24	−62.79	−31.52	55.11
Fewer problems than before	32.87	9.40	16.12	22.80	6.96	32.76
No problems	−24.39	15.40	11.12	39.99	24.56	−87.87
Knowledge of his/her body and pain location						
Same knowledge as before	−32.84	−12.15	14.41	−4.46	−14.32	−7.73
Knowledge somewhat better than before	−15.36	−4.90	0.39	−12.32	4.80	41.46
Knowledge much better than before	48.20	17.05	−14.80	16.78	9.52	−33.72
Number of observations	48	57	43	67	159	50
Absolute share (%)	11.32	13.44	10.14	15.80	37.50	11.79
Class share (%)	12.60	14.10	10.60	15.70	34.90	12.00
Observations	424					
McFadden *R* ^2^	26.036					
*χ* ^2^	2730.48					
Log‐likelihood	−3876.09					
Log‐likelihood (null)	−5243.68					
AIC	7994.88					
BIC	8764.89					
Conditional relative importance of attributes (%)						
Treatment modality	32.47	66.21	46.23	20.06	31.50	39.95
Pain reduction	6.40	10.97	22.85	22.87	28.87	12.28
Onset of treatment efficacy	14.10	3.09	7.09	9.87	6.01	0.40
Duration effectiveness	9.28	7.37	4.53	11.29	10.54	11.92
Difficulties with daily activities	17.99	2.45	8.94	17.08	11.66	4.28
Sleep problems	8.18	5.74	6.19	14.68	8.01	20.43
Knowledge of his/her body and pain location	11.58	4.17	4.17	4.16	3.41	10.74

Abbreviations: AIC, Akaike Information Criterion; BIC, Bayesian Information Criterion.

^a^ A value close to zero indicates an absence of preference: the further the value is from zero, the greater the preference.

^b^ Coefficient of the opt‐out option.

With regard to other attributes, the HB model (i.e., preferred model) indicated that respondents were able to wait up to 6–12 months until their preferred treatment started to be effective and wanted an effectiveness duration of at least 6 months. An improvement from ‘fewer difficulties as before’ to ‘no difficulties’ in their daily life activities, as well as an improvement to ‘fewer problems than before’ in their sleep problems were appreciated whereas the attribute ‘knowledge of his/her body and pain location’ had a minor effect, that is, its improvement had to be high to influence the preference.

From these results, we can observe that the treatment modality had a great importance beside the other attributes. All attributes were always ranked at the same position. Among the treatment modality, corticosteroid injections and psychotherapy were generally rejected whereas physical manipulations were preferred followed by the supervised body‐mind physical activities and the self‐management courses. Supervised sports physical activities had a positive but slight effect, and its weight was smaller in the HB model than in the logit and CL models. Graphical representations of part worth utilities are given in Supporting Information: Appendix 8 and Kernel densities using Epanechnikov kernel function of the HB model' part worth utilities are provided in Supporting Information: Appendix 9 with one‐sample multivariate tests of means ($H_{0}:\mu =0$).

### 3.4 Explaining preference heterogeneity

The LC model estimated preferences that are heterogeneous between classes and homogeneous within classes. It allowed us to examine significant statistical differences (at *p* < .10) in the sociodemographic characteristics of the six classes (Table 2). Compared to the other classes, class 1 had a higher household income (72,604 CAD, ±36,796, *p* = .042), benefited more from injections of corticosteroid products (62.5%, *p* < .001), spent more money to treat CLBP (2107 CAD, ±4821, *p* = .547) and had more other problems affecting quality of life (75%, *p* = .138). Class 2 benefited less from corticosteroid injections (12.28%, *p* < .001) and spent less money to treat their CLBP (961 CAD, ±1245, *p* = .547). Class 3 was made up of more men (25.58%, *p* = .031), although the ratio of men over women remained very unbalanced (1 man for 2.8 women against 1 man for 4.1 women in the full sample), more individuals with pain lasting from 3 months to 1 year at the time of the survey (7%, *p* = .133), more often benefited from corticosteroid injections (53.49%, *p* < .001), did less yoga (6.98%, *p* = .103), presented more diseases or physical or mental problems affecting their quality of life (83.72%, *p* = .138), and reported having a better self‐reported health state (3.81 over 5, ±0.82, *p* = .093). Class 4 was more feminine (85.1%, *p* = .031), less in pain when responding to the survey (5 over 10, ±2.06, *p* = .090) and spent more money to treat CLBP (1906 CAD, ±6679, *p* = .547). It was also the class with the most cancer (7.46%, *p* = .021) and respiratory problems reported (17.91%, *p* = .021). Class 5 was slightly younger (53, *p* = .002) and more educated (*p* = .046). Finally, class 6 was the oldest (62, *p* = .002), the one with the most of men (34%, *p* = .031), the least educated (*p* = .046), more diagnosed with vertebral arthritis or spondylarthrosis (22%, *p* = .069) and the one being the most in pain when answering the survey (5.86 over 10, ±2.06, *p* = .090), but also having the fewest other problems affecting their quality of life (60%, *p* = .138). Respondent's personal ranking of attributes and treatment modalities are presented in Supporting Information: Appendix 10.

No significant difference was found among classes on the choice certainty score, the difficulty and the quality of responses (Supporting Information: Appendix 11). The feeling of annoyance was almost significant (*p* = .102), individuals in class 1 being more annoyed compared to other classes (39.13%). Only the number of dimensions/attributes considered by individuals for the choice exercises was significant (*p* = .004), with classes 1, 3 and 6 who considered less dimensions than the average. Class 6 rejected more than other classes the treatments offered in the choice‐based exercise (7.3 opt‐out selected over 11 possibilities [note that respondents who refused 12 times were deleted], ±2.91, *p* < .001), whereas classes 1 and 5 strongly accepted them (0.73, ±1.03 and 0.17, ±0.41, respectively). About 13.68% and 34.67% failed to the rationality test and to the temporal consistency test, respectively, and differences between latent classes were significant (*p* < .001 and *p* = .003, respectively). Bivariate analyses of the hierarchical clustering derived from the HB model are provided in Supporting Information: Appendix 12.

The bivariate analyses were enhanced by a multinomial logistic regression on the classes (Supporting Information: File 1) with respondent characteristics as arguments. The results indicated that only some variables explained the class memberships. This is an important limitation that is probably due to a very limited number of observations in each class to allow a consistent multivariate analysis.

## 4 DISCUSSION

Using a DCE, we assessed patients' preferences for CLBP nonsurgical treatments. Our segmentation results showed a diversity of preferences. Indeed, classes 1, 3 and 6 preferred the corticosteroid injections (class 3 also preferred psychotherapy), class 2 preferred supervised body‐mind physical activities and self‐management courses and classes 4 and 5 preferred physical manipulations. More generally, patients (as a whole) expressed a disutility for corticosteroid injections and psychotherapy. They all differed in their sociodemographic status, health conditions and in their experiences with CLBP, although in a multivariate analysis this was not a predictor for class membership. These results were somewhat corroborated with a simple question of attributes self‐ranking and with the four‐item questionnaire of Dima et al.^37^ (Table 5). According to these findings, Quebec health authorities should generalize access to physical manipulations, develop body‐mind and self‐management programmes, and in some specific cases, propose corticosteroid injections and psychotherapies.

**Table 5:** Four‐item Low Back Pain Treatment Beliefs Questionnaire (LBP‐TBQ) scores

Four‐item LBP‐TBQ	Proposed treatment					
	Corticosteroid injections	Supervised body‐mind physical activities	Supervised sports physical activities	Physical manipulations	Self‐management courses	Psychotherapy
Taking/having this treatment for back pain makes a lot of sense (1–5)						
Mean	3.01	3.89	3.64	4.16	4.18	3.76
Standard deviation	1.32	0.99	1.11	0.96	0.76	0.96
Range	(1–5)	(1–5)	(1–5)	(1–5)	(1–5)	(1–5)
Strongly disagree	19.17%	3.06%	6.11%	2.78%	1.11%	2.22%
Disagree	15.00%	4.72%	8.61%	3.06%	0.56%	6.67%
Neither agree nor disagree	25.28%	21.94%	23.33%	13.06%	13.06%	27.50%
Agree	26.94%	40.28%	38.61%	38.06%	49.44%	40.28%
Strongly agree	13.61%	30.00%	23.33%	43.06%	35.83%	23.33%
I think this treatment is pretty useless for people with back pain (1–5)^a^						
Mean	2.66	2.44	2.46	2.29	2.33	2.54
Standard deviation	1.29	1.23	1.21	1.30	1.26	1.22
Range	(1–5)	(1–5)	(1–5)	(1–5)	(1–5)	(1–5)
Strongly disagree	23.61%	26.67%	26.39%	37.78%	32.78%	24.44%
Disagree	24.44%	31.39%	29.44%	23.89%	29.72%	26.11%
Neither agree nor disagree	26.11%	19.44%	22.22%	17.78%	16.94%	28.61%
Agree	14.44%	15.83%	15.56%	12.78%	13.33%	12.78%
Strongly agree	11.39%	6.67%	6.39%	7.78%	7.22%	8.06%
I have concerns about taking/having this treatment for my back pain (1–5)^a^						
Mean	3.33	2.22	2.63	2.19	1.99	2.27
Standard deviation	3.33	2.22	2.63	2.19	1.99	2.27
Range	1.41	1.10	1.29	1.14	0.99	1.12
Strongly disagree	15.56%	30.56%	23.61%	33.61%	36.94%	30.56%
Disagree	15.28%	33.33%	28.33%	32.22%	36.94%	29.72%
Neither agree nor disagree	15.83%	23.33%	18.89%	19.44%	17.78%	26.11%
Agree	27.50%	8.89%	20.00%	10.56%	6.39%	9.17%
Strongly agree	25.83%	3.89%	9.17%	4.17%	1.94%	4.44%
I am confident this treatment would be a suitable treatment for my back pain (1–5)						
Mean	2.93	3.82	3.54	4.03	4.05	3.67
Standard deviation	2.93	3.82	3.54	4.03	4.05	3.67
Range	1.39	1.03	1.20	0.99	0.85	1.07
Strongly disagree	24.72%	4.72%	8.61%	2.78%	1.67%	5.00%
Disagree	11.39%	5.00%	11.67%	6.11%	2.78%	6.94%
Neither agree nor disagree	25.83%	20.00%	19.17%	12.78%	15.28%	28.06%
Agree	22.78%	44.44%	38.33%	42.50%	49.44%	36.39%
Strongly agree	15.28%	25.83%	22.22%	35.83%	30.83%	23.61%

^a^ The lower the score is, the more appreciated the treatment modality. For each treatment, Cronbach's *α* were equal to .818, .737, .766, .738, .652 and .712, respectively. See Supporting Information: File 2 for details.

We found seven DCEs on LBP treatments conducted in the United States,^14^ the United Kingdom,^19^, ^20^ Denmark,^15^ Sweden,^17^ Australia^18^ and China.^16^ Contrary to these DCEs mostly focussing on a few specific treatments, our study mobilized six different first‐line treatments for the management of CLBP. For example, Kløjgaard et al.^15^ opposed generic surgical to nonsurgical treatments and Turk et al.^14^ only included oral medication and injection as treatment modalities. About three types of exercises were considered by Aboagye et al.^17^ and a mix of medication, education and physical therapy were considered by Yi et al.^19^ Comparing six different treatments including psychological and various physical therapies was useful for considering the patients' preferences about CLBP treatments, particularly in an academic hospital to help organize the care pathways (e.g., to identify imbalance in resource requirements, potential breakdown of services). In addition, beyond preferences, differences in accessibility and/or affordability for some treatments may remain, particularly surgical ones, and such a study may help healthcare professionals and managers to better consider the balance between the needs of patients and what can be offered to them. While patients granted the highest conditional relative importance to treatments and manifested a high preference for physical manipulations and body‐mind physical activities in our study, these types of treatment were not significantly retained by the participants in the study of Yi et al.^19^ However, they were the second most important attributes in the study of Aboagye et al.^17^ Chen et al.^16^ and Kløjgaard et al.^15^ were the only studies to examine the effectiveness duration and treatment effect on daily activities and found that these attributes were significantly retained by participants, as in our study. Both also found that the effectiveness of pain relief was a major attribute.

Differences between the six classes from the LC model could explain some preferences for the treatment choices, in particular why classes 1, 3 and 6 expressed a strong preference for corticosteroid injections. Including heterogeneity in the analysis, the LC model allowed us to consider heterogeneity between classes. We described six classes and found relevant discriminant preferences and sociodemographic characteristics. Ferreira et al.^18^ and Walsh et al.^20^ also derived different classes (n=4) from an LC model with their own preferences and sociodemographic characteristics. For instance, the primer found that the elderly and men with lower socioeconomic status preferred exercise less whereas all other classes preferred this programme. Individuals already doing exercise also had a preference for a programme with higher frequency and lower cost. Walsh et al. found that improvement in symptom control and risk of physical dependency were of utmost importance in patients suffering from osteoarthritis and CLPB and defined four distinct groups as ‘efficacy‐focused’, ‘cost‐averse’, ‘physical‐dependence‐averse’ and ‘needle‐averse’. Yi et al.,^19^ Kløjgaard et al.^15^ and Turk et al.^14^ also performed subgroups' analyses. They found the following, respectively: (1) significant differences between pain grades and preferences for the content (i.e., education, physical therapy, medicines and coping with pain) and for the type of provider of the programme (i.e., nurse, physiotherapist, general practitioner and psychologist), (2) some differences between sociodemographic characteristics and subgroups (i.e., age, gender, surgery experiences) and (3) few significant differences between the respondent condition (i.e., osteoarthritis, CLBP or both), the opioids use, the painful experiences or the locus of control questions on the MHLC Scale‐Form C. However, all studies had different aims, with their own attributes and levels. Thus, the comparisons with common attributes may not be so relevant.

The biggest strength of our study was the number of treatment modalities, which allowed us to consider a variety of preferences. Indeed, this is the only study that considered six nonsurgical CLBP treatments. Moreover, preferences were estimated via three models and attributes were mostly coherent in their levels, when there was gradation. Also, almost 70% of the total number of respondents was included in the analysis showing the attractivity and quality of the survey. Individuals with longer CLBP were more willing to answer with 98.11% of respondents suffering for more than 1 year. Another strength of the study is that we followed a mixed method with a qualitative phase for the design of the DCE.^13^, ^38^ In addition, the survey was administered online and made it possible to relieve the social desirability bias.^43^ Nevertheless, an online survey may create a self‐selection bias, targeting only a certain population who have an interest and capacities and access to information technologies to answer the survey. However, this bias may be limited by the fact that 93%of Quebec households have access to the internet.^44^ Another limitation referred to the DCE design. Using seven attributes may lead to a cognitive burden and cause confusion in individuals while making their choices. Indeed, it has been shown that increasing the number of attributes increases the likelihood of self‐simplifying the exercise by heuristics.^45^ However, the number of attributes in this study is in line with other studies.^14^, ^15^, ^16^, ^17^, ^18^, ^19^ In addition, since a very simple description of the attributes, was provided (Table 1), it could induce a lack of comprehension from the participants if not carefully considered. Yet, participants were part of an association dealing with chronic pain. Almost all were suffering from CLBP for more than 1 year, used painkillers, and had substantial experiences with treatments (Table 2). We are thus confident that participants were knowledgeable about the treatments. Also to note that as Ferreira et al.^18^ discussed in the case of exercise programmes, it must be emphasized that considering the patient's preferences, he/she could prefer a less effective treatment because of his/her own socioeconomic characteristics or because of the treatment characteristics (e.g., price, risk). This underlines the importance of having a dialogue between practitioner and patient, and the need to discuss barriers to treatments (e.g., price, availability). The discrepancies identified in the LC model, particularly in class 6, could be explained by many factors and we highlighted two of them: (1) they chose the most to refuse one or the other treatment (7.30 over 11, ±2.91), which could lead to estimation issues, and (2) they reported higher CLBP according to the NPRS, higher frequency of use of painkillers, as well as a low satisfaction with life, which could induce a poor understanding about the choice‐based exercise although it was not reflected by the follow‐up questions (Appendix 11). Even so, we must be careful about such interpretations. Another limitation of this study is related to the choice of the software for analysis, which allows less flexibility in the methods used as compared to other software.

Finally, DCEs are in line with patient‐centred health care because they allow for consideration of patients' preferences. Socioeconomic background, illness experiences, the lifestyle of the patient and his/her preferences are now part of the care pathway. Knowing these preferences can be useful for decision‐makers to tailor care delivery to the needs of patients. Shared‐decision making improves patient adherence, involvement and satisfaction, and so, treatments' outcomes.^12^, ^46^ DCEs and, more generally, stated preference methods are a way to understand and measure patients' preferences and to ensure that they are well embedded in healthcare decision‐making.^47^ The study allowed us to determine the preferences of patients for CLBP nonsurgical treatments whereas only the surgery and corticosteroid injections were available in our healthcare institution at the time of the survey. These results may be used by managers and clinicians as a lever to better address patients' needs by offering them a wider variety of treatments through a reallocation of funds. They can also serve physicians and practitioners by improving information in monitoring patients for their needs and choices. In our local context, this led to nonsystematically offering corticosteroid injections and discussing other options with patients. It also led to a discussion for prioritization of resources to offer the preferred nonsurgical options assessed in this study and finally to a reorganization of the patient's care pathway.

## 5 CONCLUSION

We assessed the preferences of patients suffering from CLBP for six different nonsurgical treatments. We drew different patients' profiles and highlighted several differences between preferences and sociodemographic characteristics. As such, there is no one size fits all approach and we should consider the singularity of each patient, even if some common patterns can be found. For example, physical manipulations were highly preferred by most while corticosteroid injections were strongly rejected by a large minority, thus impacting the general results found in the HB model. Because of the heterogeneity and the complexity of behaviours towards choices for treatments, patients' preferences about treatments need a clearer understanding, especially for those facing CLBP which is a growing condition in developed countries with an ageing population. An array of different CLBP treatments exists but most of them are still understudied, unavailable or underused. We hope that it can help decision‐makers for organizing care access and delivery and at an individual level, to informing practitioners' sharing decisions with their patients for choosing what treatment best suits them for CLBP.

## CONFLICT OF INTEREST

The authors declare no conflict of interest.

## ETHICS STATEMENT

This study was approved by our university hospital ethics committee (CIUSSS de l'Estrie—CHUS) and all participants gave their informed consent before starting the survey. All responses were anonymous.

## Supporting information

Supporting information.

Supporting information.

Supporting information.

## Data Availability

Data will be made available upon reasonable request to the corresponding author.
