# Supplementary material for: Preferences of patients with chronic low back pain about nonsurgical treatments: Results of a discrete choice experiment
Source: Health Expect. 2022 Dec 8;26(1):510–30. doi: 10.1111/hex.13685 (PMC9854323; doi:10.1111/hex.13685)
Supplement: Supplementary file 1 — Supporting information. [file HEX-26--s001.docx]

Table of appendix

[Appendix 1 – Example of a choice card 1](#_Toc116729407)

[Appendix 2 – Conditional Logit model 2](#_Toc116729408)

[Appendix 3.1 – Latent class and clustering cross-tabulation between c = 4 and c = 6 latent classes clusters 4](#_Toc116729409)

[Appendix 3.2 – Hierarchical clustering on part-worth utilities derived from the Hierarchical Bayesian model 4](#_Toc116729410)

[Appendix 4 – Latent class models information criteria and log-likelihood functions 7](#_Toc116729411)

[Appendix 5 – Latent class assignment probabilities ($c=4$ and $c=6$) 8](#_Toc116729412)

Appendix 6 – Sociodemographic characteristics by latent classes ($c=4$) and full sample…………….9

Appendix 7 – Zero-centered utility values produced by the latent class analysis ($c=4$) and conditional relative importance of attributes………………………………………………………………………..[16](#_Toc116729413)

# Appendix 8 – Models’ results graphical representations……………………………………………….18

[Appendix 9 – Density of part worth utilities with one-sample multivariate tests of means ($H0:\mu=0$), Hierarchical Bayesian model ($n=424$) 24](#_Toc116729414)

[Appendix 10 – Respondent’s personal ranking of attributes and treatment modalities in total and per class from the latent class analysis ($c=4$ and $c=6$) (“What were the most important dimensions for you in choosing? Please rank the dimensions from 1 (the most important) to 7 (the least important).”; (“Please rank the 6 LBP treatments proposed from the most important (1) to the least important (6) in your opinion.”)) 28](#_Toc116729415)

[Appendix 11 – Discrete choice experiment perception (total and per group from the latent class analysis, $c=4$ and $c=6$) 33](#_Toc116729416)

[Appendix 12 – Sociodemographic characteristics, respondent’s personal ranking of attributes and treatment modalities, and discrete choice experiment perception by cluster derived from the Hierarchical Bayesian model ($c=4$ and $c=6$) and full sample 35](#_Toc116729417)

# **Appendix 1 – Example of a choice card**


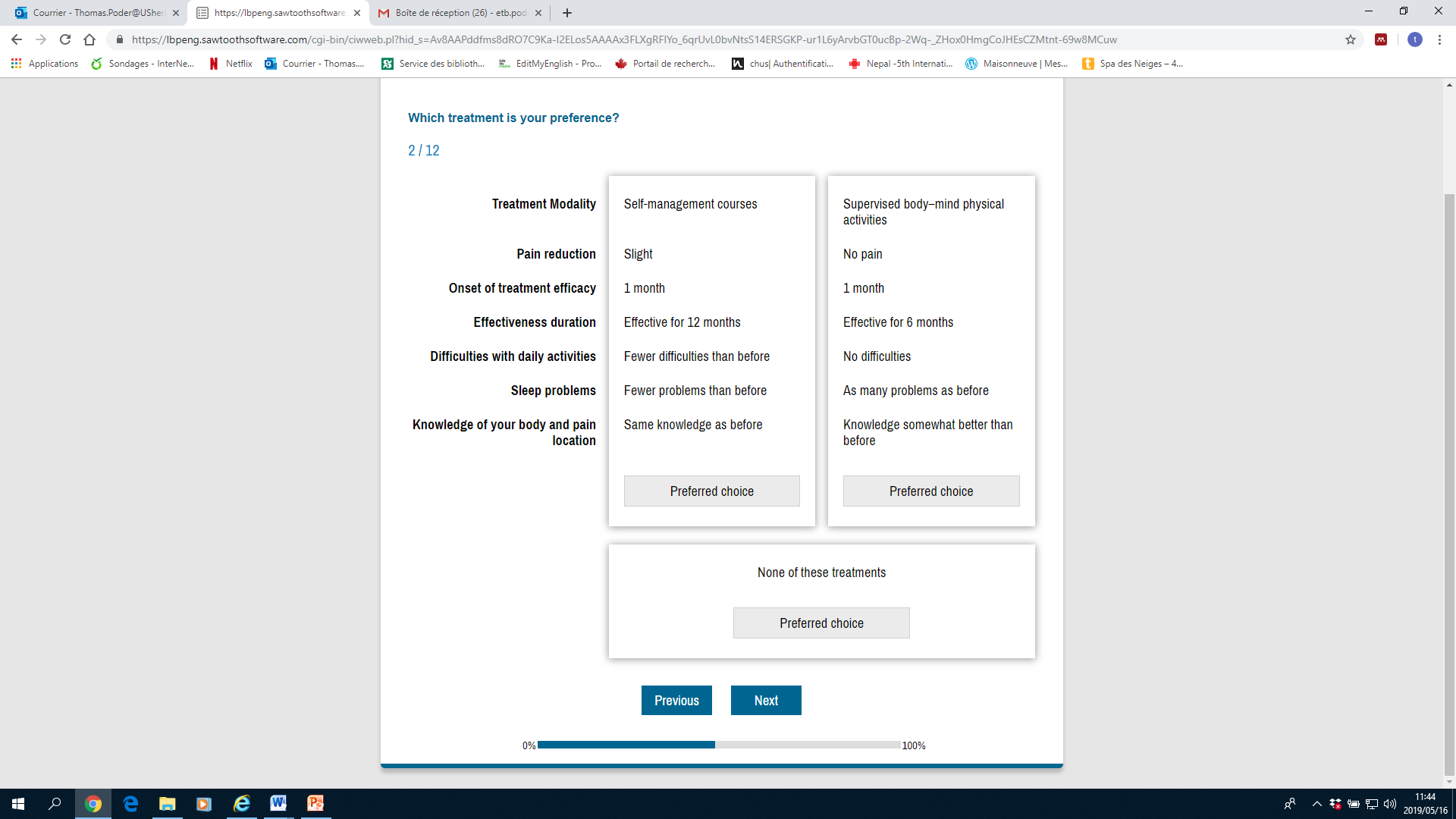


# **Appendix 2 – Conditional Logit model**

| **Conditional Logit model** | | | |
| --- | --- | --- | --- |
| **Attribute** | **Standardized utilities**^1^ | **Standard Error** | **t-ratio** |
|  |  |  |  |
| None^2^ | -110.63 | 8.21 | -13.47 |
|  |  |  |  |
| Treatment modality |  |  |  |
| Corticosteroid injections | -76.25 | 11.64 | -6.55 |
| Supervised body-mind physical activities | 35.61 | 11.16 | 3.19 |
| Supervised sports physical activities | 18.51 | 11.23 | 1.65 |
| Physical manipulations | 79.61 | 10.39 | 7.66 |
| Self-management courses | 27.50 | 11.04 | 2.49 |
| Psychotherapy | -84.99 | 12.03 | -7.07 |
| Pain reduction |  |  |  |
| None to very slight | -103.30 | 8.84 | -11.68 |
| Slight | -31.44 | 8.36 | -3.76 |
| Reduced by half | 49.44 | 7.91 | 6.25 |
| No pain | 85.30 | 8.45 | 10.09 |
| Onset of treatment efficacy |  |  |  |
| 1 month | 23.25 | 6.54 | 3.55 |
| 6 months | 7.89 | 6.77 | 1.17 |
| 12 months | -31.15 | 6.96 | -4.48 |
| Effectiveness duration |  |  |  |
| Effective for 2 months | -42.57 | 6.83 | -6.23 |
| Effective for 6 months | -1.53 | 6.60 | -0.23 |
| Effective for 12 months | 44.10 | 6.63 | 6.65 |
| Difficulties with daily activities |  |  |  |
| As many difficulties as before | -67.37 | 6.96 | -9.68 |
| Fewer difficulties as before | 27.18 | 6.77 | 4.01 |
| No difficulties | 40.19 | 6.47 | 6.21 |
| Sleep problems |  |  |  |
| As many problems as before | -42.03 | 6.68 | -6.29 |
| Fewer problems than before | 21.22 | 6.86 | 3.09 |
| No problems | 20.81 | 6.67 | 3.12 |
| Knowledge of his/her body and pain location | |  |  |
| Same knowledge as before | -16.94 | 6.69 | -2.53 |
| Knowledge somewhat better than before | -1.04 | 6.60 | -0.16 |
| Knowledge much better than before | 17.98 | 6.79 | 2.65 |
|  |  |  |  |
| Observations | 424 | | |
| McFadden R2 | 10.65 | | |
| Chi2 | 1,117.19 | | |
| Log likelihood | -4,685.08 | | |
| Log likelihood (null) | -5,243.68 | | |
| AIC | 9,408.16 | | |
| BIC | 9,531.10 | | |
|  |  |  |  |
| **Relative weight of attributes (%)** | | | |
| Treatment modality | 23.51 | | |
| Pain reduction | 26.94 | | |
| Onset of treatment efficacy | 7.77 | | |
| Effectiveness duration | 12.38 | | |
| Difficulties with daily activities | 15.37 | | |
| Sleep problems | 9.04 | | |
| Knowledge of his/her body and pain location | 4.99 | | |
|  |  |  |  |

Notes: AIC: Akaike Information Criterion; BIC: Bayesian Information Criterion.

^1^A value close to zero indicates an absence of preference: the further the value is from zero, the greater the preference.

^2^Coefficient of the opt-out option.

# **Appendix 3.1 – Latent class and clustering cross-tabulation between c = 4 and c = 6 latent classes clusters**

| **Number of latent classes** | **1** | **2** | **3** | **4** | **5** | **6** | **Total** |
| --- | --- | --- | --- | --- | --- | --- | --- |
| **1** | 1 | 25 | 5 | 63 | 0 | 0 | 94 |
| **2** | 43 | 0 | 27 | 0 | 30 | 0 | 100 |
| **3** | 1 | 4 | 6 | 3 | 0 | 50 | 64 |
| **4** | 3 | 28 | 5 | 1 | 129 | 0 | 166 |
| **Total** | 48 | 57 | 43 | 67 | 159 | 50 | 424 |

| **Number of clusters** | **1** | **2** | **3** | **4** | **5** | **6** | **Total** |
| --- | --- | --- | --- | --- | --- | --- | --- |
| **1** | 112 | 43 | 0 | 0 | 0 | 0 | 155 |
| **2** | 0 | 0 | 74 | 0 | 0 | 0 | 74 |
| **3** | 0 | 0 | 0 | 111 | 0 | 0 | 111 |
| **4** | 0 | 0 | 0 | 0 | 22 | 62 | 84 |
| **Total** | 112 | 43 | 74 | 111 | 22 | 62 | 424 |

Note: Ward's minimum variance method with L2 squared measure.

# **Appendix 3.2 – Hierarchical clustering on part-worth utilities derived from the Hierarchical Bayesian model**

| **Number of clusters** | **Calinski/Harabasz pseudo-F** | **Duda/Hart** | |
| --- | --- | --- | --- |
|  |  | **Je(2)/Je(1)** | **Pseudo T^2^** |
|  |  |  |  |
| 1 |  | 0.74 | 145.07 |
| 2 | 145.07 | 0.81 | 45.92 |
| 3 | 107.58 | 0.78 | 65.05 |
| 4 | 96.68 | 0.74 | 29.36 |
| 5 | 85.73 | 0.76 | 48.45 |
| 6 | 79.41 | 0.83 | 22.66 |
| 7 | 75.17 | 0.76 | 23.70 |
| 8 | 71.39 | 0.72 | 42.37 |
| 9 | 67.99 | 0.71 | 24.75 |
| 10 | 65.59 | 0.79 | 18.69 |
| 11 | 63.67 | 0.83 | 12.63 |
| 12 | 61.85 | 0.80 | 11.67 |
| 13 | 59.79 | 0.72 | 15.64 |
| 14 | 57.91 | 0.71 | 16.50 |
| 15 | 56.40 | 0.77 | 9.00 |
|  |  |  |  |

| **Attributes** | **Cluster 1** | | | | **Cluster 2** | | | | **Cluster 3** | | | | **Cluster 4** | | | |
| --- | --- | --- | --- | --- | --- | --- | --- | --- | --- | --- | --- | --- | --- | --- | --- | --- |
|  | **Observed Mean** | **Bootstrap Standard Error** | **Lower 95% CI** | **Upper 95% CI** | **Observed Mean** | **Bootstrap Standard Error** | **Lower 95% CI** | **Upper 95% CI** | **Observed Mean** | **Bootstrap Standard Error** | **Lower 95% CI** | **Upper 95% CI** | **Observed Mean** | **Bootstrap Standard Error** | **Lower 95% CI** | **Upper 95% CI** |
|  |  |  |  |  |  |  |  |  |  |  |  |  |  |  |  |  |
| None | -141.72 | 5.81 | -153.11 | -130.32 | -213.00 | 4.71 | -222.23 | -203.77 | 118.21 | 10.46 | 97.70 | 138.72 | 62.61 | 11.67 | 39.75 | 85.48 |
|  |  |  |  |  |  |  |  |  |  |  |  |  |  |  |  |  |
| Treatment modality |  |  |  |  |  |  |  |  |  |  |  |  |  |  |  |  |
| Corticosteroid injections | -138.68 | 5.05 | -148.59 | -128.78 | 25.43 | 10.12 | 5.59 | 45.28 | -96.43 | 7.02 | -110.19 | -82.67 | 97.52 | 10.85 | 76.25 | 118.79 |
| Supervised body-mind physical activities | 59.47 | 3.38 | 52.84 | 66.09 | -21.94 | 4.50 | -30.76 | -13.11 | 42.46 | 5.35 | 31.97 | 52.95 | -28.92 | 5.61 | -39.92 | -17.91 |
| Supervised sports physical activities | 17.45 | 6.50 | 4.71 | 30.19 | 24.91 | 10.14 | 5.03 | 44.80 | 11.09 | 9.51 | -7.55 | 29.73 | -37.75 | 9.17 | -55.73 | -19.78 |
| Physical manipulations | 46.88 | 2.98 | 41.04 | 52.71 | 30.02 | 4.81 | 20.60 | 39.43 | 85.69 | 4.84 | 76.21 | 95.17 | 39.20 | 5.84 | 27.75 | 50.65 |
| Self-management courses | 44.87 | 3.45 | 38.12 | 51.63 | -20.72 | 5.22 | -30.95 | -10.49 | 41.70 | 6.55 | 28.87 | 54.53 | -11.26 | 5.31 | -21.66 | -0.86 |
| Psychotherapy | -29.98 | 5.67 | -41.10 | -18.86 | -37.71 | 8.19 | -53.77 | -21.66 | -84.51 | 6.54 | -97.32 | -71.70 | -58.79 | 9.20 | -76.82 | -40.75 |
| Pain reduction |  |  |  |  |  |  |  |  |  |  |  |  |  |  |  |  |
| None to very slight | -79.49 | 2.15 | -83.70 | -75.28 | -73.56 | 4.42 | -82.23 | -64.90 | -47.09 | 3.65 | -54.24 | -39.93 | -35.09 | 5.15 | -45.18 | -25.00 |
| Slight | -20.87 | 1.54 | -23.88 | -17.85 | -0.55 | 3.06 | -6.54 | 5.44 | -26.96 | 2.27 | -31.40 | -22.51 | -9.61 | 3.11 | -15.70 | -3.52 |
| Reduced by half | 32.69 | 1.44 | 29.86 | 35.52 | 31.76 | 3.04 | 25.80 | 37.72 | 40.34 | 2.48 | 35.49 | 45.19 | 28.36 | 2.86 | 22.76 | 33.96 |
| No pain | 67.67 | 2.35 | 63.06 | 72.28 | 42.36 | 4.90 | 32.76 | 51.95 | 33.71 | 3.82 | 26.22 | 41.19 | 16.33 | 5.67 | 5.23 | 27.44 |
| Onset of treatment efficacy |  |  |  |  |  |  |  |  |  |  |  |  |  |  |  |  |
| 1 month | 9.39 | 1.70 | 6.05 | 12.73 | 6.75 | 4.14 | -1.37 | 14.88 | 22.18 | 2.92 | 16.46 | 27.89 | 12.90 | 3.94 | 5.19 | 20.62 |
| 6 months | 7.60 | 0.97 | 5.71 | 9.50 | 16.18 | 2.23 | 11.80 | 20.56 | 0.55 | 1.53 | -2.45 | 3.55 | 7.24 | 1.71 | 3.89 | 10.58 |
| 12 months | -17.00 | 1.80 | -20.52 | -13.47 | -22.93 | 3.33 | -29.46 | -16.40 | -22.73 | 2.85 | -28.32 | -17.13 | -20.14 | 3.84 | -27.67 | -12.60 |
| Duration effectiveness |  |  |  |  |  |  |  |  |  |  |  |  |  |  |  |  |
| Effective for 2 months | -33.10 | 1.49 | -36.02 | -30.18 | -16.67 | 3.28 | -23.10 | -10.25 | -34.19 | 2.55 | -39.18 | -29.20 | -13.35 | 3.93 | -21.06 | -5.65 |
| Effective for 6 months | 1.80 | 1.56 | -1.25 | 4.86 | -12.49 | 2.79 | -17.96 | -7.03 | 15.89 | 2.33 | 11.33 | 20.45 | -4.07 | 3.22 | -10.38 | 2.24 |
| Effective for 12 months | 31.30 | 1.46 | 28.43 | 34.16 | 29.17 | 3.30 | 22.71 | 35.62 | 18.30 | 1.88 | 14.62 | 21.98 | 17.42 | 3.13 | 11.29 | 23.56 |
| Difficulties with daily activities |  |  |  |  |  |  |  |  |  |  |  |  |  |  |  |  |
| As many difficulties as before | -44.76 | 1.77 | -48.23 | -41.29 | -65.00 | 3.09 | -71.06 | -58.94 | -28.15 | 2.54 | -33.13 | -23.16 | -38.16 | 3.63 | -45.27 | -31.05 |
| Fewer difficulties as before | 20.21 | 1.12 | 18.01 | 22.41 | 18.72 | 2.45 | 13.91 | 23.53 | 15.38 | 1.87 | 11.71 | 19.06 | 13.23 | 2.66 | 8.02 | 18.43 |
| No difficulties | 24.55 | 1.64 | 21.34 | 27.77 | 46.28 | 2.15 | 42.06 | 50.50 | 12.76 | 2.13 | 8.58 | 16.95 | 24.93 | 2.41 | 20.20 | 29.66 |
| Sleep problems |  |  |  |  |  |  |  |  |  |  |  |  |  |  |  |  |
| As many problems as before | -34.02 | 1.89 | -37.72 | -30.33 | -24.91 | 3.71 | -32.19 | -17.63 | -24.33 | 3.17 | -30.54 | -18.11 | -10.57 | 4.25 | -18.90 | -2.24 |
| Fewer problems than before | 13.90 | 1.29 | 11.38 | 16.42 | 11.48 | 2.43 | 6.71 | 16.25 | 13.45 | 1.85 | 9.84 | 17.07 | 19.42 | 2.26 | 14.98 | 23.85 |
| No problems | 20.13 | 1.74 | 16.72 | 23.53 | 13.43 | 3.41 | 6.75 | 20.11 | 10.87 | 2.89 | 5.21 | 16.54 | -8.85 | 4.07 | -16.82 | -0.88 |
| Knowledge of his/her body and pain location |  |  |  |  |  |  |  |  |  |  |  |  |  |  |  |  |
| Same knowledge as before | -16.70 | 1.00 | -18.66 | -14.74 | -7.48 | 2.59 | -12.56 | -2.40 | -6.90 | 1.66 | -10.15 | -3.65 | 2.41 | 2.72 | -2.92 | 7.74 |
| Knowledge somewhat better than before | -0.78 | 1.10 | -2.93 | 1.37 | -0.45 | 2.27 | -4.91 | 4.00 | -4.10 | 1.65 | -7.34 | -0.87 | -2.11 | 2.32 | -6.66 | 2.44 |
| Knowledge much better than before | 17.48 | 1.05 | 15.42 | 19.54 | 7.93 | 2.49 | 3.05 | 12.82 | 11.00 | 1.73 | 7.62 | 14.39 | -0.31 | 2.47 | -5.14 | 4.53 |
|  |  |  |  |  |  |  |  |  |  |  |  |  |  |  |  |  |
| Observations | 424 |  |  |  |  |  |  |  |  |  |  |  |  |  |  |  |
| Method | Ward's minimum variance method | | | |  |  |  |  |  |  |  |  |  |  |  |  |
| Measure | L2 squared | |  |  |  |  |  |  |  |  |  |  |  |  |  |  |
| Calinski-Harabasz pseudo-F index | 96.68 |  |  |  |  |  |  |  |  |  |  |  |  |  |  |  |
| Duda–Hart Je(2)/Je(1) index | 0.74 |  |  |  |  |  |  |  |  |  |  |  |  |  |  |  |
| Duda–Hart pseudo T^2^ | 29.36 |  |  |  |  |  |  |  |  |  |  |  |  |  |  |  |
|  |  |  |  |  |  |  |  |  |  |  |  |  |  |  |  |  |

Note: Standard error and 95% confidence interval were obtained by bootstrapping method with $10,000$ replications.

| **Hierarchical clustering on part-worth utilities derived from the Hierarchical Bayesian model** | | | | | | | | | | | | | | | | | | | | | | | | |
| --- | --- | --- | --- | --- | --- | --- | --- | --- | --- | --- | --- | --- | --- | --- | --- | --- | --- | --- | --- | --- | --- | --- | --- | --- |
| **Attributes** | **Cluster 1** | | | | **Cluster 2** | | | | **Cluster 3** | | | | **Cluster 4** | | | | **Cluster 5** | | | | **Cluster 6** | | | |
|  | **Observed Mean** | **Bootstrap Standard Error** | **Lower 95% CI** | **Upper 95% CI** | **Observed Mean** | **Bootstrap Standard Error** | **Lower 95% CI** | **Upper 95% CI** | **Observed Mean** | **Bootstrap Standard Error** | **Lower 95% CI** | **Upper 95% CI** | **Observed Mean** | **Bootstrap Standard Error** | **Lower 95% CI** | **Upper 95% CI** | **Observed Mean** | **Bootstrap Standard Error** | **Lower 95% CI** | **Upper 95% CI** | **Observed Mean** | **Bootstrap Standard Error** | **Lower 95% CI** | **Upper 95% CI** |
|  |  |  |  |  |  |  |  |  |  |  |  |  |  |  |  |  |  |  |  |  |  |  |  |  |
|  |  |  |  |  |  |  |  |  |  |  |  |  |  |  |  |  |  |  |  |  |  |  |  |  |
| None | -154.09 | 5.29 | -164.46 | -143.72 | -109.49 | 15.02 | -138.93 | -80.05 | -213.00 | 4.71 | -222.23 | -203.77 | 118.21 | 10.46 | 97.70 | 138.72 | 200.60 | 20.15 | 161.10 | 240.09 | 13.65 | 7.38 | -0.82 | 28.12 |
|  |  |  |  |  |  |  |  |  |  |  |  |  |  |  |  |  |  |  |  |  |  |  |  |  |
| Treatment modality |  |  |  |  |  |  |  |  |  |  |  |  |  |  |  |  |  |  |  |  |  |  |  |  |
| Corticosteroid injections | -116.22 | 5.11 | -126.23 | -106.20 | -197.20 | 6.40 | -209.75 | -184.65 | 25.43 | 10.12 | 5.59 | 45.28 | -96.43 | 7.02 | -110.19 | -82.67 | 214.30 | 14.71 | 185.46 | 243.13 | 56.08 | 9.05 | 38.34 | 73.82 |
| Supervised body-mind physical activities | 43.40 | 2.87 | 37.76 | 49.03 | 101.32 | 6.00 | 89.56 | 113.07 | -21.94 | 4.50 | -30.76 | -13.11 | 42.46 | 5.35 | 31.97 | 52.95 | -72.41 | 6.59 | -85.33 | -59.49 | -13.49 | 6.13 | -25.51 | -1.47 |
| Supervised sports physical activities | -5.50 | 6.98 | -19.17 | 8.18 | 77.22 | 9.96 | 57.69 | 96.74 | 24.91 | 10.14 | 5.03 | 44.80 | 11.09 | 9.51 | -7.55 | 29.73 | -60.25 | 15.33 | -90.30 | -30.21 | -29.77 | 10.98 | -51.30 | -8.24 |
| Physical manipulations | 43.57 | 3.40 | 36.90 | 50.24 | 55.48 | 5.90 | 43.92 | 67.05 | 30.02 | 4.81 | 20.60 | 39.43 | 85.69 | 4.84 | 76.21 | 95.17 | 30.71 | 6.57 | 17.84 | 43.58 | 42.22 | 7.58 | 27.36 | 57.07 |
| Self-management courses | 33.43 | 3.57 | 26.43 | 40.42 | 74.69 | 6.36 | 62.22 | 87.17 | -20.72 | 5.22 | -30.95 | -10.49 | 41.70 | 6.55 | 28.87 | 54.53 | -26.69 | 7.44 | -41.27 | -12.12 | -5.78 | 6.55 | -18.63 | 7.06 |
| Psychotherapy | 1.32 | 4.75 | -7.99 | 10.63 | -111.51 | 7.30 | -125.83 | -97.19 | -37.71 | 8.19 | -53.77 | -21.66 | -84.51 | 6.54 | -97.32 | -71.70 | -85.65 | 12.23 | -109.62 | -61.67 | -49.25 | 11.51 | -71.82 | -26.69 |
| Pain reduction |  |  |  |  |  |  |  |  |  |  |  |  |  |  |  |  |  |  |  |  |  |  |  |  |
| None to very slight | -89.11 | 1.82 | -92.67 | -85.55 | -54.45 | 4.29 | -62.85 | -46.05 | -73.56 | 4.42 | -82.23 | -64.90 | -47.09 | 3.65 | -54.24 | -39.93 | 8.49 | 9.00 | -9.15 | 26.13 | -50.55 | 4.90 | -60.16 | -40.94 |
| Slight | -23.37 | 1.64 | -26.60 | -20.15 | -14.34 | 3.36 | -20.93 | -7.75 | -0.55 | 3.06 | -6.54 | 5.44 | -26.96 | 2.27 | -31.40 | -22.51 | 7.52 | 4.39 | -1.09 | 16.13 | -15.69 | 3.64 | -22.83 | -8.55 |
| Reduced by half | 37.63 | 1.47 | 34.75 | 40.51 | 19.82 | 2.69 | 14.55 | 25.10 | 31.76 | 3.04 | 25.80 | 37.72 | 40.34 | 2.48 | 35.49 | 45.19 | 18.25 | 5.13 | 8.19 | 28.31 | 31.95 | 3.30 | 25.48 | 38.41 |
| No pain | 74.85 | 2.14 | 70.67 | 79.04 | 48.96 | 5.57 | 38.04 | 59.89 | 42.36 | 4.90 | 32.76 | 51.95 | 33.71 | 3.82 | 26.22 | 41.19 | -34.26 | 7.76 | -49.46 | -19.06 | 34.29 | 5.71 | 23.09 | 45.49 |
| Onset of treatment efficacy |  |  |  |  |  |  |  |  |  |  |  |  |  |  |  |  |  |  |  |  |  |  |  |  |
| 1 month | 15.14 | 1.82 | 11.57 | 18.71 | -5.58 | 2.78 | -11.03 | -0.14 | 6.75 | 4.14 | -1.37 | 14.88 | 22.18 | 2.92 | 16.46 | 27.89 | -0.18 | 4.99 | -9.97 | 9.60 | 17.55 | 4.90 | 7.94 | 27.16 |
| 6 months | 7.42 | 1.11 | 5.24 | 9.60 | 8.09 | 1.98 | 4.20 | 11.98 | 16.18 | 2.23 | 11.80 | 20.56 | 0.55 | 1.53 | -2.45 | 3.55 | 9.49 | 2.63 | 4.33 | 14.65 | 6.44 | 2.12 | 2.27 | 10.60 |
| 12 months | -22.56 | 2.02 | -26.51 | -18.61 | -2.51 | 2.79 | -7.97 | 2.96 | -22.93 | 3.33 | -29.46 | -16.40 | -22.73 | 2.85 | -28.32 | -17.13 | -9.30 | 5.03 | -19.16 | 0.55 | -23.98 | 4.81 | -33.42 | -14.55 |
| Duration effectiveness |  |  |  |  |  |  |  |  |  |  |  |  |  |  |  |  |  |  |  |  |  |  |  |  |
| Effective for 2 months | -37.89 | 1.61 | -41.05 | -34.73 | -20.63 | 2.50 | -25.52 | -15.74 | -16.67 | 3.28 | -23.10 | -10.25 | -34.19 | 2.55 | -39.18 | -29.20 | 6.77 | 3.78 | -0.64 | 14.18 | -20.49 | 4.86 | -30.01 | -10.98 |
| Effective for 6 months | 6.10 | 1.71 | 2.75 | 9.45 | -9.38 | 2.75 | -14.77 | -4.00 | -12.49 | 2.79 | -17.96 | -7.03 | 15.89 | 2.33 | 11.33 | 20.45 | -9.41 | 4.41 | -18.06 | -0.76 | -2.18 | 4.04 | -10.09 | 5.74 |
| Effective for 12 months | 31.79 | 1.69 | 28.47 | 35.10 | 30.02 | 2.92 | 24.30 | 35.73 | 29.17 | 3.30 | 22.71 | 35.62 | 18.30 | 1.88 | 14.62 | 21.98 | 2.64 | 3.79 | -4.79 | 10.08 | 22.67 | 3.85 | 15.11 | 30.22 |
| Difficulties with daily activities |  |  |  |  |  |  |  |  |  |  |  |  |  |  |  |  |  |  |  |  |  |  |  |  |
| As many difficulties as before | -52.21 | 1.83 | -55.79 | -48.63 | -25.36 | 2.55 | -30.35 | -20.37 | -65.00 | 3.09 | -71.06 | -58.94 | -28.15 | 2.54 | -33.13 | -23.16 | -10.27 | 6.17 | -22.37 | 1.83 | -48.06 | 3.68 | -55.27 | -40.84 |
| Fewer difficulties as before | 21.31 | 1.34 | 18.68 | 23.93 | 17.35 | 2.01 | 13.40 | 21.29 | 18.72 | 2.45 | 13.91 | 23.53 | 15.38 | 1.87 | 11.71 | 19.06 | -4.27 | 3.70 | -11.53 | 2.99 | 19.44 | 3.02 | 13.53 | 25.35 |
| No difficulties | 30.90 | 1.61 | 27.74 | 34.07 | 8.01 | 2.94 | 2.26 | 13.77 | 46.28 | 2.15 | 42.06 | 50.50 | 12.76 | 2.13 | 8.58 | 16.95 | 14.54 | 4.34 | 6.04 | 23.04 | 28.62 | 2.76 | 23.20 | 34.03 |
| Sleep problems |  |  |  |  |  |  |  |  |  |  |  |  |  |  |  |  |  |  |  |  |  |  |  |  |
| As many problems as before | -41.16 | 1.48 | -44.06 | -38.26 | -15.43 | 4.55 | -24.35 | -6.51 | -24.91 | 3.71 | -32.19 | -17.63 | -24.33 | 3.17 | -30.54 | -18.11 | 24.07 | 6.87 | 10.61 | 37.53 | -22.86 | 4.25 | -31.18 | -14.54 |
| Fewer problems than before | 16.09 | 1.44 | 13.27 | 18.91 | 8.19 | 2.55 | 3.19 | 13.19 | 11.48 | 2.43 | 6.71 | 16.25 | 13.45 | 1.85 | 9.84 | 17.07 | 10.96 | 4.10 | 2.92 | 19.01 | 22.42 | 2.61 | 17.30 | 27.54 |
| No problems | 25.07 | 1.42 | 22.28 | 27.86 | 7.24 | 4.47 | -1.53 | 16.01 | 13.43 | 3.41 | 6.75 | 20.11 | 10.87 | 2.89 | 5.21 | 16.54 | -35.04 | 5.33 | -45.48 | -24.59 | 0.44 | 4.64 | -8.65 | 9.54 |
| Knowledge of his/her body and pain location |  |  |  |  |  |  |  |  |  |  |  |  |  |  |  |  |  |  |  |  |  |  |  |  |
| Same knowledge as before | -17.17 | 1.15 | -19.42 | -14.93 | -15.47 | 2.02 | -19.44 | -11.50 | -7.48 | 2.59 | -12.56 | -2.40 | -6.90 | 1.66 | -10.15 | -3.65 | 25.92 | 3.81 | 18.45 | 33.38 | -5.93 | 2.75 | -11.33 | -0.53 |
| Knowledge somewhat better than before | 0.37 | 1.22 | -2.03 | 2.77 | -3.77 | 2.27 | -8.21 | 0.68 | -0.45 | 2.27 | -4.91 | 4.00 | -4.10 | 1.65 | -7.34 | -0.87 | -6.74 | 3.13 | -12.87 | -0.61 | -0.46 | 2.95 | -6.25 | 5.33 |
| Knowledge much better than before | 16.80 | 1.12 | 14.61 | 19.00 | 19.24 | 2.47 | 14.40 | 24.07 | 7.93 | 2.49 | 3.05 | 12.82 | 11.00 | 1.73 | 7.62 | 14.39 | -19.18 | 2.94 | -24.94 | -13.41 | 6.39 | 2.70 | 1.10 | 11.68 |
|  |  |  |  |  |  |  |  |  |  |  |  |  |  |  |  |  |  |  |  |  |  |  |  |  |
| Observations | 424 |  |  |  |  |  |  |  |  |  |  |  |  |  |  |  |  |  |  |  |  |  |  |  |
| Method | Ward's minimum variance method | | |  |  |  |  |  |  |  |  |  |  |  |  |  |  |  |  |  |  |  |  |  |
| Dissimilarity measure | L2 squared |  |  |  |  |  |  |  |  |  |  |  |  |  |  |  |  |  |  |  |  |  |  |  |
| Calinski-Harabasz pseudo-F index | 79.41 |  |  |  |  |  |  |  |  |  |  |  |  |  |  |  |  |  |  |  |  |  |  |  |
| Duda–Hart Je(2)/Je(1) index | 0.83 |  |  |  |  |  |  |  |  |  |  |  |  |  |  |  |  |  |  |  |  |  |  |  |
| Duda–Hart pseudo T^2^ | 22.66 |  |  |  |  |  |  |  |  |  |  |  |  |  |  |  |  |  |  |  |  |  |  |  |
|  |  |  |  |  |  |  |  |  |  |  |  |  |  |  |  |  |  |  |  |  |  |  |  |  |

Note: Standard error and 95% confidence interval were obtained by bootstrapping method with $10,000$ replications.

# **Appendix 4 – Latent class models information criteria and log-likelihood functions**

| **Number of latent classes** | **AIC** | **CAIC** | **BIC** | **ABIC** | **LLF** |
| --- | --- | --- | --- | --- | --- |
|  |  |  |  |  |  |
| 2 | 8,451.02 | 8,742.38 | 8,703.38 | 8,579.45 | -4,186.51 |
| 3 | 8,285.83 | 8,726.60 | 8,667.60 | 8,480.12 | -4,082.18 |
| 4 | 8,143.55 | 8,733.74 | 8,654.74 | 8,403.71 | -3,990.45 |
| 5 | 8,070.85 | 8,810.45 | 8,711.45 | 8,396.86 | -3,924.90 |
| 6 | 7,994.88 | 8,883.89 | 8,764.89 | 8,386.76 | -3,876.09 |
|  |  |  |  |  |  |

Notes: AIC: Akaike Information Criterion; CAIC: Consistent Akaike Information Criterion; BIC: Bayesian Information Criterion; ABIC: Adjusted Bayesian Information Criterion; LLF: Log-Likelihood Function.

# **Appendix 5 – Latent class assignment probabilities (**$\boldsymbol{c=4}$ **and** $\boldsymbol{c=6}$**)**

| **Latent class assignment probabilities** | **Latent Class Logit** | | | |
| --- | --- | --- | --- | --- |
|  | Class 1 | Class 2 | Class 3 | Class 4 |
|  |  |  |  |  |
| Mean | 0.230 | 0.253 | 0.150 | 0.367 |
| Standard deviation | 0.372 | 0.374 | 0.328 | 0.424 |
| Range | (0.000-1.000) | (0.000-1.000) | (0.000-1.000) | (0.000-1.000) |
| Median | 0.003 | 0.020 | 0.000 | 0.056 |
| Skewness | 1.318 | 1.185 | 1.948 | 0.514 |
| Kurtosis | 2.984 | 2.665 | 4.991 | 1.440 |
|  |  |  |  |  |

| **Latent class assignment probabilities** | **Latent Class Logit** | | | | | |
| --- | --- | --- | --- | --- | --- | --- |
|  | Class 1 | Class 2 | Class 3 | Class 4 | Class 5 | Class 6 |
|  |  |  |  |  |  |  |
| Mean | 0.126 | 0.141 | 0.106 | 0.157 | 0.349 | 0.120 |
| Standard deviation | 0.279 | 0.301 | 0.266 | 0.327 | 0.427 | 0.302 |
| Range | (0.000-1.000) | (0.000-1.000) | (0.000-1.000) | (0.000-1.000) | (0.000-0.999) | (0.000-1.000) |
| Median | 0.003 | 0.002 | 0.000 | 0.000 | 0.030 | 0.000 |
| Skewness | 2.344 | 2.090 | 2.613 | 1.884 | 0.600 | 2.366 |
| Kurtosis | 6.970 | 5.741 | 8.331 | 4.787 | 1.512 | 6.782 |
|  |  |  |  |  |  |  |

Note: the latent class logit model does not assign individuals to a class, hence the term “latent”. However, it generates class membership probabilities for each individual. To assign individual to each latent class, we considered the maximum class membership probability.

**Appendix 6 – Sociodemographic characteristics by latent classes (**$\boldsymbol{c=4}$**) and full sample**

| **Sociodemographic characteristics** | **Class 1** | **Class 2** | **Class 3** | **Class 4** | **Total** | **P-value**^1^ |
| --- | --- | --- | --- | --- | --- | --- |
|  |  |  |  |  |  |  |
| **Observations** |  |  |  |  |  |  |
| n | 94 | 100 | 64 | 166 | 424 | - |
| Class share | 22.17% | 23.58% | 15.09% | 39.15% | 100.00% |  |
| **Gender** |  |  |  |  |  |  |
| Male | 19.15% | 20.00% | 32.81% | 13.86% | 19.34% | **0.003** |
| Female | 80.85% | 80.00% | 64.06% | 86.14% | 80.19% |  |
| Intersex | 0.00% | 0.00% | 3.13% | 0.00% | 0.47% |  |
|  |  |  |  |  |  |  |
| Female/Male Ratio | 4.22 | 4.00 | 1.95 | 6.22 | 4.15 | - |
| **Age (years)** |  |  |  |  |  |  |
| Mean | 55.95 | 54.97 | 61.56 | 52.91 | 55.38 | **<.001** |
| Standard Deviation | 13.22 | 12.50 | 10.35 | 12.39 | 12.62 |  |
| Range | (25-85) | (27-83) | (32-82) | (20-87) | (20-87) |  |
|  |  |  |  |  |  |  |
| Less than 35 | 5.32% | 5.00% | 1.56% | 6.02% | 4.95% | **0.027** |
| 35-39 | 5.32% | 8.00% | 0.00% | 7.23% | 5.90% |  |
| 40-44 | 11.70% | 11.00% | 4.69% | 16.87% | 12.50% |  |
| 45-49 | 5.32% | 8.00% | 7.81% | 9.04% | 7.78% |  |
| 50-54 | 18.09% | 13.00% | 9.38% | 13.86% | 13.92% |  |
| 55-59 | 12.77% | 14.00% | 15.63% | 16.87% | 15.09% |  |
| 60-64 | 8.51% | 16.00% | 17.19% | 12.05% | 12.97% |  |
| 65-69 | 14.89% | 14.00% | 15.63% | 11.45% | 13.44% |  |
| 70-74 | 12.77% | 5.00% | 18.75% | 3.01% | 8.02% |  |
| 75 or more | 5.32% | 6.00% | 9.38% | 3.61% | 5.42% |  |
| **Body Mass Index (BMI)** |  |  |  |  |  |  |
| Mean | 29.68 | 29.18 | 29.29 | 29.11 | 29.28 | 0.927 |
| Standard Deviation | 6.91 | 7.05 | 5.87 | 6.46 | 6.60 |  |
| Range | (19.96-58.37) | (18.42-62.75) | (19.07-49.94) | (13.71-50.81) | (13.71-62.75) |  |
| **Marital status** |  |  |  |  |  |  |
| Married | 40.43% | 40.00% | 35.94% | 31.33% | 36.08% | 0.529 |
| Living with a partner | 19.15% | 28.00% | 18.75% | 25.90% | 23.82% |  |
| Single | 18.09% | 13.00% | 12.50% | 20.48% | 16.98% |  |
| Separated | 5.32% | 5.00% | 6.25% | 7.83% | 6.37% |  |
| Divorced | 12.77% | 10.00% | 21.88% | 12.05% | 13.21% |  |
| Widowed | 4.26% | 4.00% | 4.69% | 2.41% | 3.54% |  |
| **Occupational status** |  |  |  |  |  |  |
| Employed | 20.21% | 21.00% | 6.25% | 24.70% | 20.05% | **0.040** |
| Self-employed | 4.26% | 3.00% | 9.38% | 6.63% | 5.66% |  |
| Retired | 42.55% | 35.00% | 48.44% | 29.52% | 36.56% |  |
| At home | 3.19% | 6.00% | 7.81% | 4.22% | 4.95% |  |
| Student | 2.13% | 0.00% | 0.00% | 4.82% | 2.36% |  |
| Unemployed | 4.26% | 3.00% | 3.13% | 1.81% | 2.83% |  |
| Sick leave | 9.57% | 17.00% | 14.06% | 12.65% | 13.21% |  |
| Parental leave | 1.06% | 0.00% | 0.00% | 0.00% | 0.24% |  |
| Other (e.g., disability) | 12.77% | 15.00% | 10.94% | 15.66% | 14.15% |  |
| **Educational level** |  |  |  |  |  |  |
| Secondary or less and Diploma of professional studies | 28.72% | 27.00% | 37.50% | 22.29% | 27.12% | **0.048** |
| College and CEGEP | 27.66% | 29.00% | 35.94% | 25.30% | 28.30% |  |
| Baccalaureate, Master and PhD | 43.62% | 42.00% | 25.00% | 51.20% | 43.40% |  |
| Other | 0.00% | 2.00% | 1.56% | 1.20% | 1.18% |  |
| **Annual household income (CAD)** |  |  |  |  |  |  |
| Mean | 59,707.45 | 63,250.00 | 51,210.94 | 56,189.76 | 57,883.25 | 0.203 |
| Standard Deviation | 37,288.57 | 37,961.80 | 34,577.93 | 38,517.71 | 37,618.81 |  |
| Range | (12,500-165,000) | (7,500-165,000) | (7,500-165,000) | (2,500-165,000) | (2,500-165,000) |  |
| **Living with an adult** |  |  |  |  |  |  |
| Yes | 69.15% | 69.00% | 59.38% | 65.06% | 66.04% | 0.545 |
| No | 30.85% | 31.00% | 40.63% | 34.94% | 33.96% |  |
| **Under-age dependent children (at least one)** |  |  |  |  |  |  |
| Yes | 12.77% | 17.00% | 10.94% | 18.67% | 15.80% | 0.403 |
| No | 87.23% | 83.00% | 89.06% | 81.33% | 84.20% |  |
| **Type of residence** |  |  |  |  |  |  |
| Rural | 31.91% | 23.00% | 32.81% | 29.52% | 29.01% | 0.456 |
| Urban | 68.09% | 77.00% | 67.19% | 70.48% | 70.99% |  |
| **Owning a home** |  |  |  |  |  |  |
| Yes | 65.96% | 64.00% | 59.38% | 66.27% | 64.62% | 0.788 |
| No | 34.04% | 36.00% | 40.63% | 33.73% | 35.38% |  |
| **Smoking** |  |  |  |  |  |  |
| Yes | 10.64% | 12.00% | 15.63% | 14.46% | 13.21% | 0.748 |
| No | 89.36% | 88.00% | 84.38% | 85.54% | 86.79% |  |
| **Diagnosis given by a medical doctor** |  |  |  |  |  |  |
| No diagnosis | 3.19% | 0.00% | 7.81% | 6.02% | 4.25% | **0.018** |
| Muscle and/or ligament sprain | 9.57% | 8.00% | 12.50% | 8.43% | 9.20% | 0.768 |
| Sciatica | 28.72% | 24.00% | 31.25% | 24.70% | 26.42% | 0.664 |
| Lumbar disc herniation | 37.23% | 36.00% | 37.50% | 39.16% | 37.74% | 0.963 |
| Degenerative disc disease | 15.96% | 16.00% | 14.06% | 20.48% | 17.45% | 0.602 |
| Facet arthritis | 34.04% | 44.00% | 34.38% | 30.12% | 34.91% | 0.148 |
| Vertebral arthritis or spondylarthrosis | 15.96% | 14.00% | 25.00% | 9.04% | 14.15% | **0.018** |
| Spondylolisthesis | 5.32% | 7.00% | 6.25% | 6.63% | 6.37% | 0.983 |
| Deformation (e.g., scoliosis, kyphosis) | 10.64% | 10.00% | 7.81% | 11.45% | 10.38% | 0.878 |
| Osteoporosis | 8.51% | 3.00% | 7.81% | 9.04% | 7.31% | 0.254 |
| Osteoporosis with spinal fracture | 1.06% | 1.00% | 3.13% | 0.00% | 0.94% | **0.081** |
| Fracture or dislocation of the spine | 2.13% | 1.00% | 6.25% | 1.20% | 2.12% | 0.102 |
| Autoimmune inflammatory disease | 3.19% | 8.00% | 1.56% | 3.61% | 4.25% | 0.237 |
| Fibromyalgia | 34.04% | 38.00% | 31.25% | 35.54% | 35.14% | 0.839 |
| Other diagnosis | 23.40% | 25.00% | 26.56% | 25.90% | 25.24% | 0.966 |
| **How long are you suffering from low back pain?** |  |  |  |  |  |  |
| Between 3 months and 1 year | 0.00% | 3.00% | 0.00% | 3.01% | 1.89% | 0.184 |
| More than 1 year | 100.00% | 97.00% | 100.00% | 96.99% | 98.11% |  |
| **Today low back pain** |  |  |  |  |  |  |
| Mean | 5.15 | 5.72 | 5.86 | 5.15 | 5.39 | **0.021** |
| Standard Deviation | 2.07 | 1.88 | 2.20 | 1.99 | 2.03 |  |
| Range | (0-10) | (2-10) | (0-10) | (0-10) | (0-10) |  |
| **Worst level of low back pain in the past two weeks** |  |  |  |  |  |  |
| Mean | 7.40 | 7.72 | 7.59 | 7.47 | 7.53 | 0.631 |
| Standard Deviation | 2.15 | 1.61 | 2.06 | 1.73 | 1.85 |  |
| Range | (0-10) | (4-10) | (2-10) | (2-10) | (0-10) |  |
| **Average level of low back pain in the past two weeks** |  |  |  |  |  |  |
| Mean | 5.36 | 5.78 | 5.97 | 5.31 | 5.53 | **0.041** |
| Standard Deviation | 2.00 | 1.71 | 2.15 | 1.76 | 1.88 |  |
| Range | (0-10) | (2-10) | (1-10) | (1-10) | (0-10) |  |
| **Frequency of use of painkillers** |  |  |  |  |  |  |
| Several times a day | 23.40% | 34.00% | 43.75% | 25.30% | 29.72% | 0.409 |
| Every day | 34.04% | 29.00% | 28.13% | 31.93% | 31.13% |  |
| Several times a week | 12.77% | 18.00% | 9.38% | 15.06% | 14.39% |  |
| Once a week | 3.19% | 3.00% | 1.56% | 1.81% | 2.36% |  |
| Several times a month | 6.38% | 7.00% | 1.56% | 9.04% | 6.84% |  |
| Once a month | 2.13% | 3.00% | 1.56% | 4.22% | 3.07% |  |
| Several times a year | 8.51% | 3.00% | 7.81% | 6.02% | 6.13% |  |
| Once a year | 1.06% | 1.00% | 0.00% | 0.60% | 0.71% |  |
| Never | 8.51% | 2.00% | 6.25% | 6.02% | 5.66% |  |
| **Treatments for reducing pain other than painkillers** |  |  |  |  |  |  |
| Homeopathic products | 4.26% | 3.00% | 7.81% | 11.45% | 7.31% | **0.044** |
| Infiltration of corticosteroid products | 31.91% | 56.00% | 42.19% | 24.10% | 36.08% | **<.001** |
| Chiropractic sessions | 21.28% | 12.00% | 9.38% | 12.65% | 13.92% | 0.118 |
| Physiotherapy sessions | 28.72% | 38.00% | 23.44% | 39.16% | 34.20% | 0.072 |
| Osteopathy sessions | 28.72% | 21.00% | 20.31% | 26.51% | 24.76% | 0.475 |
| Occupational therapy sessions | 2.13% | 7.00% | 12.50% | 5.42% | 6.13% | **0.064** |
| Psychotherapy sessions | 5.32% | 17.00% | 7.81% | 12.65% | 11.32% | **0.054** |
| Reflexology sessions | 3.19% | 1.00% | 4.69% | 3.61% | 3.07% | 0.503 |
| Massage therapy sessions | 45.74% | 48.00% | 31.25% | 40.36% | 41.98% | 0.155 |
| Yoga sessions | 12.77% | 8.00% | 10.94% | 24.10% | 15.80% | **0.002** |
| Stretching sessions | 11.70% | 15.00% | 17.19% | 24.70% | 18.40% | **0.045** |
| Acupuncture sessions | 9.57% | 14.00% | 17.19% | 13.86% | 13.44% | 0.566 |
| Cupping sessions | 3.19% | 3.00% | 0.00% | 3.01% | 2.59% | 0.580 |
| Infrared frequency sessions | 1.06% | 3.00% | 0.00% | 1.81% | 1.65% | 0.595 |
| Bodybuilding | 7.45% | 7.00% | 14.06% | 13.86% | 10.85% | 0.180 |
| Endurance activities (aerobic) | 5.32% | 12.00% | 4.69% | 10.24% | 8.73% | 0.212 |
| Consumption of medical cannabis | 14.89% | 15.00% | 10.94% | 18.67% | 15.80% | 0.517 |
| Others | 31.91% | 32.00% | 35.94% | 31.93% | 32.55% | 0.941 |
| **Treatment expenditure per year** |  |  |  |  |  |  |
| Mean | 1,663.19 | 1,889.10 | 1,417.50 | 1,312.95 | 1,542.26 | 0.529 |
| Standard Deviation | 5,694.23 | 4,031.41 | 1,845.33 | 1,512.29 | 3,519.43 |  |
| Range | (0-55,000) | (0-34,000) | (20-12,000) | (0-10,000) | (0-55,000) |  |
| **Insurance** |  |  |  |  |  |  |
| RAMQ (carte soleil) | 39.36% | 36.00% | 45.31% | 37.35% | 38.68% | 0.634 |
| Private insurance | 50.00% | 56.00% | 40.63% | 52.41% | 50.94% |  |
| No insurance | 10.64% | 8.00% | 14.06% | 10.24% | 10.38% |  |
| **Do you suffer from a disease or a physical or mental problem that reduces your quality of life (e.g., diabetes, cancer, osteoarthritis)?** | | | | | |  |
| Yes | 63.83% | 84.00% | 60.94% | 63.25% | 67.92% | **0.001** |
| No | 36.17% | 16.00% | 39.06% | 36.75% | 32.08% |  |
|  |  |  |  |  |  |  |
| Tiredness | 75.00% | 73.81% | 71.79% | 75.24% | 74.31% | 0.977 |
| Insomnia | 50.00% | 63.10% | 71.79% | 57.14% | 59.38% | 0.146 |
| Pain | 90.00% | 95.24% | 84.62% | 84.76% | 88.89% | **0.084** |
| Anxiety/stress | 55.00% | 58.33% | 58.97% | 51.43% | 55.21% | 0.762 |
| Depression | 36.67% | 41.67% | 25.64% | 34.29% | 35.76% | 0.371 |
| Other mental disorder | 6.67% | 8.33% | 5.13% | 4.76% | 6.25% | 0.799 |
| Osteoarthritis | 60.00% | 64.29% | 66.67% | 65.71% | 64.24% | 0.880 |
| Arthritis | 16.67% | 14.29% | 23.08% | 15.24% | 16.32% | 0.647 |
| Unintentional injury | 5.00% | 3.57% | 2.56% | 9.52% | 5.90% | 0.322 |
| Musculoskeletal problem | 30.00% | 30.95% | 25.64% | 31.43% | 30.21% | 0.923 |
| Disease of the central nervous system | 8.33% | 15.48% | 12.82% | 5.71% | 10.07% | 0.132 |
| Thyroid problem | 28.33% | 15.48% | 5.13% | 20.95% | 18.75% | **0.026** |
| Other endocrin problem | 8.33% | 8.33% | 0.00% | 2.86% | 5.21% | **0.090** |
| Genital-urinary problem | 11.67% | 10.71% | 7.69% | 6.67% | 9.03% | 0.660 |
| Hypertension | 38.33% | 38.10% | 51.28% | 27.62% | 36.11% | **0.059** |
| Cardiac disease | 11.67% | 7.14% | 10.26% | 1.90% | 6.60% | **0.037** |
| Stroke | 0.00% | 2.38% | 0.00% | 0.95% | 1.04% | 0.749 |
| Digestive disorder | 36.67% | 22.62% | 23.08% | 25.71% | 26.74% | 0.254 |
| Other gastrointestinal problem | 21.67% | 21.43% | 15.38% | 20.95% | 20.49% | 0.865 |
| Diabetes | 30.00% | 20.24% | 30.77% | 16.19% | 22.22% | 0.105 |
| Cancer/tumor | 10.00% | 0.00% | 2.56% | 0.95% | 2.78% | **0.002** |
| Chronic Obstructive Pulmonary Disease (COPD) | 1.67% | 1.19% | 2.56% | 1.90% | 1.74% | 0.928 |
| Other breathing problems (asthma, emphysema) | 20.00% | 11.90% | 23.08% | 14.29% | 15.97% | 0.327 |
| Other medical disorder | 35.00% | 38.10% | 51.28% | 45.71% | 42.01% | 0.295 |
| **Health status** |  |  |  |  |  |  |
| Mean | 3.45 | 3.62 | 3.64 | 3.42 | 3.50 | 0.161 |
| Standard Deviation | 0.91 | 0.89 | 0.84 | 0.91 | 0.90 |  |
| Range | (1-5) | (1-5) | (2-5) | (1-5) | (1-5) |  |
|  |  |  |  |  |  |  |
| Excellent | 1.06% | 1.00% | 0.00% | 0.60% | 0.71% | 0.269 |
| Very good | 12.77% | 5.00% | 9.38% | 15.06% | 11.32% |  |
| Good | 39.36% | 44.00% | 31.25% | 38.55% | 38.92% |  |
| Fair | 34.04% | 31.00% | 45.31% | 33.73% | 34.91% |  |
| Poor | 12.77% | 19.00% | 14.06% | 12.05% | 14.15% |  |
| **Satisfaction with health** |  |  |  |  |  |  |
| Mean | 4.31 | 3.59 | 3.95 | 4.40 | 4.12 | **0.046** |
| Standard Deviation | 2.53 | 2.32 | 2.49 | 2.34 | 2.42 |  |
| Range | (0-9) | (0-9) | (0-9) | (0-10) | (0-10) |  |
| **Satisfaction with life** |  |  |  |  |  |  |
| Mean | 5.63 | 5.45 | 4.66 | 5.86 | 5.53 | **0.008** |
| Standard Deviation | 2.33 | 2.40 | 2.67 | 2.32 | 2.42 |  |
| Range | (0-10) | (0-10) | (0-9) | (0-10) | (0-10) |  |
| **Willingness to take risks** |  |  |  |  |  |  |
| Mean | 4.18 | 4.32 | 4.47 | 4.32 | 4.31 | 0.916 |
| Standard Deviation | 2.44 | 2.51 | 2.57 | 2.51 | 2.50 |  |
| Range | (0-9) | (0-9) | (0-10) | (0-9) | (0-10) |  |
| **Roland-Morris Disability Questionnaire**^2^ |  |  |  |  |  |  |
| Mean | 10.07 | 10.68 | 10.87 | 9.27 | 10.02 | 0.116 |
| Standard Deviation | 5.25 | 5.11 | 5.27 | 5.10 | 5.18 |  |
| Range | (1-23) | (1-23) | (1-22) | (1-21) | (1-23) |  |
| **Oswestry Disability Index**^3^ |  |  |  |  |  |  |
| Mean | 42.86 | 44.84 | 47.55 | 38.98 | 42.49 | **0.001** |
| Standard Deviation | 16.12 | 13.69 | 13.95 | 15.73 | 15.36 |  |
| Range | (6-80) | (12-92) | (6-86) | (6-76) | (6-92) |  |
|  |  |  |  |  |  |  |
| Minimal Disability (0-20) | 11.11% | 3.23% | 1.89% | 17.01% | 10.16% | **0.002** |
| Moderate Disability (21-40) | 30.86% | 38.71% | 24.53% | 38.78% | 35.03% |  |
| Severe Disability (41-60) | 45.68% | 45.16% | 60.38% | 35.37% | 43.58% |  |
| Crippled (61-80) | 12.35% | 11.83% | 11.32% | 8.84% | 10.70% |  |
| Bed-Bound (81-100) | 0.00% | 1.08% | 1.89% | 0.00% | 0.53% |  |
| **QALY SF-6Dv2bis**^4^ |  |  |  |  |  |  |
| Mean | 0.328 | 0.294 | 0.316 | 0.363 | 0.332 | 0.141 |
| Standard Deviation | 0.228 | 0.240 | 0.246 | 0.220 | 0.231 |  |
| Range | (-0.317-0.843) | (-0.373-0.759) | (-0.320-0.840) | (-0.320-0.882) | (-0.373-0.882) |  |
| **QALY EQ-5D-5L**^5^ |  |  |  |  |  |  |
| Mean | 0.605 | 0.543 | 0.508 | 0.603 | 0.575 | **0.019** |
| Standard Deviation | 0.229 | 0.223 | 0.234 | 0.225 | 0.229 |  |
| Range | (0.045-0.905) | (-0.072-0.905) | (-0.064-0.867) | (-0.044-0.905) | (-0.072-0.905) |  |
| **EQ-5D-5L-VAS** |  |  |  |  |  |  |
| Mean | 52.77 | 49.01 | 42.75 | 56.86 | 52.00 | **<.001** |
| Standard deviation | 25.23 | 21.94 | 21.40 | 20.09 | 22.39 |  |
| First quartile | 31 | 33 | 26 | 46 | 34 |  |
| Median | 55 | 49 | 40 | 58.5 | 53 |  |
| Third quartile | 71 | 61 | 58 | 71 | 69 |  |
| Range | (0-100) | (0-100) | (2-86) | (0-100) | (0-100) |  |
|  |  |  |  |  |  |  |

Notes: CEGEP: Collège d’enseignement général et professionnel; RAMQ: Régie de l’assurance maladie du Québec.

^1^The $p$-values refer to tests between classes using one-way analysis of variance, Kruskal-Wallis H test, Bartlett’s test for equality of variances, Fisher's exact test, and Chi2 test of independence.

^2^The Roland-Morris Disability Questionnaire is a 24-item questionnaire measuring self-assessed back pain with a yes/no format and ranging from 0 (no back pain) to 24 (worst back pain).

^3^The Oswestry Disability Index is a 10-item questionnaire with a 6-point Likert scaling and rescaled from 0 to 100.

^4^The Short Form 6-Dimension version 2 (SF-6Dv2) is a 6-dimension generic health-related quality of life questionnaire.

^5^The EuroQol 5-Dimension 5-Level (EQ-5D-5L) is a 5-dimension generic health-related quality of life questionnaire.

**Appendix 7 – Zero-centered utility values produced by the latent class analysis (**$\boldsymbol{c}\mathbf{=4}$**) and conditional relative importance of attributes**

| **Latent class logit** | | | | |
| --- | --- | --- | --- | --- |
| **Attributes** | **Class 1** | **Class 2** | **Class 3** | **Class 4** |
| **(Standardized utilities**^1^**)** |  |  |  |  |
|  |  |  |  |  |
| None^2^ | 94.09 | -390.66 | 274.99 | -227.02 |
|  |  |  |  |  |
| Treatment modality |  |  |  |  |
| Corticosteroid injections | -58.17 | 133.98 | 132.81 | -226.02 |
| Supervised body-mind physical activities | 15.54 | -49.41 | 21.53 | 84.46 |
| Supervised sports physical activities | 6.65 | -11.02 | 17.79 | 40.94 |
| Physical manipulations | 114.22 | -6.27 | -17.23 | 69.06 |
| Self-management courses | -6.18 | -36.21 | 58.35 | 63.29 |
| Psychotherapy | -72.07 | -31.06 | -213.24 | -31.73 |
| Pain reduction |  |  |  |  |
| None to very slight | -80.76 | -78.87 | 10.86 | -87.90 |
| Slight | -49.63 | -4.27 | 32.62 | -21.56 |
| Reduced by half | 58.20 | 38.81 | 8.33 | 36.90 |
| No pain | 72.19 | 44.33 | -51.81 | 72.57 |
| Onset of treatment efficacy |  |  |  |  |
| 1 month | 35.92 | 20.02 | -8.74 | 11.22 |
| 6 months | -15.20 | 16.01 | -1.85 | 11.80 |
| 12 months | -20.72 | -36.03 | 10.60 | -23.02 |
| Duration effectiveness |  |  |  |  |
| Effective for 2 months | -53.92 | -32.96 | 46.66 | -38.46 |
| Effective for 6 months | 21.98 | -20.23 | -32.43 | 5.24 |
| Effective for 12 months | 31.93 | 53.18 | -14.23 | 33.22 |
| Difficulties with daily activities |  |  |  |  |
| As many difficulties as before | -65.29 | -93.68 | -14.36 | -30.74 |
| Fewer difficulties as before | 29.55 | 18.61 | 0.98 | 19.24 |
| No difficulties | 35.74 | 75.06 | 13.38 | 11.50 |
| Sleep problems |  |  |  |  |
| As many problems as before | -59.09 | -27.05 | 40.88 | -29.31 |
| Fewer problems than before | 27.88 | 14.76 | 22.20 | 14.20 |
| No problems | 31.20 | 12.29 | -63.07 | 15.11 |
| Knowledge of his/her body and pain location |  |  |  |  |
| Same knowledge as before | -10.34 | -24.32 | 12.59 | -11.32 |
| Knowledge somewhat better than before | -6.26 | 7.96 | 13.41 | -5.52 |
| Knowledge much better than before | 16.60 | 16.36 | -26.00 | 16.83 |
|  |  |  |  |  |
| **Number of observations** |  |  |  |  |
| Absolute share (%) | 36.56 | 17.45 | 26.18 | 19.81 |
| Class share (%) | 23.00 | 25.30 | 15.00 | 36.70 |
|  |  |  |  |  |
| Observations | 424 | | | |
| McFadden R2 | 23.86 | | | |
| Chi2 | 2,501.80 | | | |
| Log likelihood | -3,990.45 | | | |
| Log likelihood (null) | -5,243.68 | | | |
| AIC | 8,143.55 | | | |
| BIC | 8,654.74 | | | |
| **Relative weight of attributes (%)** | | | | |
|  |  |  |  |  |
| Treatment modality | 26.61 | 26.20 | 49.44 | 44.36 |
| Pain reduction | 21.85 | 17.60 | 12.06 | 22.92 |
| Onset of treatment efficacy | 8.09 | 8.01 | 2.76 | 4.97 |
| Duration effectiveness | 12.26 | 12.31 | 11.30 | 10.24 |
| Difficulties with daily activities | 14.43 | 24.11 | 3.96 | 7.14 |
| Sleep problems | 12.90 | 5.97 | 14.85 | 6.35 |
| Knowledge of his/her body and pain location | 3.85 | 5.81 | 5.63 | 4.02 |
|  |  |  |  |  |

Notes: AIC: Akaike Information Criterion; BIC: Bayesian Information Criterion.

^1^A value close to zero indicates an absence of preference: the further the value is from zero, the greater the preference.

^2^Coefficient of the opt-out option.

# **Appendix 8 – Models’ results graphical representations**

# **Appendix 9 – Density of part worth utilities with one-sample multivariate tests of means (**$\mathbf{H}_{\boldsymbol{0}}\boldsymbol{:\mu=0}$**), Hierarchical Bayesian model (**$\boldsymbol{n=424}$**)**


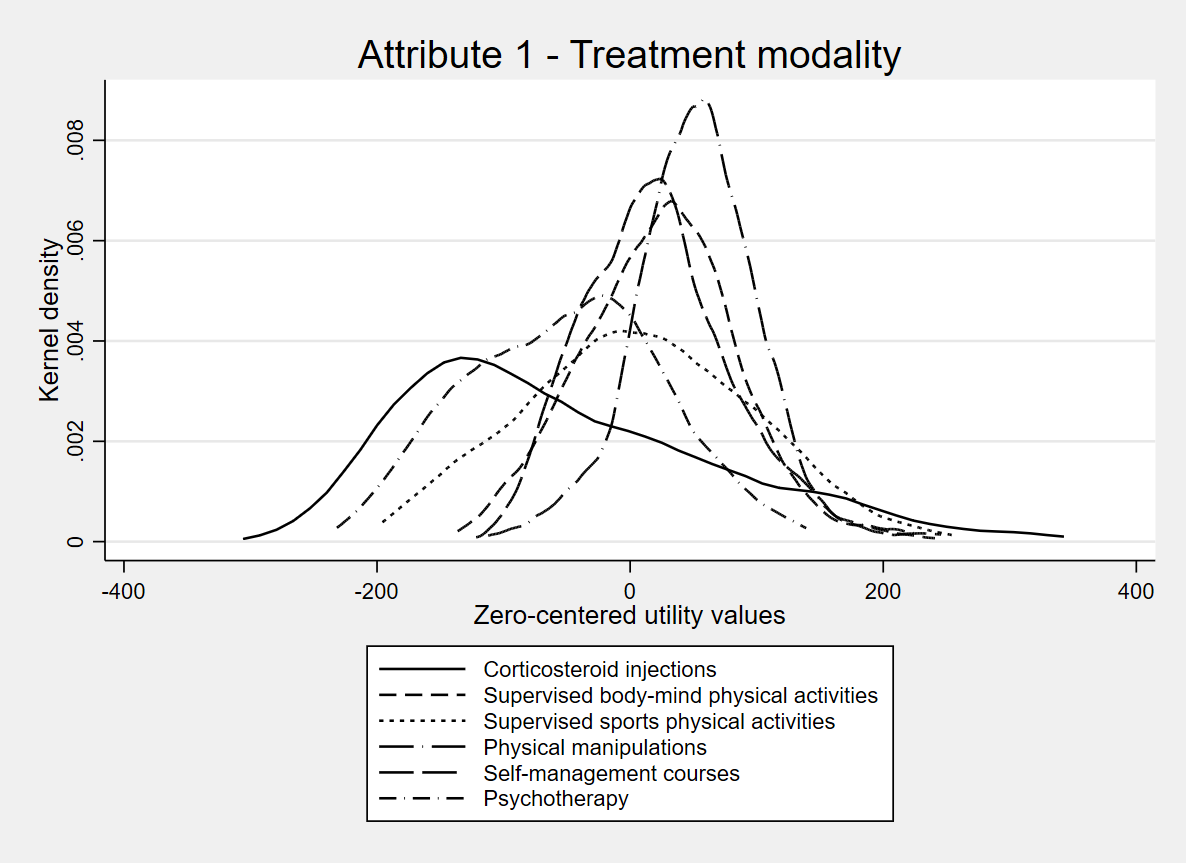


Hotelling $T^{2}=638.74$, $p<0.001$.


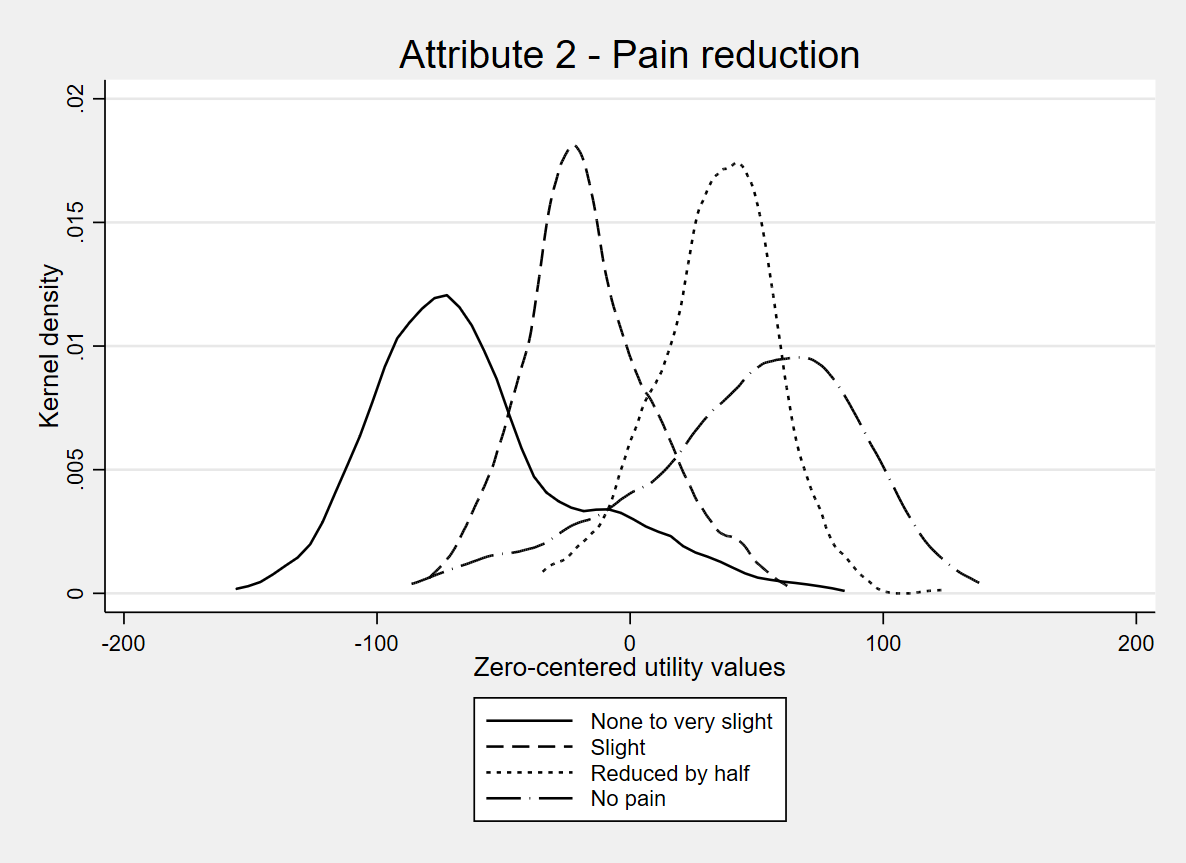


Hotelling $T^{2}=1,134.71$, $p<0.001$.


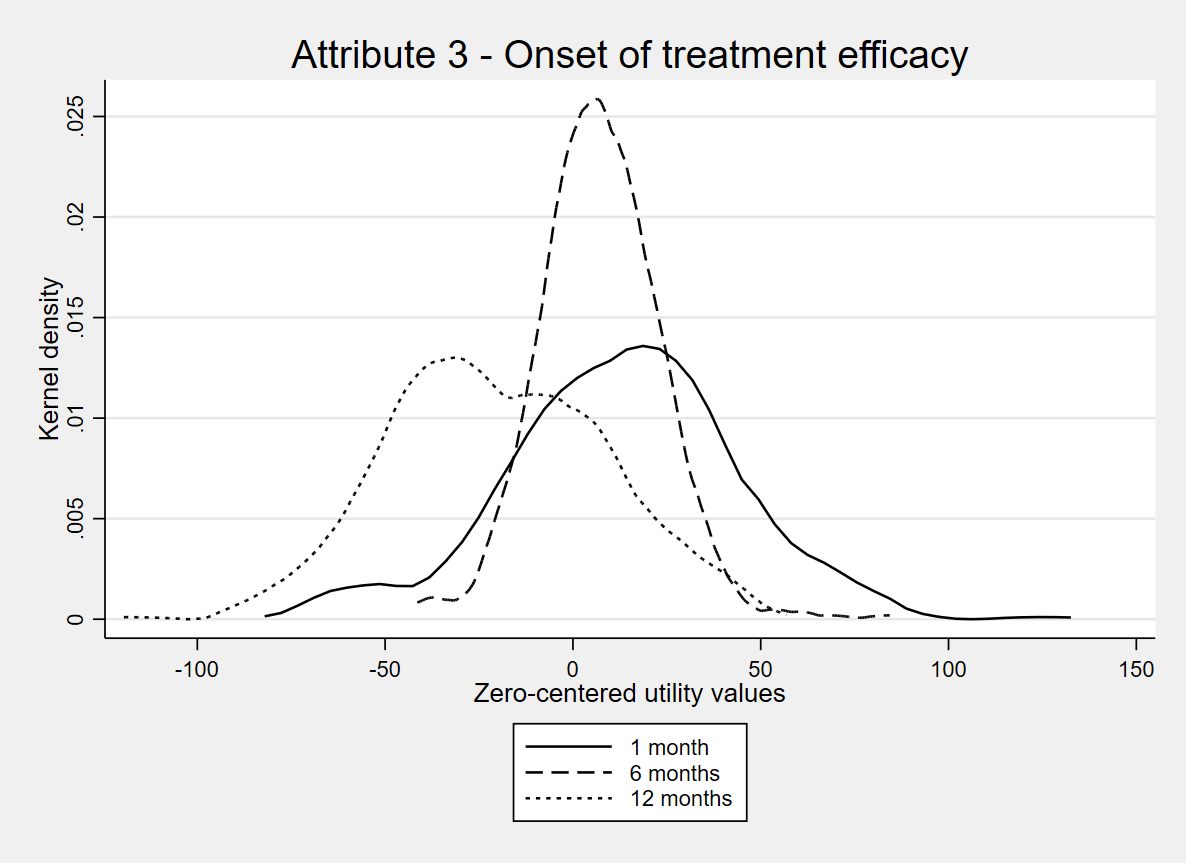


Hotelling $T^{2}=258.15$, $p<0.001$.


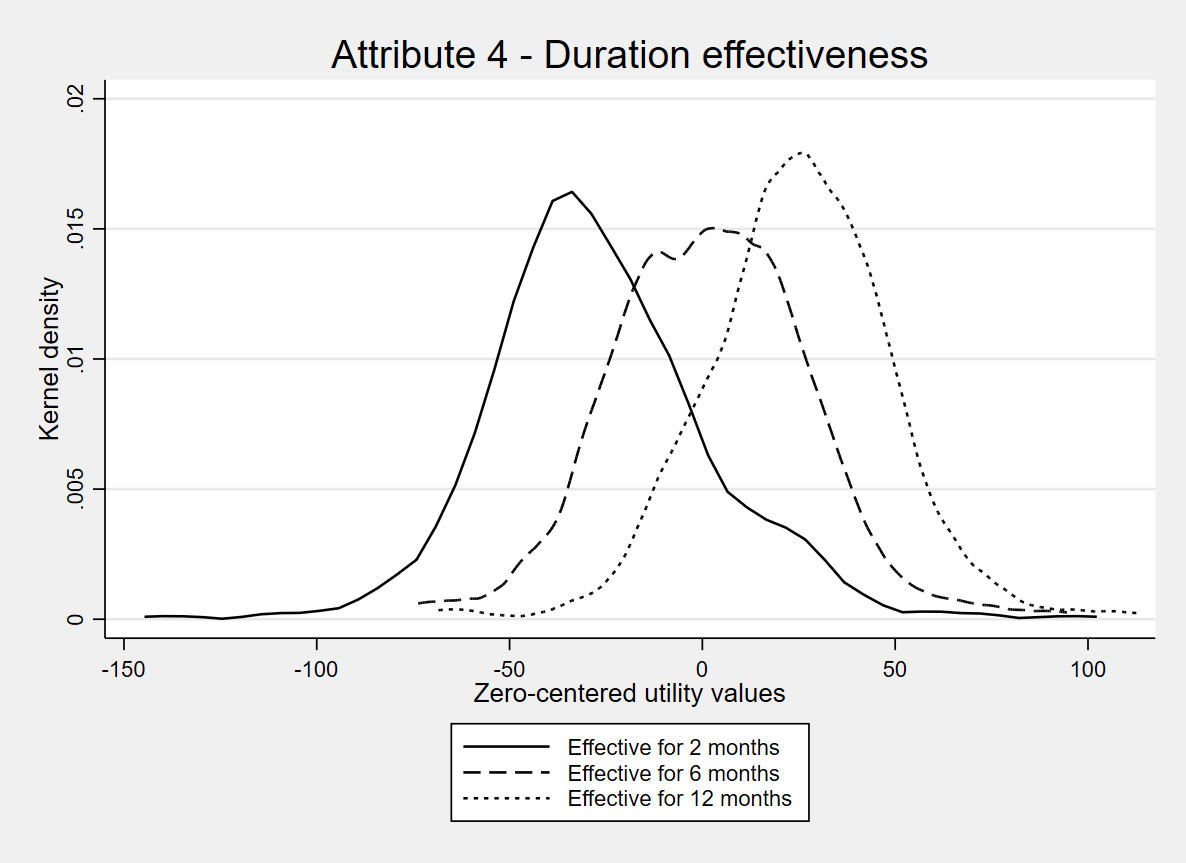


Hotelling $T^{2}=549.23$, $p<0.001$.


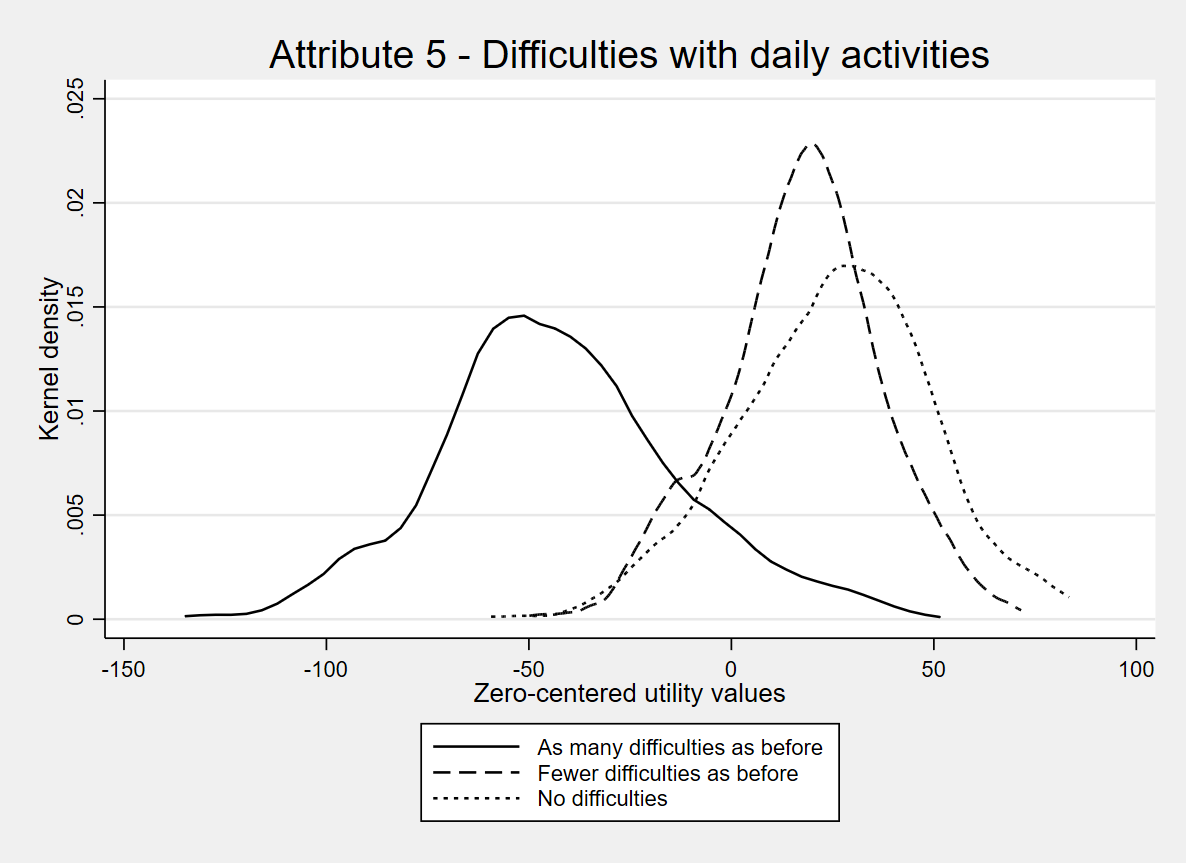


Hotelling $T^{2}=902.24$, $p<0.001$.


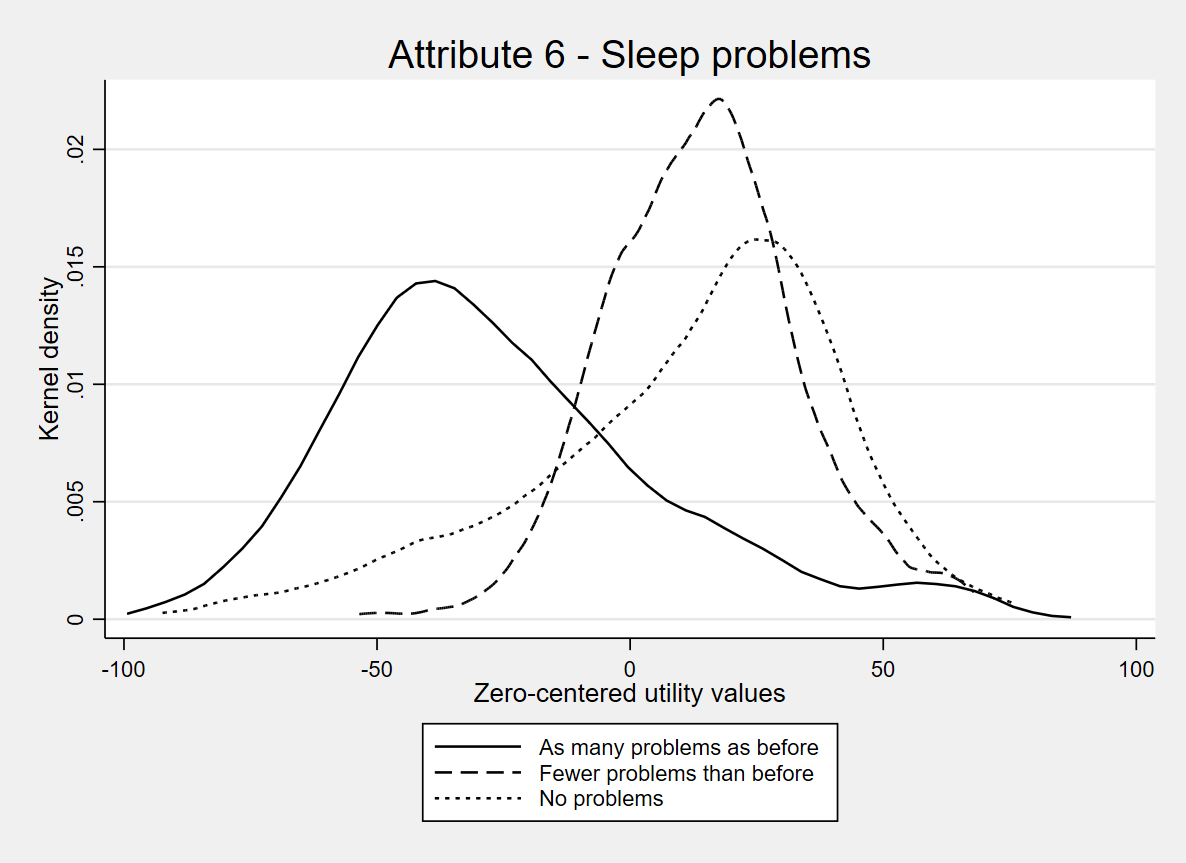


Hotelling $T^{2}=369.33$, $p<0.001$.


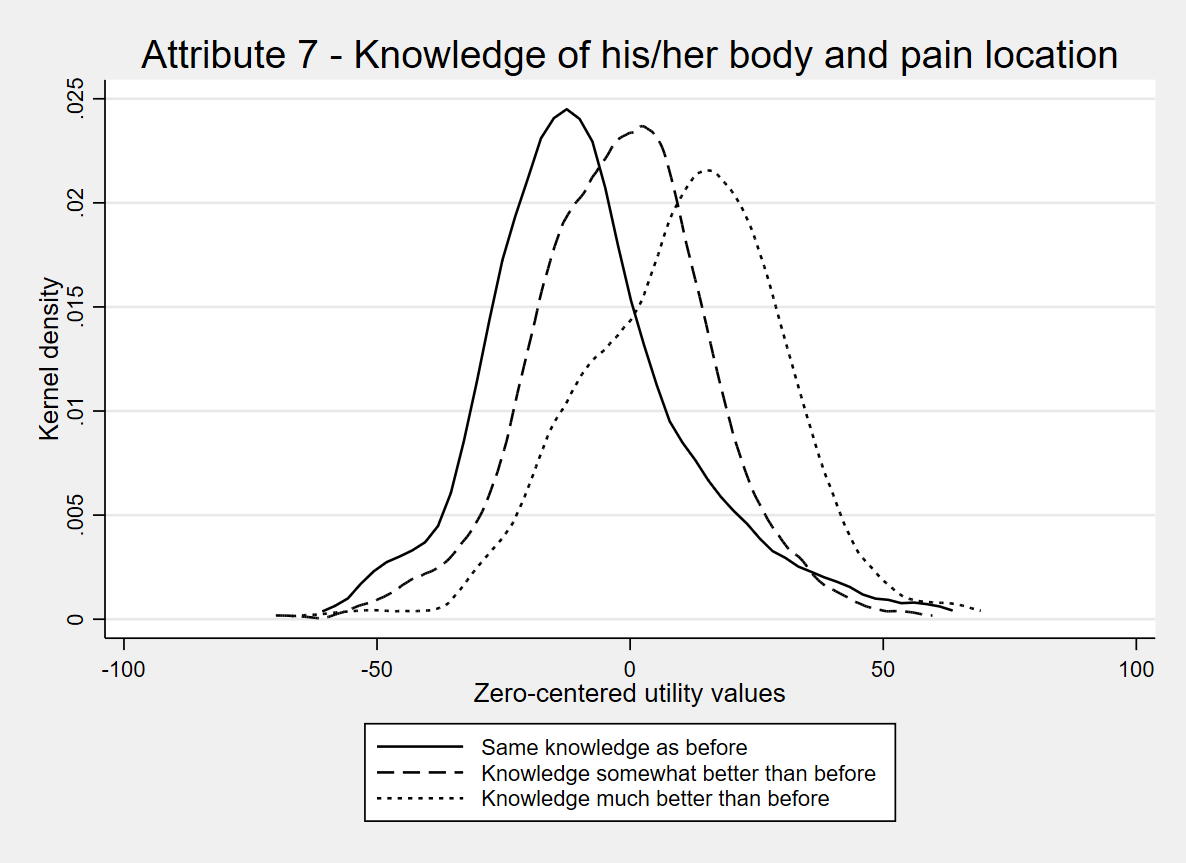


Hotelling $T^{2}=135.51$, $p<0.001$.

# **Appendix 10 –** **Respondent’s personal ranking of attributes and treatment modalities in total and per class from the latent class analysis (**$\boldsymbol{c=4}$ **and** $\boldsymbol{c=6}$**) (“What were the most important dimensions for you in choosing? Please rank the dimensions from 1 (the most important) to 7 (the least important).”; (“Please rank the 6 LBP treatments proposed from the most important (1) to the least important (6) in your opinion.**”**))**

After the choice-based exercise, respondents were asked to rank in order of importance the seven attributes. Results of this simple question indicated that pain reduction seemed a lot more important than the treatment modality (mean difference, $p<0.001$) and was followed by the effectiveness duration. Differences within attributes ranking (from this same question) between latent classes appeared only in the treatment modality (i.e., it was more important for class 2, $p=0.010$), in the onset of treatment modality (i.e., it was more important for class 1, $p=0.012$), and in the sleep problem (it was more important for class 6, $p=0.066$). Within treatment modality (i.e., another simple question to rank by order of importance the six treatments), corticosteroid injections were ranked first by class 3 ($p<0.001$), while physical manipulations were ranked first by almost all other classes ($p=0.029$), and psychotherapy was generally ranked as the least preferred attribute ($p=0.738$).

| **Respondent's personal ranking of attributes** | **Class 1** | **Class 2** | **Class 3** | **Class 4** | **Class 5** | **Class 6** | **Total** | **P-value**^1^ |
| --- | --- | --- | --- | --- | --- | --- | --- | --- |
|  |  |  |  |  |  |  |  |  |
| **Treatment modality** |  |  |  |  |  |  |  |  |
| Mean | 4.70 | 3.52 | 4.26 | 4.81 | 4.24 | 4.90 | 4.37 | **0.010** |
| Standard deviation | 1.81 | 2.39 | 1.72 | 1.97 | 2.25 | 1.58 | 2.09 |  |
| Range | (1-7) | (1-7) | (1-7) | (1-7) | (1-7) | (1-7) | (1-7) |  |
| **Pain reduction** |  |  |  |  |  |  |  |  |
| Mean | 1.54 | 1.71 | 1.39 | 1.48 | 1.62 | 1.46 | 1.56 | 0.542 |
| Standard deviation | 0.96 | 1.15 | 0.85 | 0.83 | 0.94 | 0.78 | 0.93 |  |
| Range | (1-4) | (1-5) | (1-5) | (1-4) | (1-6) | (1-4) | (1-6) |  |
| **Onset of treatment efficacy** |  |  |  |  |  |  |  |  |
| Mean | 3.41 | 3.75 | 4.28 | 3.32 | 3.94 | 4.07 | 3.80 | **0.012** |
| Standard deviation | 1.77 | 1.54 | 1.47 | 1.42 | 1.57 | 1.59 | 1.58 |  |
| Range | (1-7) | (1-7) | (2-7) | (1-6) | (1-7) | (1-7) | (1-7) |  |
| **Effectiveness duration** |  |  |  |  |  |  |  |  |
| Mean | 3.37 | 3.81 | 3.62 | 3.32 | 3.49 | 3.76 | 3.53 | 0.429 |
| Standard deviation | 1.31 | 1.58 | 1.55 | 1.44 | 1.34 | 1.69 | 1.45 |  |
| Range | (2-6) | (2-7) | (1-7) | (1-7) | (1-7) | (1-7) | (1-7) |  |
| **Difficulties with daily activities** |  |  |  |  |  |  |  |  |
| Mean | 4.15 | 4.35 | 4.23 | 4.13 | 4.26 | 4.10 | 4.22 | 0.962 |
| Standard deviation | 1.69 | 1.26 | 1.78 | 1.34 | 1.52 | 1.79 | 1.54 |  |
| Range | (2-7) | (2-7) | (1-7) | (1-6) | (1-7) | (1-7) | (1-7) |  |
| **Sleep problems** |  |  |  |  |  |  |  |  |
| Mean | 5.13 | 5.54 | 5.03 | 4.98 | 5.04 | 4.39 | 5.03 | **0.066** |
| Standard deviation | 1.60 | 1.46 | 1.90 | 1.60 | 1.73 | 1.86 | 1.71 |  |
| Range | (1-7) | (2-7) | (1-7) | (1-7) | (1-7) | (1-7) | (1-7) |  |
| **Knowledge of his/her body and pain location** | |  |  |  |  |  |  |  |
| Mean | 5.70 | 5.31 | 5.21 | 5.95 | 5.42 | 5.32 | 5.49 | 0.245 |
| Standard deviation | 1.58 | 1.73 | 1.94 | 1.60 | 1.86 | 2.04 | 1.81 |  |
| Range | (1-7) | (1-7) | (1-7) | (1-7) | (1-7) | (1-7) | (1-7) |  |
|  |  |  |  |  |  |  |  |  |

Note: the lower the mean is, the higher the attribute is considered as important

^1^The $p$-values refer to tests between classes using one-way analysis of variance, Kruskal-Wallis H test, Bartlett’s test for equality of variances, Fisher's exact test, and Chi2 test of independence.

| **Respondent's personal ranking of attributes** | **Class 1** | **Class 2** | **Class 3** | **Class 4** | **Total** | **P-value**^1^ |
| --- | --- | --- | --- | --- | --- | --- |
|  |  |  |  |  |  |  |
| **Treatment modality** |  |  |  |  |  |  |
| Mean | 4.34 | 4.84 | 4.72 | 3.96 | 4.37 | **0.029** |
| Standard deviation | 2.14 | 1.85 | 1.69 | 2.26 | 2.09 |  |
| Range | (1-7) | (1-7) | (1-7) | (1-7) | (1-7) |  |
| **Pain reduction** |  |  |  |  |  |  |
| Mean | 1.57 | 1.44 | 1.45 | 1.66 | 1.56 | 0.232 |
| Standard deviation | 0.97 | 0.88 | 0.75 | 0.98 | 0.93 |  |
| Range | (1-5) | (1-5) | (1-4) | (1-6) | (1-6) |  |
| **Onset of treatment efficacy** |  |  |  |  |  |  |
| Mean | 3.40 | 3.72 | 4.09 | 3.97 | 3.80 | **0.029** |
| Standard deviation | 1.34 | 1.71 | 1.62 | 1.56 | 1.58 |  |
| Range | (1-6) | (1-7) | (1-7) | (1-7) | (1-7) |  |
| **Effectiveness duration** |  |  |  |  |  |  |
| Mean | 3.44 | 3.35 | 3.79 | 3.60 | 3.53 | 0.275 |
| Standard deviation | 1.41 | 1.31 | 1.65 | 1.47 | 1.45 |  |
| Range | (1-7) | (1-7) | (1-7) | (1-7) | (1-7) |  |
| **Difficulties with daily activities** |  |  |  |  |  |  |
| Mean | 4.30 | 4.03 | 4.11 | 4.32 | 4.22 | 0.457 |
| Standard deviation | 1.44 | 1.62 | 1.74 | 1.46 | 1.54 |  |
| Range | (1-7) | (1-7) | (1-7) | (1-7) | (1-7) |  |
| **Sleep problems** |  |  |  |  |  |  |
| Mean | 5.09 | 4.95 | 4.64 | 5.19 | 5.03 | 0.218 |
| Standard deviation | 1.58 | 1.62 | 1.87 | 1.75 | 1.71 |  |
| Range | (1-7) | (1-7) | (1-7) | (1-7) | (1-7) |  |
| **Knowledge of his/her body and pain location** |  |  |  |  |  |  |
| Mean | 5.85 | 5.67 | 5.19 | 5.29 | 5.49 | **0.055** |
| Standard deviation | 1.72 | 1.61 | 2.05 | 1.85 | 1.81 |  |
| Range | (1-7) | (1-7) | (1-7) | (1-7) | (1-7) |  |
|  |  |  |  |  |  |  |

Note: the lower the mean is, the higher the attribute is considered as important

^1^The $p$-values refer to tests between classes using one-way analysis of variance, Kruskal-Wallis H test, Bartlett’s test for equality of variances, Fisher's exact test, and Chi2 test of independence.

| **Respondent's personal ranking of treatment modalities** | **Class 1** | **Class 2** | **Class 3** | **Class 4** | **Class 5** | **Class 6** | **Total** | **P-value**^1^ |
| --- | --- | --- | --- | --- | --- | --- | --- | --- |
|  |  |  |  |  |  |  |  |  |
| **Corticosteroid injections** |  |  |  |  |  |  |  |  |
| Mean | 2.64 | 5.55 | 2.78 | 4.02 | 4.75 | 3.87 | 4.20 | **<0.001** |
| Standard deviation | 1.75 | 0.97 | 2.06 | 1.98 | 1.74 | 2.15 | 2.00 |  |
| Range | (1-6) | (1-6) | (1-6) | (1-6) | (1-6) | (1-6) | (1-6) |  |
| **Supervised body-mind physical activities** | |  |  |  |  |  |  |  |
| Mean | 4.10 | 2.64 | 3.70 | 3.45 | 3.14 | 3.46 | 3.33 | **<0.001** |
| Standard deviation | 1.50 | 1.21 | 1.53 | 1.61 | 1.51 | 1.35 | 1.52 |  |
| Range | (1-6) | 1 | (1-6) | (1-6) | (1-6) | (1-6) | (1-6) |  |
| **Supervised sports physical activities** |  |  |  |  |  |  |  |  |
| Mean | 3.98 | 3.09 | 4.22 | 3.94 | 3.43 | 3.80 | 3.65 | **0.005** |
| Standard deviation | 1.57 | 1.63 | 1.60 | 1.61 | 1.64 | 1.58 | 1.64 |  |
| Range | 2 | (1-6) | (1-6) | (1-6) | (1-6) | (1-6) | (1-6) |  |
| **Physical manipulations** |  |  |  |  |  |  |  |  |
| Mean | 2.57 | 2.66 | 2.87 | 2.07 | 2.60 | 3.08 | 2.59 | **0.029** |
| Standard deviation | 1.70 | 1.49 | 1.69 | 1.27 | 1.54 | 1.53 | 1.54 |  |
| Range | (1-6) | (1-6) | (1-6) | (1-6) | (1-6) | (1-6) | (1-6) |  |
| **Self-management courses** |  |  |  |  |  |  |  |  |
| Mean | 3.33 | 2.83 | 3.30 | 3.15 | 2.99 | 2.69 | 3.03 | 0.224 |
| Standard deviation | 1.41 | 1.42 | 1.31 | 1.32 | 1.45 | 1.51 | 1.42 |  |
| Range | (1-6) | (1-6) | (1-6) | (1-6) | (1-6) | (1-6) | (1-6) |  |
| **Psychotherapy** |  |  |  |  |  |  |  |  |
| Mean | 4.38 | 4.23 | 4.14 | 4.39 | 4.09 | 4.10 | 4.20 | 0.738 |
| Standard deviation | 1.50 | 1.27 | 1.53 | 1.37 | 1.42 | 1.71 | 1.44 |  |
| Range | (1-6) | (1-6) | (1-6) | (1-6) | (1-6) | (1-6) | (1-6) |  |
|  |  |  |  |  |  |  |  |  |

Note: the lower the mean is, more appreciated is the treatment modality.

^1^The $p$-values refer to tests between classes using one-way analysis of variance, Kruskal-Wallis H test, Bartlett’s test for equality of variances, Fisher's exact test, and Chi2 test of independence.

| **Respondent's personal ranking of treatment modalities** | **Class 1** | **Class 2** | **Class 3** | **Class 4** | **Total** | **P-value**^1^ |
| --- | --- | --- | --- | --- | --- | --- |
|  |  |  |  |  |  |  |
| **Corticosteroid injections** |  |  |  |  |  |  |
| Mean | 4.36 | 2.90 | 3.55 | 5.15 | 4.20 | **<.001** |
| Standard deviation | 1.87 | 1.87 | 2.24 | 1.48 | 2.00 |  |
| Range | (1-6) | (1-6) | (1-6) | (1-6) | (1-6) |  |
| **Supervised body-mind physical activities** |  |  |  |  |  |  |
| Mean | 3.35 | 4.07 | 3.43 | 2.82 | 3.33 | **<.001** |
| Standard deviation | 1.63 | 1.53 | 1.37 | 1.30 | 1.52 |  |
| Range | (1-6) | (1-6) | (1-6) | (1-6) | (1-6) |  |
| **Supervised sports physical activities** |  |  |  |  |  |  |
| Mean | 3.78 | 3.90 | 3.98 | 3.30 | 3.65 | **0.010** |
| Standard deviation | 1.60 | 1.58 | 1.54 | 1.69 | 1.64 |  |
| Range | (1-6) | (1-6) | (1-6) | (1-6) | (1-6) |  |
| **Physical manipulations** |  |  |  |  |  |  |
| Mean | 2.15 | 2.58 | 3.35 | 2.58 | 2.59 | **<.001** |
| Standard deviation | 1.37 | 1.65 | 1.53 | 1.47 | 1.54 |  |
| Range | (1-6) | (1-6) | (1-6) | (1-6) | (1-6) |  |
| **Self-management courses** |  |  |  |  |  |  |
| Mean | 3.02 | 3.26 | 2.67 | 3.02 | 3.03 | 0.119 |
| Standard deviation | 1.28 | 1.43 | 1.47 | 1.45 | 1.42 |  |
| Range | (1-6) | (1-6) | (1-6) | (1-6) | (1-6) |  |
| **Psychotherapy** |  |  |  |  |  |  |
| Mean | 4.35 | 4.29 | 4.02 | 4.12 | 4.20 | 0.506 |
| Standard deviation | 1.35 | 1.49 | 1.68 | 1.38 | 1.44 |  |
| Range | (1-6) | (1-6) | (1-6) | (1-6) | (1-6) |  |
|  |  |  |  |  |  |  |

Note: the lower the mean is, more appreciated is the treatment modality.

^1^The $p$-values refer to tests between classes using one-way analysis of variance, Kruskal-Wallis H test, Bartlett’s test for equality of variances, Fisher's exact test, and Chi2 test of independence.

# **Appendix 11 – Discrete choice experiment perception (total and per group from the latent class analysis,** $\boldsymbol{c=4}$ **and** $\boldsymbol{c=6}$**)**

| **DCE perception** | **Class 1** | **Class 2** | **Class 3** | **Class 4** | **Class 5** | **Class 6** | **Total** | **P-value^1^** |
| --- | --- | --- | --- | --- | --- | --- | --- | --- |
|  |  |  |  |  |  |  |  |  |
| **Observations** | 48 | 57 | 43 | 67 | 159 | 50 | 424 |  |
|  | 11.32% | 13.44% | 10.14% | 15.80% | 37.50% | 11.79% | 100.00% |  |
|  |  |  |  |  |  |  |  |  |
| **Number of treatment refusals (ranging from 0 to 11)** |  |  |  |  |  |  |  |  |
| Mean | 0.73 | 3.19 | 1.65 | 5.70 | 0.17 | 7.30 | 2.50 | **<.001** |
| Standard deviation | 1.03 | 1.60 | 1.29 | 2.10 | 0.41 | 2.91 | 3.04 |  |
| Range | (0-3) | (1-7) | (0-4) | (1-10) | (0-2) | (0-11) | (0-11) |  |
| **Choice certainty score (ranging from 12 to 132)** | |  |  |  |  |  |  |  |
| Mean | 96.72 | 105.80 | 103.77 | 106.92 | 104.92 | 102.86 | 104.04 | 0.211 |
| Standard deviation | 21.28 | 22.45 | 24.04 | 19.13 | 20.26 | 25.38 | 21.56 |  |
| Range | (35-132) | (13-132) | (45-132) | (54-132) | (24-132) | (33-132) | (13-132) |  |
| **Choice exercise difficulty** |  |  |  |  |  |  |  |  |
| Very easy | 0.00% | 6.00% | 5.13% | 1.59% | 6.21% | 11.90% | 5.19% | 0.124 |
| Easy | 21.74% | 26.00% | 20.51% | 20.63% | 26.90% | 14.29% | 23.12% |  |
| Neutral | 21.74% | 22.00% | 23.08% | 36.51% | 22.76% | 35.71% | 26.23% |  |
| Hard | 52.17% | 44.00% | 48.72% | 36.51% | 41.38% | 26.19% | 41.30% |  |
| Very hard | 4.35% | 2.00% | 2.56% | 4.76% | 2.76% | 11.90% | 4.16% |  |
| **Number of dimensions considered** |  |  |  |  |  |  |  |  |
| 1 | 2.17% | 0.00% | 2.56% | 0.00% | 2.07% | 2.38% | 1.56% | **0.004** |
| 2 | 15.22% | 22.00% | 15.38% | 7.94% | 8.97% | 9.52% | 11.95% |  |
| 3 | 45.65% | 28.00% | 38.46% | 34.92% | 35.86% | 28.57% | 35.32% |  |
| 4 or more | 34.78% | 30.00% | 15.38% | 41.27% | 42.76% | 33.33% | 36.10% |  |
| Not sure | 2.17% | 20.00% | 28.21% | 15.87% | 10.34% | 26.19% | 15.06% |  |
| **Quality of responses** |  |  |  |  |  |  |  |  |
| Very good | 19.57% | 29.17% | 20.51% | 29.03% | 27.78% | 26.83% | 26.32% | 0.329 |
| Good | 54.35% | 52.08% | 43.59% | 53.23% | 56.25% | 43.90% | 52.37% |  |
| Average | 26.09% | 18.75% | 35.90% | 17.74% | 15.97% | 29.27% | 21.32% |  |
| **Have been annoyed** |  |  |  |  |  |  |  |  |
| Yes | 39.13% | 27.08% | 23.08% | 25.81% | 20.14% | 36.59% | 26.32% | 0.102 |
| No | 60.87% | 72.92% | 76.92% | 74.19% | 79.86% | 63.41% | 73.68% |  |
| **Failed to the rationality test** |  |  |  |  |  |  |  |  |
| Yes | 31.25% | 0.00% | 37.21% | 0.00% | 1.26% | 50.00% | 13.68% | **<.001** |
| No | 68.75% | 100.00% | 62.79% | 100.00% | 98.74% | 50.00% | 86.32% |  |
| **Failed to the temporal consistency test** |  |  |  |  |  |  |  |  |
| Yes | 27.08% | 43.86% | 39.53% | 41.79% | 24.53% | 50.00% | 34.67% | **0.003** |
| No | 72.92% | 56.14% | 60.47% | 58.21% | 75.47% | 50.00% | 65.33% |  |
|  |  |  |  |  |  |  |  |  |

^1^The $p$-values refer to tests between classes using one-way analysis of variance, Kruskal-Wallis H test, Bartlett’s test for equality of variances, Fisher's exact test, and Chi2 test of independence.

| **DCE perception** | **Class 1** | **Class 2** | **Class 3** | **Class 4** | **Total** | **P-value**^1^ |
| --- | --- | --- | --- | --- | --- | --- |
|  |  |  |  |  |  |  |
| **Observations** |  |  |  |  |  |  |
| n | 94 | 100 | 64 | 166 | 424 | - |
| Class share | 22.17% | 23.58% | 15.09% | 39.15% | 100.00% |  |
| **Number of treatment refusals (ranging from 0 to 11)** |  |  |  |  |  |  |
| Mean | 5.03 | 0.60 | 6.70 | 0.60 | 2.50 | **<.001** |
| Standard deviation | 2.06 | 0.97 | 3.03 | 0.95 | 3.04 |  |
| Range | (1-10) | (0-3) | (0-11) | (0-3) | (0-11) |  |
| **Choice certainty score (ranging from 12 to 132)** |  |  |  |  |  |  |
| Mean | 104.68 | 99.21 | 104.72 | 106.52 | 104.04 | **0.073** |
| Standard deviation | 21.54 | 22.52 | 24.71 | 19.35 | 21.56 |  |
| Range | (13-132) | (35-132) | (33-132) | (24-132) | (13-132) |  |
| **Choice exercise difficulty** |  |  |  |  |  |  |
| Mean | 3.31 | 3.40 | 3.19 | 2.92 | 3.16 | **0.004** |
| Standard deviation | 0.89 | 0.85 | 1.12 | 1.05 | 1.00 |  |
| Range | (1-5) | (1-5) | (1-5) | (1-5) | (1-5) |  |
|  |  |  |  |  |  |  |
| Very easy | 2.35% | 1.04% | 9.26% | 8.00% | 5.19% | **<.001** |
| Easy | 16.47% | 18.75% | 14.81% | 32.67% | 23.12% |  |
| Neutral | 34.12% | 21.88% | 35.19% | 21.33% | 26.23% |  |
| Hard | 42.35% | 56.25% | 29.63% | 35.33% | 41.30% |  |
| Very hard | 4.71% | 2.08% | 11.11% | 2.67% | 4.16% |  |
| **Number of dimensions considered** |  |  |  |  |  |  |
| 1 | 0.00% | 2.08% | 1.85% | 2.00% | 1.56% | 0.113 |
| 2 | 12.94% | 15.63% | 7.41% | 10.67% | 11.95% |  |
| 3 | 30.59% | 42.71% | 31.48% | 34.67% | 35.32% |  |
| 4 or more | 37.65% | 30.21% | 31.48% | 40.67% | 36.10% |  |
| Not sure | 18.82% | 9.38% | 27.78% | 12.00% | 15.06% |  |
| **Quality of responses** |  |  |  |  |  |  |
| Very good | 20.73% | 23.96% | 28.30% | 30.20% | 26.32% | **0.078** |
| Good | 57.32% | 45.83% | 47.17% | 55.70% | 52.37% |  |
| Average | 21.95% | 30.21% | 24.53% | 14.09% | 21.32% |  |
| **Have been annoyed** |  |  |  |  |  |  |
| Yes | 26.83% | 29.17% | 32.08% | 22.15% | 26.32% | 0.448 |
| No | 73.17% | 70.83% | 67.92% | 77.85% | 73.68% |  |
| **Failed to the rationality test** |  |  |  |  |  |  |
| Yes | 0.00% | 24.00% | 50.00% | 1.20% | 13.68% | **<.001** |
| No | 100.00% | 76.00% | 50.00% | 98.80% | 86.32% |  |
| **Failed to the temporal consistency test** |  |  |  |  |  |  |
| Yes | 45.74% | 29.00% | 48.44% | 26.51% | 34.67% | **0.001** |
| No | 54.26% | 71.00% | 51.56% | 73.49% | 65.33% |  |
|  |  |  |  |  |  |  |

^1^The $p$-values refer to tests between classes using one-way analysis of variance, Kruskal-Wallis H test, Bartlett’s test for equality of variances, Fisher's exact test, and Chi2 test of independence.

# **Appendix 12 – Sociodemographic characteristics, respondent’s personal ranking of attributes and treatment modalities, and discrete choice experiment perception by cluster derived from the Hierarchical Bayesian model (**$\boldsymbol{c=4}$ **and** $\boldsymbol{c=6}$**) and full sample**

| **Sociodemographic characteristics** | **Cluster 1** | **Cluster 2** | **Cluster 3** | **Cluster 4** | **Total** | **P-value**^1^ |
| --- | --- | --- | --- | --- | --- | --- |
|  |  |  |  |  |  |  |
| **Observations** |  |  |  |  |  |  |
| n | 155 | 74 | 111 | 84 | 424 | - |
| Cluster share | 36.56% | 17.45% | 26.18% | 19.81% | 100.00% |  |
| **Gender** |  |  |  |  |  |  |
| Male | 12.26% | 18.92% | 23.42% | 27.38% | 19.34% | **0.006** |
| Female | 87.74% | 81.08% | 76.58% | 70.24% | 80.19% |  |
| Intersex | 0.00% | 0.00% | 0.00% | 2.38% | 0.47% |  |
|  |  |  |  |  |  |  |
| Female/Male Ratio | 7.16 | 4.29 | 3.27 | 2.57 | 4.15 | - |
| **Age (years)** |  |  |  |  |  |  |
| Mean | 52.95 | 53.53 | 57.34 | 58.87 | 55.38 | **0.001** |
| Standard Deviation | 12.61 | 11.79 | 11.14 | 14.07 | 12.62 |  |
| Range | (20-87) | (28-77) | (26-83) | (25-85) | (20-87) |  |
|  |  |  |  |  |  |  |
| Less than 35 | 5.81% | 6.76% | 2.70% | 4.76% | 4.95% | **0.018** |
| 35-39 | 7.10% | 6.76% | 2.70% | 7.14% | 5.90% |  |
| 40-44 | 16.77% | 13.51% | 9.91% | 7.14% | 12.50% |  |
| 45-49 | 10.32% | 8.11% | 5.41% | 5.95% | 7.78% |  |
| 50-54 | 14.19% | 13.51% | 15.32% | 11.90% | 13.92% |  |
| 55-59 | 16.77% | 13.51% | 18.02% | 9.52% | 15.09% |  |
| 60-64 | 12.26% | 13.51% | 12.61% | 14.29% | 12.97% |  |
| 65-69 | 8.39% | 17.57% | 19.82% | 10.71% | 13.44% |  |
| 70-74 | 3.23% | 5.41% | 9.91% | 16.67% | 8.02% |  |
| 75 or more | 5.16% | 1.35% | 3.60% | 11.90% | 5.42% |  |
| **Body Mass Index (BMI)** |  |  |  |  |  |  |
| Mean | 29.22 | 29.55 | 29.43 | 28.95 | 29.28 | 0.942 |
| Standard Deviation | 6.982 | 6.704 | 5.976 | 6.677 | 6.601 |  |
| Range | (13.71-62.75) | (18.42-54.69) | (19.27-58.37) | (18.42-49.94) | (13.71-62.75) |  |
| **Marital status** |  |  |  |  |  |  |
| Married | 32.26% | 37.84% | 40.54% | 35.71% | 36.08% | 0.730 |
| Living with a partner | 22.58% | 29.73% | 19.82% | 26.19% | 23.82% |  |
| Single | 21.94% | 13.51% | 13.51% | 15.48% | 16.98% |  |
| Separated | 8.39% | 4.05% | 6.31% | 4.76% | 6.37% |  |
| Divorced | 10.97% | 13.51% | 16.22% | 13.10% | 13.21% |  |
| Widowed | 3.87% | 1.35% | 3.60% | 4.76% | 3.54% |  |
| **Occupational status** |  |  |  |  |  |  |
| Employed | 26.45% | 21.62% | 16.22% | 11.90% | 20.05% | **0.006** |
| Self-employed | 7.10% | 2.70% | 2.70% | 9.52% | 5.66% |  |
| Retired | 29.03% | 35.14% | 41.44% | 45.24% | 36.56% |  |
| At home | 3.23% | 6.76% | 6.31% | 4.76% | 4.95% |  |
| Student | 4.52% | 0.00% | 0.00% | 3.57% | 2.36% |  |
| Unemployed | 0.65% | 2.70% | 4.50% | 4.76% | 2.83% |  |
| Sick leave | 12.90% | 16.22% | 10.81% | 14.29% | 13.21% |  |
| Parental leave | 0.00% | 0.00% | 0.90% | 0.00% | 0.24% |  |
| Other (e.g., disability) | 16.13% | 14.86% | 17.12% | 5.95% | 14.15% |  |
| **Educational level** |  |  |  |  |  |  |
| Secondary or less and Diploma of professional studies | 24.52% | 25.68% | 27.93% | 32.14% | 27.12% | **0.088** |
| College and CEGEP | 22.58% | 29.73% | 35.14% | 28.57% | 28.30% |  |
| Baccalaureate, Master and PhD | 52.26% | 40.54% | 36.04% | 39.29% | 43.40% |  |
| Other | 0.65% | 4.05% | 0.90% | 0.00% | 1.18% |  |
| **Annual household income (CAD)** |  |  |  |  |  |  |
| Mean | 54,516 | 63,480 | 57,860 | 59,196 | 57,883 | 0.205 |
| Standard Deviation | 39,199 | 38,614 | 37,319 | 33,961 | 37,619 |  |
| Range | (2,500-165,000) | (7,500-165,000) | (12,500-165,000) | (7,500-165,000) | (12,500-165,000) |  |
| **Living with an adult** |  |  |  |  |  |  |
| Yes | 61.29% | 70.27% | 67.57% | 69.05% | 66.04% | 0.457 |
| No | 38.71% | 29.73% | 32.43% | 30.95% | 33.96% |  |
| **Under-age dependent children (at least one)** |  |  |  |  |  |  |
| Yes | 20.00% | 18.92% | 9.91% | 13.10% | 15.80% | 0.114 |
| No | 80.00% | 81.08% | 90.09% | 86.90% | 84.20% |  |
| **Type of residence** |  |  |  |  |  |  |
| Rural | 24.52% | 27.03% | 40.54% | 23.81% | 29.01% | **0.019** |
| Urban | 75.48% | 72.97% | 59.46% | 76.19% | 70.99% |  |
| **Owning a home** |  |  |  |  |  |  |
| Yes | 60.65% | 70.27% | 72.97% | 55.95% | 64.62% | **0.041** |
| No | 39.35% | 29.73% | 27.03% | 44.05% | 35.38% |  |
| **Smoking** |  |  |  |  |  |  |
| Yes | 12.26% | 13.51% | 12.61% | 15.48% | 13.21% | 0.910 |
| No | 87.74% | 86.49% | 87.39% | 84.52% | 86.79% |  |
| **Diagnosis given by a medical doctor** |  |  |  |  |  |  |
| No diagnosis | 4.52% | 1.35% | 4.50% | 5.95% | 4.25% | 0.536 |
| Muscle and/or ligament sprain | 5.81% | 8.11% | 11.71% | 13.10% | 9.20% | 0.203 |
| Sciatica | 21.29% | 27.03% | 31.53% | 28.57% | 26.42% | 0.283 |
| Lumbar disc herniation | 33.55% | 39.19% | 44.14% | 35.71% | 37.74% | 0.346 |
| Degenerative disc disease | 19.35% | 14.86% | 18.92% | 14.29% | 17.45% | 0.686 |
| Facet arthritis | 32.26% | 45.95% | 34.23% | 30.95% | 34.91% | 0.168 |
| Vertebral arthritis or spondylarthrosis | 10.32% | 14.86% | 18.92% | 14.29% | 14.15% | 0.264 |
| Spondylolisthesis | 6.45% | 8.11% | 5.41% | 5.95% | 6.37% | 0.907 |
| Deformation (e.g., scoliosis, kyphosis) | 11.61% | 10.81% | 8.11% | 10.71% | 10.38% | 0.827 |
| Osteoporosis | 10.32% | 4.05% | 5.41% | 7.14% | 7.31% | 0.280 |
| Osteoporosis with spinal fracture | 0.00% | 1.35% | 1.80% | 1.19% | 0.94% | 0.273 |
| Fracture or dislocation of the spine | 0.65% | 2.70% | 3.60% | 2.38% | 2.12% | 0.303 |
| Autoimmune inflammatory disease | 4.52% | 5.41% | 2.70% | 4.76% | 4.25% | 0.768 |
| Fibromyalgia | 35.48% | 40.54% | 33.33% | 32.14% | 35.14% | 0.695 |
| Other diagnosis | 25.81% | 24.32% | 27.93% | 21.43% | 25.24% | 0.770 |
| **How long are you suffering from low back pain?** |  |  |  |  |  |  |
| Between 3 months and 1 year | 3.23% | 2.70% | 0.00% | 1.19% | 1.89% | 0.201 |
| More than 1 year | 96.77% | 97.30% | 100.00% | 98.81% | 98.11% |  |
| **Today low back pain** |  |  |  |  |  |  |
| Mean | 5.27 | 5.64 | 5.12 | 5.77 | 5.39 | **0.082** |
| Standard Deviation | 1.90 | 1.83 | 2.23 | 2.12 | 2.03 |  |
| Range | (1-10) | (2-10) | (0-10) | (1-10) | (0-10) |  |
| **Worst level of low back pain in the past two weeks** |  |  |  |  |  |  |
| Mean | 7.54 | 7.54 | 7.44 | 7.64 | 7.53 | 0.905 |
| Standard Deviation | 1.66 | 1.74 | 2.19 | 1.84 | 1.85 |  |
| Range | (4-10) | (4-10) | (0-10) | (2-10) | (0-10) |  |
| **Average level of low back pain in the past two weeks** |  |  |  |  |  |  |
| Mean | 5.40 | 5.60 | 5.40 | 5.91 | 5.53 | 0.192 |
| Standard Deviation | 1.78 | 1.63 | 2.02 | 2.04 | 1.88 |  |
| Range | (1-10) | (2-10) | (0-10) | (1-10) | (0-10) |  |
| **Frequency of use of painkillers** |  |  |  |  |  |  |
| Several times a day | 26.45% | 27.03% | 30.63% | 36.90% | 29.72% | 0.233 |
| Every day | 32.90% | 28.38% | 28.83% | 33.33% | 31.13% |  |
| Several times a week | 16.13% | 22.97% | 9.91% | 9.52% | 14.39% |  |
| Once a week | 1.29% | 2.70% | 2.70% | 3.57% | 2.36% |  |
| Several times a month | 9.68% | 2.70% | 6.31% | 5.95% | 6.84% |  |
| Once a month | 4.52% | 2.70% | 1.80% | 2.38% | 3.07% |  |
| Several times a year | 5.16% | 5.41% | 10.81% | 2.38% | 6.13% |  |
| Once a year | 0.00% | 1.35% | 1.80% | 0.00% | 0.71% |  |
| Never | 3.87% | 6.76% | 7.21% | 5.95% | 5.66% |  |
| **Treatments for reducing pain other than painkillers** |  |  |  |  |  |  |
| Homeopathic products | 12.26% | 4.05% | 6.31% | 2.38% | 7.31% | **0.019** |
| Infiltration of corticosteroid products | 27.10% | 55.41% | 25.23% | 50.00% | 36.08% | **<.001** |
| Chiropractic sessions | 11.61% | 18.92% | 19.82% | 5.95% | 13.92% | **0.019** |
| Physiotherapy sessions | 34.84% | 39.19% | 32.43% | 30.95% | 34.20% | 0.707 |
| Osteopathy sessions | 27.10% | 17.57% | 29.73% | 20.24% | 24.76% | 0.179 |
| Occupational therapy sessions | 5.16% | 4.05% | 5.41% | 10.71% | 6.13% | 0.319 |
| Psychotherapy sessions | 12.26% | 18.92% | 5.41% | 10.71% | 11.32% | **0.040** |
| Reflexology sessions | 3.23% | 2.70% | 2.70% | 3.57% | 3.07% | 1.000 |
| Massage therapy sessions | 44.52% | 44.59% | 36.04% | 42.86% | 41.98% | 0.521 |
| Yoga sessions | 21.94% | 12.16% | 13.51% | 10.71% | 15.80% | **0.066** |
| Stretching sessions | 22.58% | 16.22% | 13.51% | 19.05% | 18.40% | 0.281 |
| Acupuncture sessions | 13.55% | 12.16% | 10.81% | 17.86% | 13.44% | 0.537 |
| Cupping sessions | 5.16% | 1.35% | 0.90% | 1.19% | 2.59% | 0.148 |
| Infrared frequency sessions | 1.94% | 4.05% | 0.00% | 1.19% | 1.65% | 0.166 |
| Bodybuilding | 12.26% | 10.81% | 9.91% | 9.52% | 10.85% | 0.903 |
| Endurance activities (aerobic) | 9.68% | 14.86% | 6.31% | 4.76% | 8.73% | 0.105 |
| Consumption of medical cannabis | 18.06% | 13.51% | 13.51% | 16.67% | 15.80% | 0.712 |
| Others | 30.97% | 29.73% | 40.54% | 27.38% | 32.55% | 0.196 |
| **Treatment expenditure per year** |  |  |  |  |  |  |
| Mean | 1,409 | 1,589 | 1,557 | 1,727 | 1,542 | 0.629 |
| Standard Deviation | 1,648 | 2,602 | 5,236 | 3,967 | 3,519 |  |
| Range | (0-10,000) | (0-20,000) | (0-55,000) | (0-34,000) | (0-55,000) |  |
| **Insurance** |  |  |  |  |  |  |
| RAMQ (carte soleil) | 36.77% | 33.78% | 42.34% | 41.67% | 38.68% | **0.760** |
| Private insurance | 51.61% | 58.11% | 48.65% | 46.43% | 50.94% |  |
| No insurance | 11.61% | 8.11% | 9.01% | 11.90% | 10.38% |  |
| **Do you suffer from a disease or a physical or mental problem that reduces your quality of life (e.g., diabetes, cancer, osteoarthritis)?** | | | | | | |
| Yes | 65.16% | 79.73% | 63.06% | 69.05% | 67.92% | **0.089** |
| No | 34.84% | 20.27% | 36.94% | 30.95% | 32.08% |  |
|  |  |  |  |  |  |  |
| Tiredness | 75.25% | 77.97% | 74.29% | 68.97% | 74.31% | 0.723 |
| Insomnia | 55.45% | 67.80% | 54.29% | 63.79% | 59.38% | 0.308 |
| Pain | 87.13% | 96.61% | 88.57% | 84.48% | 88.89% | 0.170 |
| Anxiety/stress | 54.46% | 57.63% | 54.29% | 55.17% | 55.21% | 0.980 |
| Depression | 38.61% | 38.98% | 31.43% | 32.76% | 35.76% | 0.700 |
| Other mental disorder | 6.93% | 8.47% | 7.14% | 1.72% | 6.25% | 0.398 |
| Osteoarthritis | 65.35% | 62.71% | 67.14% | 60.34% | 64.24% | 0.861 |
| Arthritis | 16.83% | 8.47% | 18.57% | 20.69% | 16.32% | 0.290 |
| Unintentional injury | 8.91% | 1.69% | 7.14% | 3.45% | 5.90% | 0.236 |
| Musculoskeletal problem | 25.74% | 37.29% | 35.71% | 24.14% | 30.21% | 0.223 |
| Disease of the central nervous system | 9.90% | 16.95% | 7.14% | 6.90% | 10.07% | 0.222 |
| Thyroid problem | 20.79% | 15.25% | 22.86% | 13.79% | 18.75% | 0.483 |
| Other endocrin problem | 5.94% | 3.39% | 5.71% | 5.17% | 5.21% | 0.950 |
| Genital-urinary problem | 9.90% | 10.17% | 8.57% | 6.90% | 9.03% | 0.913 |
| Hypertension | 30.69% | 27.12% | 38.57% | 51.72% | 36.11% | **0.022** |
| Cardiac disease | 2.97% | 5.08% | 11.43% | 8.62% | 6.60% | 0.128 |
| Stroke | 0.99% | 1.69% | 0.00% | 1.72% | 1.04% | 0.700 |
| Digestive disorder | 28.71% | 22.03% | 30.00% | 24.14% | 26.74% | 0.694 |
| Other gastrointestinal problem | 20.79% | 20.34% | 22.86% | 17.24% | 20.49% | 0.891 |
| Diabetes | 16.83% | 15.25% | 30.00% | 29.31% | 22.22% | **0.058** |
| Cancer/tumor | 0.99% | 0.00% | 7.14% | 3.45% | 2.78% | **0.049** |
| Chronic Obstructive Pulmonary Disease (COPD) | 2.97% | 0.00% | 1.43% | 1.72% | 1.74% | 0.652 |
| Other breathing problems (asthma, emphysema) | 16.83% | 8.47% | 18.57% | 18.97% | 15.97% | 0.352 |
| Other medical disorder | 45.54% | 37.29% | 51.43% | 29.31% | 42.01% | 0.059 |
| **Health status** |  |  |  |  |  |  |
| Mean | 3.48 | 3.57 | 3.44 | 3.57 | 3.51 | 0.692 |
| Standard Deviation | 0.88 | 0.89 | 0.97 | 0.84 | 0.90 |  |
| Range | (1-5) | (1-5) | (1-5) | (2-5) | (1-5) |  |
|  |  |  |  |  |  |  |
| Excellent | 0.65% | 1.35% | 0.90% | 0.00% | 0.71% | 0.453 |
| Very good | 12.26% | 5.41% | 16.22% | 8.33% | 11.32% |  |
| Good | 36.77% | 45.95% | 36.04% | 40.48% | 38.92% |  |
| Fair | 38.71% | 29.73% | 31.53% | 36.90% | 34.91% |  |
| Poor | 11.61% | 17.57% | 15.32% | 14.29% | 14.15% |  |
| **Satisfaction with health** |  |  |  |  |  |  |
| Mean | 4.18 | 3.50 | 4.32 | 4.30 | 4.12 | 0.101 |
| Standard Deviation | 2.39 | 2.33 | 2.51 | 2.35 | 2.42 |  |
| Range | (0-10) | (0-9) | (0-9) | (0-8) | (0-10) |  |
| **Satisfaction with life** |  |  |  |  |  |  |
| Mean | 5.76 | 5.42 | 5.18 | 5.68 | 5.53 | 0.248 |
| Standard Deviation | 2.37 | 2.46 | 2.44 | 2.44 | 2.42 |  |
| Range | (0-10) | (0-10) | (0-10) | (0-10) | (0-10) |  |
| **Willingness to take risks** |  |  |  |  |  |  |
| Mean | 4.23 | 4.27 | 4.28 | 4.55 | 4.31 | 0.809 |
| Standard Deviation | 2.54 | 2.60 | 2.52 | 2.32 | 2.50 |  |
| Range | (0-9) | (0-9) | (0-10) | (0-8) | (0-10) |  |
| **Roland-Morris Disability Questionnaire^2^** |  |  |  |  |  |  |
| Mean | 9.92 | 10.21 | 10.20 | 9.77 | 10.02 | 0.936 |
| Standard Deviation | 5.46 | 4.99 | 5.55 | 4.25 | 5.18 |  |
| Range | (1-23) | (1-22) | (1-23) | (1-18) | (1-23) |  |
| **Oswestry Disability Index^3^** |  |  |  |  |  |  |
| Mean | 40.19 | 43.17 | 43.74 | 44.43 | 42.49 | 0.178 |
| Standard Deviation | 17.20 | 12.39 | 17.22 | 10.28 | 15.36 |  |
| Range | (6-92) | (12-68) | (6-86) | (24-66) | (6-92) |  |
|  |  |  |  |  |  |  |
| Minimal Disability (0-20) | 17.16% | 4.29% | 11.88% | 0.00% | 10.16% | **<.001** |
| Moderate Disability (21-40) | 36.57% | 38.57% | 29.70% | 36.23% | 35.03% |  |
| Severe Disability (41-60) | 34.33% | 47.14% | 42.57% | 59.42% | 43.58% |  |
| Crippled (61-80) | 11.19% | 10.00% | 14.85% | 4.35% | 10.70% |  |
| Bed-Bound (81-100) | 0.75% | 0.00% | 0.99% | 0.00% | 0.53% |  |
| **QALY SF-6Dv2bis^4^** |  |  |  |  |  |  |
| Mean | 0.340 | 0.297 | 0.337 | 0.342 | 0.332 | 0.593 |
| Standard Deviation | 0.238 | 0.234 | 0.228 | 0.221 | 0.231 |  |
| Range | (-0.373-0.882) | (-0.320-0.703) | (-0.277-0.843) | (-0.320-0.759) | (-0.373-0.882) |  |
| **QALY EQ-5D-5L^5^** |  |  |  |  |  |  |
| Mean | 0.593 | 0.555 | 0.579 | 0.555 | 0.575 | 0.584 |
| Standard Deviation | 0.238 | 0.208 | 0.24 | 0.216 | 0.229 |  |
| Range | (-0.072-0.904) | (0.083-0.904) | (-0.064-0.904) | (0.001-0.866) | (-0.072-0.904) |  |
| **EQ-5D-5L-VAS** |  |  |  |  |  |  |
| Mean | 55.09 | 50.41 | 51.17 | 48.91 | 52.00 | 0.226 |
| Standard deviation | 22.07 | 21.02 | 24.78 | 20.35 | 22.39 |  |
| First quartile | 40 | 34 | 31 | 31 | 34 |  |
| Median | 56 | 49 | 53 | 51.5 | 53 |  |
| Third quartile | 71 | 64 | 68 | 66 | 69 |  |
| Range | (0-100) | (0-100) | (0-100) | (12-85) | (0-100) |  |
|  |  |  |  |  |  |  |

Notes: CEGEP: Collège d’enseignement général et professionnel; RAMQ: Régie de l’assurance maladie du Québec.

^1^The $p$-values refer to tests between classes using one-way analysis of variance, Kruskal-Wallis H test, Bartlett’s test for equality of variances, Fisher's exact test, and Chi2 test of independence.

^2^The Roland-Morris Disability Questionnaire is a 24-item questionnaire measuring self-assessed back pain with a yes/no format and ranging from 0 (no back pain) to 24 (worst back pain).

^3^The Oswestry Disability Index is a 10-item questionnaire with a 6-point Likert scaling and rescaled from 0 to 100.

^4^The Short Form 6-Dimension version 2 (SF-6Dv2) is a 6-dimension generic health-related quality of life questionnaire.

^5^The EuroQol 5-Dimension 5-Level (EQ-5D-5L) is a 5-dimension generic health-related quality of life questionnaire.

| **Sociodemographic characteristics** | **Cluster 1** | **Cluster 2** | **Cluster 3** | **Cluster 4** | **Cluster 5** | **Cluster 6** | **Total** | **P-value^1^** |
| --- | --- | --- | --- | --- | --- | --- | --- | --- |
|  |  |  |  |  |  |  |  |  |
| **Observations** |  |  |  |  |  |  |  |  |
| n | 112 | 43 | 74 | 111 | 22 | 62 | 424 |  |
| Cluster share | 26.42% | 10.14% | 17.45% | 26.18% | 5.19% | 14.62% | 100.00% |  |
| **Gender** |  |  |  |  |  |  |  |  |
| Male | 15.18% | 4.65% | 18.92% | 23.42% | 31.82% | 25.81% | 19.34% | **0.001** |
| Female | 84.82% | 95.35% | 81.08% | 76.58% | 59.09% | 74.19% | 80.19% |  |
| Intersex | 0.00% | 0.00% | 0.00% | 0.00% | 9.09% | 0.00% | 0.47% |  |
|  |  |  |  |  |  |  |  |  |
| Female/Male Ratio | 5.59 | 20.50 | 4.29 | 3.27 | 1.86 | 2.88 | 4.15 | - |
|  |  |  |  |  |  |  |  |  |
| **Age (years)** |  |  |  |  |  |  |  |  |
| Mean | 52.9 | 53.09 | 53.53 | 57.34 | 61.27 | 58.02 | 55.38 | **0.003** |
| Standard Deviation | 12.36 | 13.4 | 11.79 | 11.14 | 10.88 | 15.02 | 12.62 |  |
| Range | (26-85) | (20-87) | (28-77) | (26-83) | (40-76) | (25-85) | (20-87) |  |
|  |  |  |  |  |  |  |  |  |
| Less than 35 | 6.25% | 4.65% | 6.76% | 2.70% | 0.00% | 6.45% | 4.95% | **0.084** |
| 35-39 | 8.04% | 4.65% | 6.76% | 2.70% | 0.00% | 9.68% | 5.90% |  |
| 40-44 | 15.18% | 20.93% | 13.51% | 9.91% | 4.55% | 8.06% | 12.50% |  |
| 45-49 | 9.82% | 11.63% | 8.11% | 5.41% | 13.64% | 3.23% | 7.78% |  |
| 50-54 | 14.29% | 13.95% | 13.51% | 15.32% | 18.18% | 9.68% | 13.92% |  |
| 55-59 | 18.75% | 11.63% | 13.51% | 18.02% | 4.55% | 11.29% | 15.09% |  |
| 60-64 | 10.71% | 16.28% | 13.51% | 12.61% | 13.64% | 14.52% | 12.97% |  |
| 65-69 | 8.93% | 6.98% | 17.57% | 19.82% | 13.64% | 9.68% | 13.44% |  |
| 70-74 | 3.57% | 2.33% | 5.41% | 9.91% | 22.73% | 14.52% | 8.02% |  |
| 75 or more | 4.46% | 6.98% | 1.35% | 3.60% | 9.09% | 12.90% | 5.42% |  |
| **Body Mass Index (BMI)** |  |  |  |  |  |  |  |  |
| Mean | 29.7 | 27.98 | 29.55 | 29.43 | 27.42 | 29.5 | 29.28 | 0.536 |
| Standard Deviation | 6.92 | 7.071 | 6.704 | 5.976 | 3.868 | 7.373 | 6.601 |  |
| Range | (18.00-62.75) | (13.71-49.35) | (18.42-54.69) | (19.27-58.37) | (19.07-35.26) | (18.42-49.94) | (13.71-62.75) |  |
|  |  |  |  |  |  |  |  |  |
| **Marital status** |  |  |  |  |  |  |  |  |
| Married | 32.14% | 32.56% | 37.84% | 40.54% | 40.91% | 33.87% | 36.08% | 0.859 |
| Living with a partner | 25.00% | 16.28% | 29.73% | 19.82% | 27.27% | 25.81% | 23.82% |  |
| Single | 21.43% | 23.26% | 13.51% | 13.51% | 9.09% | 17.74% | 16.98% |  |
| Separated | 8.93% | 6.98% | 4.05% | 6.31% | 4.55% | 4.84% | 6.37% |  |
| Divorced | 8.04% | 18.60% | 13.51% | 16.22% | 13.64% | 12.90% | 13.21% |  |
| Widowed | 4.46% | 2.33% | 1.35% | 3.60% | 4.55% | 4.84% | 3.54% |  |
| **Occupational status** |  |  |  |  |  |  |  |  |
| Employed | 30.36% | 16.28% | 21.62% | 16.22% | 0.00% | 16.13% | 20.05% | **0.001** |
| Self-employed | 8.04% | 4.65% | 2.70% | 2.70% | 13.64% | 8.06% | 5.66% |  |
| Retired | 25.89% | 37.21% | 35.14% | 41.44% | 59.09% | 40.32% | 36.56% |  |
| At home | 4.46% | 0.00% | 6.76% | 6.31% | 9.09% | 3.23% | 4.95% |  |
| Student | 2.68% | 9.30% | 0.00% | 0.00% | 0.00% | 4.84% | 2.36% |  |
| Unemployed | 0.00% | 2.33% | 2.70% | 4.50% | 9.09% | 3.23% | 2.83% |  |
| Sick leave | 12.50% | 13.95% | 16.22% | 10.81% | 4.55% | 17.74% | 13.21% |  |
| Parental leave | 0.00% | 0.00% | 0.00% | 0.90% | 0.00% | 0.00% | 0.24% |  |
| Other (e.g., disability) | 16.07% | 16.28% | 14.86% | 17.12% | 4.55% | 6.45% | 14.15% |  |
| **Educational level** |  |  |  |  |  |  |  |  |
| Secondary or less and Diploma of professional studies | 24.11% | 25.58% | 25.68% | 27.93% | 31.82% | 32.26% | 27.12% | **0.038** |
| College and CEGEP | 22.32% | 23.26% | 29.73% | 35.14% | 50.00% | 20.97% | 28.30% |  |
| Baccalaureate, Master and PhD | 53.57% | 48.84% | 40.54% | 36.04% | 18.18% | 46.77% | 43.40% |  |
| Other | 0.00% | 2.33% | 4.05% | 0.90% | 0.00% | 0.00% | 1.18% |  |
| **Annual household income (CAD)** |  |  |  |  |  |  |  |  |
| Mean | 57,188 | 47,558 | 63,480 | 57,860 | 45,455 | 64,073 | 57,883 | **0.089** |
| Standard Deviation | 40,590 | 34,802 | 38,614 | 37,319 | 22,034 | 36,192 | 37,619 |  |
| Range | (2,500-165,000) | (2,500-165,000) | (7,500-165,000) | (12,500-165,000) | (7,500-165,000) | (12,500-165,000) | (2,500-165,000) |  |
|  |  |  |  |  |  |  |  |  |
| **Living with an adult** |  |  |  |  |  |  |  |  |
| Yes | 61.61% | 60.47% | 70.27% | 67.57% | 68.18% | 69.35% | 66.04% | 0.757 |
| No | 38.39% | 39.53% | 29.73% | 32.43% | 31.82% | 30.65% | 33.96% |  |
| **Under-age dependent children (at least one)** | |  |  |  |  |  |  |  |
| Yes | 19.64% | 20.93% | 18.92% | 9.91% | 13.64% | 12.90% | 15.80% | 0.282 |
| No | 80.36% | 79.07% | 81.08% | 90.09% | 86.36% | 87.10% | 84.20% |  |
| **Type of residence** |  |  |  |  |  |  |  |  |
| Rural | 19.64% | 37.21% | 27.03% | 40.54% | 22.73% | 24.19% | 29.01% | **0.012** |
| Urban | 80.36% | 62.79% | 72.97% | 59.46% | 77.27% | 75.81% | 70.99% |  |
| **Owning a home** |  |  |  |  |  |  |  |  |
| Yes | 63.39% | 53.49% | 70.27% | 72.97% | 50.00% | 58.06% | 64.62% | **0.074** |
| No | 36.61% | 46.51% | 29.73% | 27.03% | 50.00% | 41.94% | 35.38% |  |
| **Smoking** |  |  |  |  |  |  |  |  |
| Yes | 10.71% | 16.28% | 13.51% | 12.61% | 18.18% | 14.52% | 13.21% | 0.860 |
| No | 89.29% | 83.72% | 86.49% | 87.39% | 81.82% | 85.48% | 86.79% |  |
| **Diagnosis given by a medical doctor** | |  |  |  |  |  |  |  |
| No diagnosis | 3.57% | 6.98% | 1.35% | 4.50% | 9.09% | 4.84% | 4.25% | 0.452 |
| Muscle and/or ligament sprain | 8.04% | 0.00% | 8.11% | 11.71% | 18.18% | 11.29% | 9.20% | **0.089** |
| Sciatica | 21.43% | 20.93% | 27.03% | 31.53% | 36.36% | 25.81% | 26.42% | 0.448 |
| Lumbar disc herniation | 33.93% | 32.56% | 39.19% | 44.14% | 40.91% | 33.87% | 37.74% | 0.597 |
| Degenerative disc disease | 17.86% | 23.26% | 14.86% | 18.92% | 9.09% | 16.13% | 17.45% | 0.784 |
| Facet arthritis | 34.82% | 25.58% | 45.95% | 34.23% | 27.27% | 32.26% | 34.91% | 0.270 |
| Vertebral arthritis or spondyloarthosis | 8.93% | 13.95% | 14.86% | 18.92% | 18.18% | 12.90% | 14.15% | 0.380 |
| Spondylolisthesis | 6.25% | 6.98% | 8.11% | 5.41% | 4.55% | 6.45% | 6.37% | 0.983 |
| Deformation (e.g., scoliosis, kyphosis) | 10.71% | 13.95% | 10.81% | 8.11% | 4.55% | 12.90% | 10.38% | 0.797 |
| Osteoporosis | 8.93% | 13.95% | 4.05% | 5.41% | 13.64% | 4.84% | 7.31% | 0.219 |
| Osteoporosis with spinal fracture | 0.00% | 0.00% | 1.35% | 1.80% | 4.55% | 0.00% | 0.94% | 0.225 |
| Fracture or dislocation of the spine | 0.00% | 2.33% | 2.70% | 3.60% | 9.09% | 0.00% | 2.12% | **0.038** |
| Autoimmune inflammatory disease | 5.36% | 2.33% | 5.41% | 2.70% | 0.00% | 6.45% | 4.25% | 0.738 |
| Fibromyalgia | 35.71% | 34.88% | 40.54% | 33.33% | 22.73% | 35.48% | 35.14% | 0.759 |
| Other diagnosis | 26.79% | 23.26% | 24.32% | 27.93% | 13.64% | 24.19% | 25.24% | 0.807 |
|  |  |  |  |  |  |  |  |  |
| **How long are you suffering from low back pain?** | |  |  |  |  |  |  |  |
| Between 3 months and 1 year | 1.79% | 6.98% | 2.70% | 0.00% | 0.00% | 1.61% | 1.89% | 0.103 |
| More than 1 year | 98.21% | 93.02% | 97.30% | 100.00% | 100.00% | 98.39% | 98.11% |  |
| **Today low back pain** |  |  |  |  |  |  |  |  |
| Mean | 5.13 | 5.63 | 5.64 | 5.12 | 6.36 | 5.57 | 5.39 | **0.048** |
| Standard Deviation | 1.91 | 1.83 | 1.83 | 2.23 | 2.11 | 2.10 | 2.03 |  |
| Range | (1-10) | (2-10) | (2-10) | (0-10) | (2-10) | (1-10) | (0-10) |  |
|  |  |  |  |  |  |  |  |  |
| **Worst level of low back pain in the past two weeks** | | |  |  |  |  |  |  |
| Mean | 7.57 | 7.44 | 7.54 | 7.44 | 7.91 | 7.55 | 7.53 | 0.932 |
| Standard Deviation | 1.66 | 1.68 | 1.74 | 2.19 | 1.85 | 1.84 | 1.85 |  |
| Range | (4-10) | (4-10) | (4-10) | (0-10) | (4-10) | (2-10) | (0-10) |  |
|  |  |  |  |  |  |  |  |  |
| **Average level of low back pain in the past two weeks** | | |  |  |  |  |  |  |
| Mean | 5.37 | 5.49 | 5.60 | 5.40 | 6.41 | 5.73 | 5.53 | 0.219 |
| Standard Deviation | 1.82 | 1.70 | 1.63 | 2.02 | 2.24 | 1.95 | 1.88 |  |
| Range | (1-10) | (2-10) | (2-10) | (0-10) | (1-10) | (1-10) | (0-10) |  |
|  |  |  |  |  |  |  |  |  |
| **Frequency of use of painkillers** |  |  |  |  |  |  |  |  |
| Several times a day | 25.89% | 27.91% | 27.03% | 30.63% | 40.91% | 35.48% | 29.72% | 0.516 |
| Every day | 30.36% | 39.53% | 28.38% | 28.83% | 36.36% | 32.26% | 31.13% |  |
| Several times a week | 18.75% | 9.30% | 22.97% | 9.91% | 9.09% | 9.68% | 14.39% |  |
| Once a week | 1.79% | 0.00% | 2.70% | 2.70% | 0.00% | 4.84% | 2.36% |  |
| Several times a month | 9.82% | 9.30% | 2.70% | 6.31% | 0.00% | 8.06% | 6.84% |  |
| Once a month | 3.57% | 6.98% | 2.70% | 1.80% | 0.00% | 3.23% | 3.07% |  |
| Several times a year | 6.25% | 2.33% | 5.41% | 10.81% | 0.00% | 3.23% | 6.13% |  |
| Once a year | 0.00% | 0.00% | 1.35% | 1.80% | 0.00% | 0.00% | 0.71% |  |
| Never | 3.57% | 4.65% | 6.76% | 7.21% | 13.64% | 3.23% | 5.66% |  |
| **Treatments for reducing pain other than painkillers** | | |  |  |  |  |  |  |
| Homeopathic products | 12.50% | 11.63% | 4.05% | 6.31% | 4.55% | 1.61% | 7.31% | **0.070** |
| Infiltration of corticosteroid products | 33.04% | 11.63% | 55.41% | 25.23% | 50.00% | 50.00% | 36.08% | **<.001** |
| Chiropractic sessions | 12.50% | 9.30% | 18.92% | 19.82% | 4.55% | 6.45% | 13.92% | **0.078** |
| Physiotherapy sessions | 36.61% | 30.23% | 39.19% | 32.43% | 31.82% | 30.65% | 34.20% | 0.854 |
| Osteopathy sessions | 28.57% | 23.26% | 17.57% | 29.73% | 18.18% | 20.97% | 24.76% | 0.364 |
| Occupational therapy sessions | 5.36% | 4.65% | 4.05% | 5.41% | 18.18% | 8.06% | 6.13% | 0.300 |
| Psychotherapy sessions | 12.50% | 11.63% | 18.92% | 5.41% | 13.64% | 9.68% | 11.32% | 0.105 |
| Reflexology sessions | 1.79% | 6.98% | 2.70% | 2.70% | 0.00% | 4.84% | 3.07% | 0.548 |
| Massage therapy sessions | 45.54% | 41.86% | 44.59% | 36.04% | 31.82% | 46.77% | 41.98% | 0.561 |
| Yoga sessions | 18.75% | 30.23% | 12.16% | 13.51% | 9.09% | 11.29% | 15.80% | 0.100 |
| Stretching sessions | 21.43% | 25.58% | 16.22% | 13.51% | 18.18% | 19.35% | 18.40% | 0.500 |
| Acupuncture sessions | 15.18% | 9.30% | 12.16% | 10.81% | 27.27% | 14.52% | 13.44% | 0.409 |
| Cupping sessions | 5.36% | 4.65% | 1.35% | 0.90% | 0.00% | 1.61% | 2.59% | 0.294 |
| Infrared frequency sessions | 2.68% | 0.00% | 4.05% | 0.00% | 0.00% | 1.61% | 1.65% | 0.266 |
| Bodybuilding | 10.71% | 16.28% | 10.81% | 9.91% | 22.73% | 4.84% | 10.85% | 0.211 |
| Endurance activities (aerobic) | 8.93% | 11.63% | 14.86% | 6.31% | 9.09% | 3.23% | 8.73% | 0.192 |
| Consumption of medical cannabis | 13.39% | 30.23% | 13.51% | 13.51% | 22.73% | 14.52% | 15.80% | 0.147 |
| Others | 34.82% | 20.93% | 29.73% | 40.54% | 4.55% | 35.48% | 32.55% | **0.013** |
|  |  |  |  |  |  |  |  |  |
| **Treatment expenditure per year (CAD)** |  |  |  |  |  |  |  |  |
| Mean | 1,484 | 1,214 | 1,589 | 1,557 | 1,277 | 1,887 | 1,542 | 0.542 |
| Standard Deviation | 1,591 | 1,792 | 2,602 | 5,236 | 1,765 | 4,499 | 3,519 |  |
| Range | (0-7,000) | (0-10,000) | (0-20,000) | (0-55,000) | (75-8,000) | (0-34,000) | (0-55,000) |  |
|  |  |  |  |  |  |  |  |  |
| **Insurance** |  |  |  |  |  |  |  |  |
| RAMQ (carte soleil) | 35.71% | 39.53% | 33.78% | 42.34% | 50.00% | 38.71% | 38.68% | 0.859 |
| Private insurance | 50.89% | 53.49% | 58.11% | 48.65% | 40.91% | 48.39% | 50.94% |  |
| No insurance | 13.39% | 6.98% | 8.11% | 9.01% | 9.09% | 12.90% | 10.38% |  |
| **Do you suffer from a disease or a physical or mental problem that reduces your quality of life (e.g., diabetes, cancer, osteoarthritis)?** | | | | | | | | |
| Yes | 67.86% | 58.14% | 79.73% | 63.06% | 50.00% | 75.81% | 67.92% | **0.025** |
| No | 32.14% | 41.86% | 20.27% | 36.94% | 50.00% | 24.19% | 32.08% |  |
|  |  |  |  |  |  |  |  |  |
| Tiredness | 76.32% | 72.00% | 77.97% | 74.29% | 81.82% | 65.96% | 74.31% | 0.771 |
| Insomnia | 55.26% | 56.00% | 67.80% | 54.29% | 81.82% | 59.57% | 59.38% | 0.375 |
| Pain | 85.53% | 92.00% | 96.61% | 88.57% | 90.91% | 82.98% | 88.89% | 0.212 |
| Anxiety/stress | 52.63% | 60.00% | 57.63% | 54.29% | 54.55% | 55.32% | 55.21% | 0.989 |
| Depression | 39.47% | 36.00% | 38.98% | 31.43% | 27.27% | 34.04% | 35.76% | 0.901 |
| Other mental disorder | 7.89% | 4.00% | 8.47% | 7.14% | 9.09% | 0.00% | 6.25% | 0.310 |
| Osteoarthritis | 67.11% | 60.00% | 62.71% | 67.14% | 54.55% | 61.70% | 64.24% | 0.917 |
| Arthritis | 17.11% | 16.00% | 8.47% | 18.57% | 18.18% | 21.28% | 16.32% | 0.503 |
| Unintentional injury | 10.53% | 4.00% | 1.69% | 7.14% | 0.00% | 4.26% | 5.90% | 0.381 |
| Musculoskeletal problem | 27.63% | 20.00% | 37.29% | 35.71% | 27.27% | 23.40% | 30.21% | 0.439 |
| Disease of the central nervous system | 10.53% | 8.00% | 16.95% | 7.14% | 0.00% | 8.51% | 10.07% | 0.509 |
| Thyroid problem | 22.37% | 16.00% | 15.25% | 22.86% | 9.09% | 14.89% | 18.75% | 0.749 |
| Other endocrin problem | 6.58% | 4.00% | 3.39% | 5.71% | 0.00% | 6.38% | 5.21% | 0.970 |
| Genital-urinary problem | 7.89% | 16.00% | 10.17% | 8.57% | 9.09% | 6.38% | 9.03% | 0.799 |
| Hypertension | 26.32% | 44.00% | 27.12% | 38.57% | 63.64% | 48.94% | 36.11% | **0.022** |
| Cardiac disease | 3.95% | 0.00% | 5.08% | 11.43% | 9.09% | 8.51% | 6.60% | 0.295 |
| Stroke | 1.32% | 0.00% | 1.69% | 0.00% | 0.00% | 2.13% | 1.04% | 0.857 |
| Digestive disorder | 31.58% | 20.00% | 22.03% | 30.00% | 27.27% | 23.40% | 26.74% | 0.749 |
| Other gastrointestinal problem | 18.42% | 28.00% | 20.34% | 22.86% | 18.18% | 17.02% | 20.49% | 0.888 |
| Diabetes | 11.84% | 32.00% | 15.25% | 30.00% | 54.55% | 23.40% | 22.22% | **0.005** |
| Cancer/tumor | 0.00% | 4.00% | 0.00% | 7.14% | 9.09% | 2.13% | 2.78% | **0.022** |
| Chronic Obstructive Pulmonary Disease (COPD) | 2.63% | 4.00% | 0.00% | 1.43% | 0.00% | 2.13% | 1.74% | 0.706 |
| Other breathing problems (asthma, emphysema) | 17.11% | 16.00% | 8.47% | 18.57% | 18.18% | 19.15% | 15.97% | 0.582 |
| Other medical disorder | 51.32% | 28.00% | 37.29% | 51.43% | 27.27% | 29.79% | 42.01% | **0.042** |
|  |  |  |  |  |  |  |  |  |
| **Health status** |  |  |  |  |  |  |  |  |
| Mean | 3.49 | 3.47 | 3.57 | 3.44 | 3.64 | 3.55 | 3.51 | 0.897 |
| Standard Deviation | 0.89 | 0.86 | 0.89 | 0.97 | 0.95 | 0.80 | 0.90 |  |
| Range | (1-5) | (2-5) | (1-5) | (1-5) | (2-5) | (2-5) | (1-5) |  |
|  |  |  |  |  |  |  |  |  |
| Excellent | 0.89% | 0.00% | 1.35% | 0.90% | 0.00% | 0.00% | 0.71% | 0.727 |
| Very good | 11.61% | 13.95% | 5.41% | 16.22% | 13.64% | 6.45% | 11.32% |  |
| Good | 37.50% | 34.88% | 45.95% | 36.04% | 27.27% | 45.16% | 38.92% |  |
| Fair | 37.50% | 41.86% | 29.73% | 31.53% | 40.91% | 35.48% | 34.91% |  |
| Poor | 12.50% | 9.30% | 17.57% | 15.32% | 18.18% | 12.90% | 14.15% |  |
| **Satisfaction with health** |  |  |  |  |  |  |  |  |
| Mean | 4.23 | 4.05 | 3.50 | 4.32 | 4.18 | 4.34 | 4.12 | 0.264 |
| Standard Deviation | 2.25 | 2.77 | 2.33 | 2.51 | 2.72 | 2.23 | 2.42 |  |
| Range | (0-10) | (0-10) | (0-9) | (0-9) | (0-8) | (0-8) | (0-10) |  |
|  |  |  |  |  |  |  |  |  |
| **Satisfaction with life** |  |  |  |  |  |  |  |  |
| Mean | 5.74 | 5.79 | 5.42 | 5.18 | 5.05 | 5.90 | 6.53 | 0.289 |
| Standard Deviation | 2.36 | 2.43 | 2.46 | 2.44 | 2.97 | 2.21 | 2.42 |  |
| Range | (0-10) | (0-10) | (0-10) | (0-10) | (0-9) | (0-10) | (0-10) |  |
|  |  |  |  |  |  |  |  |  |
| **Willingness to take risks** |  |  |  |  |  |  |  |  |
| Mean | 4.31 | 4.00 | 4.27 | 4.28 | 3.55 | 4.90 | 4.31 | 0.282 |
| Standard Deviation | 2.54 | 2.55 | 2.60 | 2.52 | 2.56 | 2.13 | 2.50 |  |
| Range | (0-9) | (0-9) | (0-9) | (0-10) | (0-8) | (0-8) | (0-10) |  |
|  |  |  |  |  |  |  |  |  |
| **Roland-Morris Disability Questionnaire^2^** | |  |  |  |  |  |  |  |
| Mean | 9.97 | 9.79 | 10.21 | 10.20 | 9.79 | 9.76 | 10.02 | 0.994 |
| Standard Deviation | 5.36 | 5.78 | 4.99 | 5.55 | 3.89 | 4.38 | 5.18 |  |
| Range | (1-23) | (1-21) | (1-22) | (1-23) | (2-16) | (1-18) | (1-23) |  |
|  |  |  |  |  |  |  |  |  |
| **Oswestry Disability Index^3^** |  |  |  |  |  |  |  |  |
| Mean | 39.4 | 42.21 | 43.17 | 43.74 | 44.53 | 44.41 | 42.49 | 0.324 |
| Standard Deviation | 17.52 | 16.44 | 12.39 | 17.22 | 7.615 | 10.97 | 15.36 |  |
| Range | (6-92) | (14-68) | (12-68) | (6-86) | (32-58) | (24-66) | (6-92) |  |
|  |  |  |  |  |  |  |  |  |
| Minimal Disability (0-20) | 15.63% | 21.05% | 4.29% | 11.88% | 0.00% | 0.00% | 10.16% | **0.001** |
| Moderate Disability (21-40) | 42.71% | 21.05% | 38.57% | 29.70% | 33.33% | 37.04% | 35.03% |  |
| Severe Disability (41-60) | 28.13% | 50.00% | 47.14% | 42.57% | 66.67% | 57.41% | 43.58% |  |
| Crippled (61-80) | 12.50% | 7.89% | 10.00% | 14.85% | 0.00% | 5.56% | 10.70% |  |
| Bed-Bound (81-100) | 1.04% | 0.00% | 0.00% | 0.99% | 0.00% | 0.00% | 0.53% |  |
| **QALY SF-6Dv2bis^4^** |  |  |  |  |  |  |  |  |
| Mean | 0.349 | 0.317 | 0.297 | 0.337 | 0.331 | 0.345 | 0.332 | 0.782 |
| Standard Deviation | 0.243 | 0.227 | 0.234 | 0.228 | 0.247 | 0.216 | 0.231 |  |
| Range | (-0.373-0.804) | (-0.259-0.882) | (-0.320-0.703) | (-0.277-0.843) | (-0.229-0.565) | (-0.320-0.759) | (-0.373-0.882) |  |
|  |  |  |  |  |  |  |  |  |
| **QALY EQ-5D-5L^5^** |  |  |  |  |  |  |  |  |
| Mean | 0.615 | 0.537 | 0.555 | 0.579 | 0.496 | 0.571 | 0.575 | 0.268 |
| Standard Deviation | 0.238 | 0.230 | 0.208 | 0.240 | 0.257 | 0.203 | 0.229 |  |
| Range | (-0.072-0.904) | (-0.044-0.885) | (0.083-0.904) | (-0.064-0.904) | (0.001-0.764) | (0.083-0.866) | (-0.072-0.904) |  |
| **EQ-5D-5L-VAS** |  |  |  |  |  |  |  |  |
| Mean | 53.83 | 58.24 | 50.41 | 51.17 | 44.33 | 50.16 | 52.00 | 0.287 |
| Standard deviation | 23.49 | 17.96 | 21.02 | 24.78 | 19.83 | 20.49 | 22.39 |  |
| First quartile | 35 | 47 | 34 | 31 | 24 | 32 | 34 |  |
| Median | 54 | 57 | 49 | 53 | 54 | 50 | 53 |  |
| Third quartile | 71 | 71 | 64 | 68 | 58 | 69 | 69 |  |
| Range | (0-100) | (15-88) | (0-100) | (0-100) | (14-70) | (12-85) | (0-100) |  |
|  |  |  |  |  |  |  |  |  |

Notes: CEGEP: Collège d’enseignement général et professionnel; RAMQ: Régie de l’assurance maladie du Québec.

^1^The $p$-values refer to tests between classes using one-way analysis of variance, Kruskal-Wallis H test, Bartlett’s test for equality of variances, Fisher's exact test, and Chi2 test of independence.

^2^The Roland-Morris Disability Questionnaire is a 24-item questionnaire measuring self-assessed back pain with a yes/no format and ranging from 0 (no back pain) to 24 (worst back pain).

^3^The Oswestry Disability Index is a 10-item questionnaire with a 6-point Likert scaling and rescaled from 0 to 100.

^4^The Short Form 6-Dimension version 2 (SF-6Dv2) is a 6-dimension generic health-related quality of life questionnaire.

^5^The EuroQol 5-Dimension 5-Level (EQ-5D-5L) is a 5-dimension generic health-related quality of life questionnaire.

| **DCE perception** | **Cluster 1** | **Cluster 2** | **Cluster 3** | **Cluster 4** | **Total** | **P-value**^1^ |
| --- | --- | --- | --- | --- | --- | --- |
|  |  |  |  |  |  |  |
| **Observations** |  |  |  |  |  |  |
| n | 155 | 74 | 111 | 84 | 424 |  |
| Cluster share | 36.56% | 17.45% | 26.18% | 19.81% | 100.00% |  |
| **Number of treatment refusals** |  |  |  |  |  |  |
| Mean | 0.57 | 0.05 | 5.71 | 4.00 | 2.51 | **<.001** |
| Standard deviation | 1.09 | 0.23 | 2.78 | 2.49 | 3.04 |  |
| Range | (0-6) | (0-1) | (1-11) | (0-11) | (0-11) |  |
| **Choice certainty score (ranging from 12 to 132)** |  |  |  |  |  |  |
| Mean | 106.00 | 100.40 | 105.30 | 102.10 | 104.00 | 0.246 |
| Standard deviation | 20.52 | 21.55 | 22.24 | 22.34 | 21.56 |  |
| Range | (24-132) | (35-132) | (13-132) | (45-132) | (13-132) |  |
| **Choice exercise difficulty** |  |  |  |  |  |  |
| Very easy | 6.57% | 4.17% | 5.77% | 2.78% | 5.19% | 0.296 |
| Easy | 29.20% | 20.83% | 22.12% | 15.28% | 23.12% |  |
| Neutral | 20.44% | 26.39% | 29.81% | 31.94% | 26.23% |  |
| Hard | 41.61% | 45.83% | 37.50% | 41.67% | 41.30% |  |
| Very hard | 2.19% | 2.78% | 4.81% | 8.33% | 4.16% |  |
| **Number of dimensions considered** |  |  |  |  |  |  |
| 1 | 1.46% | 2.78% | 1.92% | 0.00% | 1.56% | 0.026 |
| 2 | 9.49% | 15.28% | 14.42% | 9.72% | 11.95% |  |
| 3 | 34.31% | 45.83% | 26.92% | 38.89% | 35.32% |  |
| 4 or more | 42.34% | 29.17% | 39.42% | 26.39% | 36.10% |  |
| Not sure | 12.41% | 6.94% | 17.31% | 25.00% | 15.06% |  |
| **Quality of responses** |  |  |  |  |  |  |
| Very good | 30.15% | 19.44% | 32.67% | 16.90% | 26.32% | **0.073** |
| Good | 54.41% | 54.17% | 46.53% | 54.93% | 52.37% |  |
| Average | 15.44% | 26.39% | 20.79% | 28.17% | 21.32% |  |
| **Have been annoyed** |  |  |  |  |  |  |
| Yes | 24.26% | 26.39% | 27.72% | 28.17% | 26.32% | 0.914 |
| No | 75.74% | 73.61% | 72.28% | 71.83% | 73.68% |  |
| **Failed to the rationality test** |  |  |  |  |  |  |
| Yes | 0.65% | 25.68% | 1.80% | 42.86% | 13.68% | **<.001** |
| No | 99.35% | 74.32% | 98.20% | 57.14% | 86.32% |  |
|  | 100.00% | 100.00% | 100.00% | 100.00% | 100.00% |  |
| **Failed to the temporal consistency test** |  |  |  |  |  |  |
| Yes | 30.97% | 21.62% | 39.64% | 46.43% | 34.67% | **0.005** |
| No | 69.03% | 78.38% | 60.36% | 53.57% | 65.33% |  |
|  | 100.00% | 100.00% | 100.00% | 100.00% | 100.00% |  |
|  |  |  |  |  |  |  |

^1^The $p$-values refer to tests between classes using one-way analysis of variance, Kruskal-Wallis H test, Bartlett’s test for equality of variances, Fisher's exact test, and Chi2 test of independence.

| **DCE perception** | **Cluster 1** | **Cluster 2** | **Cluster 3** | **Cluster 4** | **Cluster 5** | **Cluster 6** | **Total** | **P-value^1^** |
| --- | --- | --- | --- | --- | --- | --- | --- | --- |
|  |  |  |  |  |  |  |  |  |
| **Observations** |  |  |  |  |  |  |  |  |
| n | 112 | 43 | 74 | 111 | 22 | 62 | 424 |  |
| Cluster share | 26.42% | 10.14% | 17.45% | 26.18% | 5.19% | 14.62% | 100.00% |  |
|  |  |  |  |  |  |  |  |  |
| **Number of treatment refusals** |  |  |  |  |  |  |  |  |
| Mean | 0.36 | 1.12 | 0.05 | 5.71 | 6.32 | 3.18 | 2.51 | **<.001** |
| Standard deviation | 0.75 | 1.56 | 0.23 | 2.78 | 2.97 | 1.67 | 3.04 |  |
| Range | (0-4) | (0-6) | (0-1) | (1-11) | (1-11) | (0-8) | (0-11) |  |
|  |  |  |  |  |  |  |  |  |
| **Choice certainty score (ranging from 12 to 132)** | |  |  |  |  |  |  |  |
| Mean | 105.90 | 106.30 | 100.40 | 105.30 | 104.90 | 101.40 | 104.00 | 0.484 |
| Standard deviation | 19.78 | 22.55 | 21.55 | 22.24 | 25.15 | 21.73 | 21.56 |  |
| Range | (31-132) | (24-132) | (35-132) | (13-132) | (57-132) | (45-132) | (13-132) |  |
|  |  |  |  |  |  |  |  |  |
| **Choice exercise difficulty** |  |  |  |  |  |  |  |  |
| Very easy | 3.06% | 15.38% | 4.17% | 5.77% | 6.67% | 1.75% | 5.19% | **0.059** |
| Easy | 28.57% | 30.77% | 20.83% | 22.12% | 6.67% | 17.54% | 23.12% |  |
| Neutral | 22.45% | 15.38% | 26.39% | 29.81% | 60.00% | 24.56% | 26.23% |  |
| Hard | 43.88% | 35.90% | 45.83% | 37.50% | 20.00% | 47.37% | 41.30% |  |
| Very hard | 2.04% | 2.56% | 2.78% | 4.81% | 6.67% | 8.77% | 4.16% |  |
| **Number of dimensions considered** |  |  |  |  |  |  |  |  |
| 1 | 1.02% | 2.56% | 2.78% | 1.92% | 0.00% | 0.00% | 1.56% | **0.033** |
| 2 | 7.14% | 15.38% | 15.28% | 14.42% | 0.00% | 12.28% | 11.95% |  |
| 3 | 32.65% | 38.46% | 45.83% | 26.92% | 53.33% | 35.09% | 35.32% |  |
| 4 or more | 47.96% | 28.21% | 29.17% | 39.42% | 20.00% | 28.07% | 36.10% |  |
| Not sure | 11.22% | 15.38% | 6.94% | 17.31% | 26.67% | 24.56% | 15.06% |  |
| **Quality of responses** |  |  |  |  |  |  |  |  |
| Very good | 31.96% | 25.64% | 19.44% | 32.67% | 20.00% | 16.07% | 26.32% | 0.189 |
| Good | 54.64% | 53.85% | 54.17% | 46.53% | 60.00% | 53.57% | 52.37% |  |
| Average | 13.40% | 20.51% | 26.39% | 20.79% | 20.00% | 30.36% | 21.32% |  |
| **Have been annoyed** |  |  |  |  |  |  |  |  |
| Yes | 22.68% | 28.21% | 26.39% | 27.72% | 40.00% | 25.00% | 26.32% | 0.785 |
| No | 77.32% | 71.79% | 73.61% | 72.28% | 60.00% | 75.00% | 73.68% |  |
| **Failed to the rationality test** |  |  |  |  |  |  |  |  |
| Yes | 0.00% | 2.33% | 25.68% | 1.80% | 86.36% | 27.42% | 13.68% | **<.001** |
| No | 100.00% | 97.67% | 74.32% | 98.20% | 13.64% | 72.58% | 86.32% |  |
| **Failed to the temporal consistency test** |  |  |  |  |  |  |  |  |
| Yes | 27.68% | 39.53% | 21.62% | 39.64% | 50.00% | 45.16% | 34.67% | **0.009** |
| No | 72.32% | 60.47% | 78.38% | 60.36% | 50.00% | 54.84% | 65.33% |  |
|  |  |  |  |  |  |  |  |  |

^1^The $p$-values refer to tests between classes using one-way analysis of variance, Kruskal-Wallis H test, Bartlett’s test for equality of variances, Fisher's exact test, and Chi2 test of independence.

| **Respondent's personal ranking of attributes** | **Cluster 1** | **Cluster 2** | **Cluster 3** | **Cluster 4** | **Total** | **P-value**^1^ |
| --- | --- | --- | --- | --- | --- | --- |
|  |  |  |  |  |  |  |
| **Treatment modality** |  |  |  |  |  |  |
| Mean | 4.10 | 4.68 | 4.17 | 4.86 | 4.37 | **0.034** |
| Standard deviation | 2.30 | 1.92 | 2.13 | 1.65 | 2.09 |  |
| Range | (1-7) | (1-7) | (1-7) | (1-7) | (1-7) |  |
| **Pain reduction** |  |  |  |  |  |  |
| Mean | 1.68 | 1.38 | 1.60 | 1.45 | 1.56 | 0.101 |
| Standard deviation | 1.01 | 0.74 | 0.90 | 0.95 | 0.93 |  |
| Range | (1-6) | (1-4) | (1-4) | (1-5) | (1-6) |  |
| **Onset of treatment efficacy** |  |  |  |  |  |  |
| Mean | 3.99 | 3.79 | 3.71 | 3.56 | 3.80 | 0.270 |
| Standard deviation | 1.55 | 1.65 | 1.53 | 1.62 | 1.58 |  |
| Range | (1-7) | (1-7) | (1-7) | (1-7) | (1-7) |  |
| **Effectiveness duration** |  |  |  |  |  |  |
| Mean | 3.56 | 3.38 | 3.78 | 3.28 | 3.53 | 0.109 |
| Standard deviation | 1.30 | 1.40 | 1.68 | 1.37 | 1.45 |  |
| Range | (1-7) | (1-7) | (1-7) | (1-7) | (1-7) |  |
| **Difficulties with daily activities** |  |  |  |  |  |  |
| Mean | 4.29 | 4.04 | 4.25 | 4.21 | 4.22 | 0.740 |
| Standard deviation | 1.55 | 1.59 | 1.42 | 1.63 | 1.54 |  |
| Range | (1-7) | (1-7) | (1-7) | (1-7) | (1-7) |  |
| **Sleep problems** |  |  |  |  |  |  |
| Mean | 5.14 | 5.00 | 4.97 | 4.94 | 5.03 | 0.829 |
| Standard deviation | 1.74 | 1.67 | 1.71 | 1.70 | 1.71 |  |
| Range | (1-7) | (1-7) | (1-7) | (1-7) | (1-7) |  |
| **Knowledge of his/her body and pain location** |  |  |  |  |  |  |
| Mean | 5.24 | 5.74 | 5.52 | 5.69 | 5.49 | 0.189 |
| Standard deviation | 1.94 | 1.48 | 1.90 | 1.69 | 1.81 |  |
| Range | (1-7) | (1-7) | (1-7) | (1-7) | (1-7) |  |
|  |  |  |  |  |  |  |

^1^The $p$-values refer to tests between classes using one-way analysis of variance, Kruskal-Wallis H test, Bartlett’s test for equality of variances, Fisher's exact test, and Chi2 test of independence.

| **Respondent's personal ranking of attributes** | **Cluster 1** | **Cluster 2** | **Cluster 3** | **Cluster 4** | **Cluster 5** | **Cluster 6** | **Total** | **P-value^1^** |
| --- | --- | --- | --- | --- | --- | --- | --- | --- |
|  |  |  |  |  |  |  |  |  |
| **Treatment modality** |  |  |  |  |  |  |  |  |
| Mean | 4.02 | 4.31 | 4.68 | 4.17 | 4.93 | 4.84 | 4.37 | 0.101 |
| Standard deviation | 2.27 | 2.38 | 1.92 | 2.13 | 1.44 | 1.71 | 2.09 |  |
| Range | (1-7) | (1-7) | (1-7) | (1-7) | (2-7) | (1-7) | (1-7) |  |
| **Pain reduction** |  |  |  |  |  |  |  |  |
| Mean | 1.59 | 1.90 | 1.38 | 1.60 | 1.33 | 1.48 | 1.56 | **0.085** |
| Standard deviation | 0.80 | 1.39 | 0.74 | 0.90 | 0.82 | 0.99 | 0.93 |  |
| Range | (1-4) | (1-6) | (1-4) | (1-4) | (1-4) | (1-5) | (1-6) |  |
| **Onset of treatment efficacy** |  |  |  |  |  |  |  |  |
| Mean | 3.96 | 4.08 | 3.79 | 3.71 | 4.07 | 3.43 | 3.80 | 0.305 |
| Standard deviation | 1.43 | 1.84 | 1.65 | 1.53 | 1.71 | 1.58 | 1.58 |  |
| Range | (1-7) | (2-7) | (1-7) | (1-7) | (2-7) | (1-7) | (1-7) |  |
| **Effectiveness duration** |  |  |  |  |  |  |  |  |
| Mean | 3.61 | 3.44 | 3.38 | 3.78 | 3.40 | 3.25 | 3.53 | 0.255 |
| Standard deviation | 1.34 | 1.23 | 1.40 | 1.68 | 1.64 | 1.30 | 1.45 |  |
| Range | (1-7) | (2-6) | (1-7) | (1-7) | (1-7) | (1-6) | (1-7) |  |
| **Difficulties with daily activities** |  |  |  |  |  |  |  |  |
| Mean | 4.21 | 4.49 | 4.04 | 4.25 | 4.20 | 4.21 | 4.22 | 0.824 |
| Standard deviation | 1.60 | 1.43 | 1.59 | 1.42 | 1.82 | 1.59 | 1.54 |  |
| Range | (1-7) | (2-6) | (1-7) | (1-7) | (1-7) | (1-7) | (1-7) |  |
| **Sleep problems** |  |  |  |  |  |  |  |  |
| Mean | 5.17 | 5.08 | 5.00 | 4.97 | 4.67 | 5.02 | 5.03 | 0.918 |
| Standard deviation | 1.72 | 1.81 | 1.67 | 1.71 | 1.72 | 1.70 | 1.71 |  |
| Range | (1-7) | (1-7) | (1-7) | (1-7) | (2-7) | (1-7) | (1-7) |  |
| **Knowledge of his/her body and pain location** | |  |  |  |  |  |  |  |
| Mean | 5.45 | 4.72 | 5.74 | 5.52 | 5.40 | 5.77 | 5.49 | **0.078** |
| Standard deviation | 1.88 | 2.00 | 1.48 | 1.90 | 1.96 | 1.62 | 1.81 |  |
| Range | (1-7) | (1-7) | (1-7) | (1-7) | (1-7) | (1-7) | (1-7) |  |
|  |  |  |  |  |  |  |  |  |

^1^The $p$-values refer to tests between classes using one-way analysis of variance, Kruskal-Wallis H test, Bartlett’s test for equality of variances, Fisher's exact test, and Chi2 test of independence.

| **Respondent's personal ranking of treatment modalities** | **Cluster 1** | **Cluster 2** | **Cluster 3** | **Cluster 4** | **Total** | **P-value**^1^ |
| --- | --- | --- | --- | --- | --- | --- |
|  |  |  |  |  |  |  |
| **Corticosteroid injections** |  |  |  |  |  |  |
| Mean | 4.92 | 3.28 | 4.89 | 2.66 | 4.20 | **<.001** |
| Standard deviation | 1.72 | 1.98 | 1.70 | 1.74 | 2.00 |  |
| Range | (1-6) | (1-6) | (1-6) | (1-6) | (1-6) |  |
| **Supervised body-mind physical activities** |  |  |  |  |  |  |
| Mean | 2.99 | 3.94 | 3.12 | 3.69 | 3.33 | **<.001** |
| Standard deviation | 1.46 | 1.61 | 1.41 | 1.47 | 1.52 |  |
| Range | (1-6) | (1-6) | (1-6) | (1-6) | (1-6) |  |
| **Supervised sports physical activities** |  |  |  |  |  |  |
| Mean | 3.36 | 3.84 | 3.59 | 4.12 | 3.65 | **0.013** |
| Standard deviation | 1.67 | 1.55 | 1.63 | 1.58 | 1.64 |  |
| Range | (1-6) | (1-6) | (1-6) | (1-6) | (1-6) |  |
| **Physical manipulations** |  |  |  |  |  |  |
| Mean | 2.58 | 2.57 | 2.40 | 2.93 | 2.59 | 0.188 |
| Standard deviation | 1.49 | 1.65 | 1.34 | 1.77 | 1.54 |  |
| Range | (1-6) | (1-6) | (1-6) | (1-6) | (1-6) |  |
| **Self-management courses** |  |  |  |  |  |  |
| Mean | 3.05 | 3.16 | 2.80 | 3.21 | 3.03 | 0.230 |
| Standard deviation | 1.38 | 1.43 | 1.43 | 1.44 | 1.42 |  |
| Range | (1-6) | (1-6) | (1-6) | (1-6) | (1-6) |  |
| **Psychotherapy** |  |  |  |  |  |  |
| Mean | 4.09 | 4.21 | 4.20 | 4.40 | 4.20 | 0.558 |
| Standard deviation | 1.38 | 1.49 | 1.41 | 1.58 | 1.44 |  |
| Range | (1-6) | (1-6) | (1-6) | (1-6) | (1-6) |  |
|  |  |  |  |  |  |  |

^1^The $p$-values refer to tests between classes using one-way analysis of variance, Kruskal-Wallis H test, Bartlett’s test for equality of variances, Fisher's exact test, and Chi2 test of independence.

| **Respondent's personal ranking of treatment modalities** | **Cluster 1** | **Cluster 2** | **Cluster 3** | **Cluster 4** | **Cluster 5** | **Cluster 6** | **Total** | **P-value^1^** |
| --- | --- | --- | --- | --- | --- | --- | --- | --- |
|  |  |  |  |  |  |  |  |  |
| **Corticosteroid injections** |  |  |  |  |  |  |  |  |
| Mean | 4.75 | 5.37 | 3.28 | 4.89 | 2.31 | 2.74 | 4.20 | **<.001** |
| Standard deviation | 1.81 | 1.40 | 1.98 | 1.70 | 1.97 | 1.68 | 2.00 |  |
| Range | (1-6) | (1-6) | (1-6) | (1-6) | (1-6) | (1-6) | (1-6) |  |
| **Supervised body-mind physical activities** | |  |  |  |  |  |  |  |
| Mean | 3.18 | 2.53 | 3.94 | 3.12 | 4.00 | 3.61 | 3.33 | **<.001** |
| Standard deviation | 1.43 | 1.45 | 1.61 | 1.41 | 1.41 | 1.49 | 1.52 |  |
| Range | (1-6) | (1-6) | (1-6) | (1-6) | (1-6) | (1-6) | (1-6) |  |
| **Supervised sports physical activities** |  |  |  |  |  |  |  |  |
| Mean | 3.50 | 3.00 | 3.84 | 3.59 | 4.08 | 4.13 | 3.65 | **0.020** |
| Standard deviation | 1.73 | 1.47 | 1.55 | 1.63 | 1.44 | 1.63 | 1.64 |  |
| Range | (1-6) | (1-6) | (1-6) | (1-6) | (2-6) | (1-6) | (1-6) |  |
| **Physical manipulations** |  |  |  |  |  |  |  |  |
| Mean | 2.49 | 2.82 | 2.57 | 2.40 | 3.54 | 2.78 | 2.59 | 0.127 |
| Standard deviation | 1.50 | 1.47 | 1.65 | 1.34 | 1.45 | 1.82 | 1.54 |  |
| Range | (1-6) | (1-5) | (1-6) | (1-6) | (1-6) | (1-6) | (1-6) |  |
| **Self-management courses** |  |  |  |  |  |  |  |  |
| Mean | 3.07 | 3.00 | 3.16 | 2.80 | 2.77 | 3.32 | 3.03 | 0.313 |
| Standard deviation | 1.45 | 1.21 | 1.43 | 1.43 | 1.42 | 1.44 | 1.42 |  |
| Range | (1-6) | (1-5) | (1-6) | (1-6) | (1-5) | (1-6) | (1-6) |  |
| **Psychotherapy** |  |  |  |  |  |  |  |  |
| Mean | 4.01 | 4.29 | 4.21 | 4.20 | 4.31 | 4.43 | 4.20 | 0.678 |
| Standard deviation | 1.37 | 1.39 | 1.49 | 1.41 | 1.84 | 1.53 | 1.44 |  |
| Range | (1-6) | (1-6) | (1-6) | (1-6) | (1-6) | (1-6) | (1-6) |  |
|  |  |  |  |  |  |  |  |  |

^1^The $p$-values refer to tests between classes using one-way analysis of variance, Kruskal-Wallis H test, Bartlett’s test for equality of variances, Fisher's exact test, and Chi2 test of independence.
