# Supplementary material for: Preferences of patients with chronic low back pain about nonsurgical treatments: Results of a discrete choice experiment
Source: Health Expect. 2022 Dec 8;26(1):510–30. doi: 10.1111/hex.13685 (PMC9854323; doi:10.1111/hex.13685)
Supplement: Supplementary file 2 — Supporting information. [file HEX-26--s002.docx]

**Supplementary file 1 – Multinomial logistic regression**

In order to enhance our descriptive analysis, we ran a multinomial logistic regression on classes. To do so, we included all variables that were significant in the univariate analysis ($p<0.10$) plus some variables we found of interest that were not significant. We performed several analyses on these variables. We established the correlation matrix and used a principal components analysis between the continuous variables and we explored the associations between the discrete variables (i.e., chi2 and Fisher exact tests, correspondence analysis) to assess the independency. We found that none of the variable had too high associations except the utility values of the SF-6Dv2 and the EQ-5D-5L and the values of the Roland-Morris Disability Questionnaire and the Oswestry Disability Index. However, only the Oswestry Disability Index was significantly different between classes and therefore retained.

Different specifications (e.g., continuous or discrete age) were assessed using the Bayesian information criterion.

First, we began by a complex model that included all variables. Here is the list of the variables that were included in the complex model with the initial p-value associated to the descriptive analysis (Table 2 and Appendix 4) and some comments:

- Gender ($p=0.031$): two individuals declared being “intersex” and were recoded as being “female”;
- Age (discrete) ($p=0.004$): categories were recoded (18-44, 45-54, 55-64, 64 and more);
- Marital status ($p=0.808$): categories were recoded (single, divorced, and widowed vs. married and living with a partner;
- Occupational status ($p=0.083$): categories were recoded (employed, self-employed and student vs. at home, unemployed and parental leave vs. sick leave vs. retired vs. other);
- Educational level ($p=0.046$): five individuals declared “other” educational level and were dropped from the analysis;
- Income (discrete) ($p=0.042$): the median income was considered;
- Today low back pain ($p=0.090$);
- Insurance ($p=0.091$);
- Health status (continuous) ($p=0.066$);
- Problems affecting quality of life ($p=0.138$);
- Oswestry Disability Index ($p=0.005$);
- Number of treatment refusals ($p<0.001$);
- Choice certainty score ($p=0.211$);
- Choice exercise difficulty ($p=0.124$);
- Number of dimensions considered ($p=0.004$);
- Quality of responses ($p=0.329$);
- Have been annoyed ($p=0.102$).

Because of a lack of observations or other considerations, some variables were recoded (i.e., gender, age, marital status, occupational status, and income). In particular, two individuals declared being “intersex” as gender and were recoded as being “female”.

We dropped individuals for which some variables were missing. We also dropped individuals who declared “other” as educational level ($n=5$). The full sample of the multivariate analysis was therefore $369$, or $87.03\%$ of our sample total (i.e., $369/424$).

From the complex model, we dropped variables one by one by iteration. We performed loglikelihood ratio tests and Wald tests to assess the statistical significance of variables. Some variables were not significant but were kept because of their interest (e.g., gender). At each iteration, we assessed the Hosmer-Lemeshow Goodness of Fit test for multinomial logit regression^1^.

Finally, we presented the results in terms of relative risk ratio (RRR) with class 5 being the base outcome.

Here are the details about variables we retained for the final model:

| **Respondents' characteristics** | **Class 1** | **Class 2** | **Class 3** | **Class 4** | **Class 5** | **Class 6** | **Total** | **P-value^1^** |
| --- | --- | --- | --- | --- | --- | --- | --- | --- |
|  |  |  |  |  |  |  |  |  |
| **Observations** | 44 | 47 | 37 | 62 | 139 | 40 | 369 |  |
|  | 11.92% | 12.74% | 10.03% | 16.80% | 37.67% | 10.84% |  |  |
|  |  |  |  |  |  |  |  |  |
| **Gender** |  |  |  |  |  |  |  |  |
| Male | 18.18% | 23.40% | 24.32% | 14.52% | 15.11% | 27.50% | 18.70% | 0.370 |
| Female | 81.82% | 76.60% | 75.68% | 85.48% | 84.89% | 72.50% | 81.30% |  |
|  |  |  |  |  |  |  |  |  |
| Female/Male Ratio | 4.50 | 3.27 | 3.11 | 5.89 | 5.62 | 2.64 | 4.35 | - |
| **Age** |  |  |  |  |  |  |  |  |
| 18-44 | 29.55% | 19.15% | 16.22% | 30.65% | 28.06% | 7.50% | 24.12% | 0.003 |
| 45-54 | 18.18% | 21.28% | 29.73% | 22.58% | 25.18% | 20.00% | 23.31% |  |
| 55-64 | 25.00% | 38.30% | 21.62% | 11.29% | 33.09% | 35.00% | 28.18% |  |
| 65 and more | 27.27% | 21.28% | 32.43% | 35.48% | 13.67% | 37.50% | 24.39% |  |
| **Occupational status** |  |  |  |  |  |  |  |  |
| Employed, self-employed, and student | 15.91% | 23.40% | 32.43% | 30.65% | 36.69% | 15.00% | 28.73% | 0.128 |
| At home, unemployed, and parental leave | 6.82% | 10.64% | 5.41% | 9.68% | 7.19% | 12.50% | 8.40% |  |
| Sick leave | 22.73% | 8.51% | 13.51% | 8.06% | 12.95% | 20.00% | 13.55% |  |
| Retired | 38.64% | 42.55% | 32.43% | 37.10% | 24.46% | 45.00% | 33.60% |  |
| Other (e.g., disability) | 15.91% | 14.89% | 16.22% | 14.52% | 18.71% | 7.50% | 15.72% |  |
| **Educational level** |  |  |  |  |  |  |  |  |
| Secondary or less and Diploma of professional studies | 27.27% | 23.40% | 29.73% | 25.81% | 29.50% | 37.50% | 28.73% | 0.158 |
| College and CEGEP | 29.55% | 36.17% | 35.14% | 32.26% | 18.71% | 35.00% | 27.91% |  |
| Baccalaureate, Master and PhD | 43.18% | 40.43% | 35.14% | 41.94% | 51.80% | 27.50% | 43.36% |  |
| **Median income (CAD)** |  |  |  |  |  |  |  |  |
| <52,500 | 38.64% | 34.04% | 51.35% | 56.45% | 51.80% | 45.00% | 47.97% | 0.151 |
| ≥52,500 | 61.36% | 65.96% | 48.65% | 43.55% | 48.20% | 55.00% | 52.03% |  |
| **Insurance** |  |  |  |  |  |  |  |  |
| RAMQ (carte soleil) | 36.36% | 29.79% | 40.54% | 43.55% | 33.81% | 45.00% | 37.13% | 0.069 |
| Private insurance | 63.64% | 65.96% | 45.95% | 45.16% | 52.52% | 42.50% | 52.57% |  |
| No insurance | 0.00% | 4.26% | 13.51% | 11.29% | 13.67% | 12.50% | 10.30% |  |
| **Problem affecting quality of life** |  |  |  |  |  |  |  |  |
| Yes | 22.73% | 27.66% | 16.22% | 33.87% | 36.69% | 45.00% | 32.25% | 0.053 |
| No | 77.27% | 72.34% | 83.78% | 66.13% | 63.31% | 55.00% | 67.75% |  |
| **Oswestry Disability Index^2^** |  |  |  |  |  |  |  |  |
| Mean | 44.45 | 43.57 | 46.11 | 42.65 | 38.78 | 47.65 | 42.41 | 0.007 |
| Standard Deviation | 12.21 | 16.2 | 13.67 | 15.38 | 16.39 | 12.41 | 15.32 |  |
| Range | (20-66) | (6-80) | (14-92) | (6-78) | (6-76) | (24-86) | (6-92) |  |
| **Number of treatment refusals** |  |  |  |  |  |  |  |  |
| Mean | 0.71 | 3.34 | 1.78 | 5.89 | 0.19 | 8.20 | 2.64 | 0.000 |
| Standard Deviation | 1.03 | 1.45 | 1.32 | 1.95 | 0.43 | 2.24 | 3.13 |  |
| Range | (0-3) | (1-6) | (0-4) | (2-10) | (0-2) | (4-11) | (0-11) |  |
| **Number of dimensions considered** |  |  |  |  |  |  |  |  |
| 1 or 2 | 18.18% | 21.28% | 16.22% | 8.06% | 10.79% | 10.00% | 13.01% | 0.004 |
| 3 or 4 and more | 79.55% | 59.57% | 54.05% | 75.81% | 78.42% | 65.00% | 71.82% |  |
| Don't know | 2.27% | 19.15% | 29.73% | 16.13% | 10.79% | 25.00% | 15.18% |  |
|  |  |  |  |  |  |  |  |  |

^1^The $p$-values refer to tests between classes using one-way analysis of variance, Kruskal-Wallis H test, Bartlett’s test for equality of variances, Fisher's exact test, and Chi2 test of independence.

^2^The Oswestry Disability Index is a 10-item questionnaire with a 6-point Likert scaling and rescaled from 0 to 100.

| **Multinomial logistic regression** | | | | | | | | | | | | | | | |
| --- | --- | --- | --- | --- | --- | --- | --- | --- | --- | --- | --- | --- | --- | --- | --- |
|  | Class 1 | | | Class 2 | | | Class 3 | | | Class 4 | | | Class 6 | | |
|  | RRR | Standard error | P-value | RRR | Standard error | P-value | RRR | Standard error | P-value | RRR | Standard error | P-value | RRR | Standard error | P-value |
|  |  |  |  |  |  |  |  |  |  |  |  |  |  |  |  |
| Constant | 0.018*** | 0.021 | 0.001 | <.001*** | 0.001 | <.001 | 0.003*** | 0.005 | <.001 | <.001*** | <.001 | <.001 | <.001*** | <.001 | <.001 |
|  |  |  |  |  |  |  |  |  |  |  |  |  |  |  |  |
| Gender |  |  |  |  |  |  |  |  |  |  |  |  |  |  |  |
| Male | (reference) |  |  |  |  |  |  |  |  |  |  |  |  |  |  |
| Female | 0.920 | 0.511 | 0.881 | 0.876 | 0.694 | 0.867 | 0.878 | 0.597 | 0.848 | 1.515 | 1.593 | 0.693 | 0.800 | 0.960 | 0.852 |
| Age |  |  |  |  |  |  |  |  |  |  |  |  |  |  |  |
| 18-44 | (reference) |  |  |  |  |  |  |  |  |  |  |  |  |  |  |
| 45-54 | 0.607 | 0.355 | 0.394 | 0.602 | 0.584 | 0.601 | 2.595 | 1.930 | 0.200 | 0.074* | 0.099 | 0.051 | 1.491 | 2.772 | 0.830 |
| 55-64 | 0.446 | 0.289 | 0.213 | 0.406 | 0.414 | 0.377 | 1.061 | 0.930 | 0.946 | 0.006*** | 0.009 | 0.001 | 0.109 | 0.207 | 0.243 |
| 65 and more | 0.887 | 0.810 | 0.895 | 0.717 | 0.956 | 0.803 | 7.813* | 9.380 | 0.087 | 0.150 | 0.249 | 0.254 | 1.108 | 2.420 | 0.962 |
| Occupational status |  |  |  |  |  |  |  |  |  |  |  |  |  |  |  |
| Employed, self-employed, and student | (reference) |  |  |  |  |  |  |  |  |  |  |  |  |  |  |
| At home, unemployed, and parental leave | 2.446 | 2.224 | 0.326 | 1.730 | 2.347 | 0.686 | 0.191 | 0.237 | 0.182 | 1.548 | 2.753 | 0.806 | 2.980 | 5.937 | 0.584 |
| Sick leave | 2.286 | 1.560 | 0.226 | 0.412 | 0.464 | 0.431 | 0.291 | 0.265 | 0.175 | 0.441 | 0.678 | 0.595 | 1.821 | 3.372 | 0.746 |
| Retired | 4.497* | 3.758 | 0.072 | 1.582 | 1.759 | 0.680 | 0.252 | 0.264 | 0.188 | 2.078 | 3.049 | 0.618 | 4.261 | 7.464 | 0.408 |
| Other (e.g., disability) | 2.141 | 1.589 | 0.305 | 0.567 | 0.642 | 0.616 | 0.215 | 0.201 | 0.101 | 0.734 | 0.994 | 0.820 | 0.564 | 0.933 | 0.729 |
| Educational level |  |  |  |  |  |  |  |  |  |  |  |  |  |  |  |
| Secondary or less and Diploma of professional studies | (reference) |  |  |  |  |  |  |  |  |  |  |  |  |  |  |
| College and CEGEP | 1.711 | 1.006 | 0.361 | 5.898* | 5.475 | 0.056 | 2.511 | 1.819 | 0.204 | 2.276 | 2.669 | 0.483 | 0.302 | 0.417 | 0.386 |
| Baccalaureate, Master and PhD | 0.835 | 0.458 | 0.743 | 2.049 | 1.826 | 0.421 | 0.611 | 0.421 | 0.474 | 3.514 | 3.812 | 0.247 | 0.761 | 0.943 | 0.826 |
| Median income (CAD) |  |  |  |  |  |  |  |  |  |  |  |  |  |  |  |
| <52,500 | (reference) |  |  |  |  |  |  |  |  |  |  |  |  |  |  |
| ≥52,500 | 1.278 | 0.634 | 0.621 | 1.161 | 0.879 | 0.844 | 1.540 | 0.976 | 0.496 | 0.237 | 0.238 | 0.153 | 1.118 | 1.323 | 0.925 |
| Insurance |  |  |  |  |  |  |  |  |  |  |  |  |  |  |  |
| RAMQ (carte soleil) | (reference) |  |  |  |  |  |  |  |  |  |  |  |  |  |  |
| Private insurance | 1.774 | 0.971 | 0.295 | 1.744 | 1.403 | 0.489 | 0.677 | 0.474 | 0.577 | 0.657 | 0.678 | 0.684 | 0.130 | 0.165 | 0.108 |
| No insurance | <0.001 | <.001 | 0.982 | 0.100 | 0.140 | 0.101 | 0.671 | 0.623 | 0.667 | 0.143 | 0.208 | 0.181 | 0.110 | 0.184 | 0.187 |
| Problem affecting quality of life |  |  |  |  |  |  |  |  |  |  |  |  |  |  |  |
| No | (reference) |  |  |  |  |  |  |  |  |  |  |  |  |  |  |
| Yes | 2.033 | 0.965 | 0.135 | 1.668 | 1.216 | 0.482 | 4.218** | 2.760 | 0.028 | 0.935 | 0.844 | 0.940 | 0.351 | 0.371 | 0.322 |
| Oswestry Disability Index |  |  |  |  |  |  |  |  |  |  |  |  |  |  |  |
|  | 1.034** | 0.015 | 0.024 | 1.056** | 0.027 | 0.032 | 1.066*** | 0.022 | 0.002 | 1.056* | 0.033 | 0.082 | 1.082** | 0.038 | 0.027 |
| Number of treatment refusals |  |  |  |  |  |  |  |  |  |  |  |  |  |  |  |
|  | 3.604*** | 1.096 | <.001 | 23.964*** | 9.524 | <.001 | 7.535*** | 2.435 | <.001 | 96.589*** | 47.862 | <.001 | 198.497*** | 104.070 | <.001 |
| Number of dimensions considered |  |  |  |  |  |  |  |  |  |  |  |  |  |  |  |
| 1 or 2 | (reference) |  |  |  |  |  |  |  |  |  |  |  |  |  |  |
| 3 or 4 and more | 0.812 | 0.474 | 0.721 | 0.491 | 0.426 | 0.413 | 0.446 | 0.326 | 0.270 | 6.889 | 10.017 | 0.184 | 16.477 | 28.481 | 0.105 |
| Don't know | 0.087 | 0.110 | 0.053 | 0.182 | 0.215 | 0.150 | 0.656 | 0.672 | 0.681 | 0.574 | 0.954 | 0.738 | 0.864 | 1.655 | 0.939 |
|  |  |  |  |  |  |  |  |  |  |  |  |  |  |  |  |
| Observations | 369 |  |  |  |  |  |  |  |  |  |  |  |  |  |  |
| McFadden's R2 | 0.563 |  |  |  |  |  |  |  |  |  |  |  |  |  |  |
| Chi2 | 687.697 |  |  |  |  |  |  |  |  |  |  |  |  |  |  |
| Log likelihood | -266.841 |  |  |  |  |  |  |  |  |  |  |  |  |  |  |
| Null Log likelihood | -610.689 |  |  |  |  |  |  |  |  |  |  |  |  |  |  |
| AIC | 723.681 |  |  |  |  |  |  |  |  |  |  |  |  |  |  |
| BIC | 1,095.207 |  |  |  |  |  |  |  |  |  |  |  |  |  |  |

Notes: class 5 is the base outcome; RRR: relative risk ratio; AIC: Akaike Information Criterion; BIC: Bayesian Information Criterion; RAMQ: Régime d’Assurance Maladie du Québec.

**References**

1. Fagerland MW, Hosmer DW. A Generalized Hosmer–Lemeshow Goodness-of-Fit Test for Multinomial Logistic Regression Models. *The Stata Journal*. 2012;12(3):447-453. doi:10.1177/1536867X1201200307
