# Supplementary material for: Preferences of patients with chronic low back pain about nonsurgical treatments: Results of a discrete choice experiment
Source: Health Expect. 2022 Dec 8;26(1):510–30. doi: 10.1111/hex.13685 (PMC9854323; doi:10.1111/hex.13685)
Supplement: Supplementary file 3 — Supporting information. [file HEX-26--s003.docx]

**Supplementary file 2 – Four-item Low Back Pain Treatment Beliefs Questionnaire by latent class (details)**^1,2^

| **Four-item Low Back Pain Treatment Beliefs Questionnaire (details)** | **Class 1** | **Class 2** | **Class 3** | **Class 4** | **Total** | **P-value**^1^ |
| --- | --- | --- | --- | --- | --- | --- |
|  |  |  |  |  |  |  |
| **Observations** | 80 | 89 | 48 | 143 | 360 |  |
|  | 22.22% | 24.72% | 13.33% | 39.72% | 100.00% | - |
|  |  |  |  |  |  |  |
| **Treatment 1 - Corticosteroid injections** | | | | | | |
|  |  |  |  |  |  |  |
| **Taking/Having this treatment for back pain makes a lot of sense (1-5)** | |  |  |  |  |  |
| Strongly disagree | 15.00% | 4.49% | 25.00% | 28.67% | 19.17% | **<.001** |
| Disagree | 12.50% | 5.62% | 8.33% | 24.48% | 15.00% |  |
| Neither agree nor disagree | 31.25% | 22.47% | 18.75% | 25.87% | 25.28% |  |
| Agree | 32.50% | 39.33% | 33.33% | 13.99% | 26.94% |  |
| Strongly agree | 8.75% | 28.09% | 14.58% | 6.99% | 13.61% |  |
|  |  |  |  |  |  |  |
| Mean | 3.08 | 3.81 | 3.04 | 2.46 | 3.01 | **<.001** |
| Standard deviation | 1.19 | 1.05 | 1.43 | 1.24 | 1.32 |  |
| Range | (1-5) | (1-5) | (1-5) | (1-5) | (1-5) |  |
| **I think this treatment is pretty useless for people with back pain (1-5)*** | |  |  |  |  |  |
| Strongly disagree | 17.50% | 33.71% | 29.17% | 18.88% | 23.61% | **0.006** |
| Disagree | 28.75% | 31.46% | 16.67% | 20.28% | 24.44% |  |
| Neither agree nor disagree | 35.00% | 14.61% | 25.00% | 28.67% | 26.11% |  |
| Agree | 8.75% | 15.73% | 16.67% | 16.08% | 14.44% |  |
| Strongly agree | 10.00% | 4.49% | 12.50% | 16.08% | 11.39% |  |
|  |  |  |  |  |  |  |
| Mean | 2.65 | 2.26 | 2.67 | 2.90 | 2.66 | **0.003** |
| Standard deviation | 1.17 | 1.21 | 1.39 | 1.33 | 1.29 |  |
| Range | (1-5) | (1-5) | (1-5) | (1-5) | (1-5) |  |
| **I have concerns about taking/having this treatment for my back pain (1-5)*** | |  |  |  |  |  |
| Strongly disagree | 10.00% | 21.35% | 29.17% | 10.49% | 15.56% | **<.001** |
| Disagree | 18.75% | 24.72% | 16.67% | 6.99% | 15.28% |  |
| Neither agree nor disagree | 17.50% | 17.98% | 20.83% | 11.89% | 15.83% |  |
| Agree | 23.75% | 28.09% | 12.50% | 34.27% | 27.50% |  |
| Strongly agree | 30.00% | 7.87% | 20.83% | 36.36% | 25.83% |  |
|  |  |  |  |  |  |  |
| Mean | 3.45 | 2.76 | 2.79 | 3.79 | 3.33 | **<.001** |
| Standard deviation | 1.36 | 1.29 | 1.52 | 1.29 | 1.41 |  |
| Range | (1-5) | (1-5) | (1-5) | (1-5) | (1-5) |  |
| **I am confident this treatment would be a suitable treatment for my back pain (1-5)** | | |  |  |  |  |
| Strongly disagree | 25.00% | 3.37% | 27.08% | 37.06% | 24.72% | **<.001** |
| Disagree | 7.50% | 4.49% | 14.58% | 16.78% | 11.39% |  |
| Neither agree nor disagree | 30.00% | 25.84% | 16.67% | 26.57% | 25.83% |  |
| Agree | 26.25% | 34.83% | 27.08% | 11.89% | 22.78% |  |
| Strongly agree | 11.25% | 31.46% | 14.58% | 7.69% | 15.28% |  |
|  |  |  |  |  |  |  |
| Mean | 2.91 | 3.87 | 2.88 | 2.36 | 2.93 | **<.001** |
| Standard deviation | 1.34 | 1.02 | 1.45 | 1.30 | 1.39 |  |
| Range | (1-5) | (1-5) | (1-5) | (1-5) | (1-5) |  |
|  |  |  |  |  |  |  |
| **Treatment 2 - Supervised body-mind physical activities** | | | | | | |
|  |  |  |  |  |  |  |
| **Taking/Having this treatment for back pain makes a lot of sense (1-5)** | |  |  |  |  |  |
| Strongly disagree | 1.25% | 3.37% | 8.33% | 2.10% | 3.06% | **<.001** |
| Disagree | 6.25% | 5.62% | 6.25% | 2.80% | 4.72% |  |
| Neither agree nor disagree | 32.50% | 28.09% | 29.17% | 9.79% | 21.94% |  |
| Agree | 40.00% | 40.45% | 39.58% | 40.56% | 40.28% |  |
| Strongly agree | 20.00% | 22.47% | 16.67% | 44.76% | 30.00% |  |
|  |  |  |  |  |  |  |
| Mean | 3.71 | 3.73 | 3.50 | 4.23 | 3.89 | **<.001** |
| Standard deviation | 0.90 | 0.99 | 1.11 | 0.89 | 0.99 |  |
| Range | (1-5) | (1-5) | (1-5) | (1-5) | (1-5) |  |
| **I think this treatment is pretty useless for people with back pain (1-5)*** | |  |  |  |  |  |
| Strongly disagree | 20.00% | 24.72% | 18.75% | 34.27% | 26.67% | **0.058** |
| Disagree | 40.00% | 26.97% | 27.08% | 30.77% | 31.39% |  |
| Neither agree nor disagree | 23.75% | 23.60% | 27.08% | 11.89% | 19.44% |  |
| Agree | 12.50% | 19.10% | 20.83% | 13.99% | 15.83% |  |
| Strongly agree | 3.75% | 5.62% | 6.25% | 9.09% | 6.67% |  |
|  |  |  |  |  |  |  |
| Mean | 2.40 | 2.54 | 2.69 | 2.33 | 2.44 | 0.284 |
| Standard deviation | 1.06 | 1.22 | 1.19 | 1.32 | 1.23 |  |
| Range | (1-5) | (1-5) | (1-5) | (1-5) | (1-5) |  |
| **I have concerns about taking/having this treatment for my back pain (1-5)*** | |  |  |  |  |  |
| Strongly disagree | 23.75% | 29.21% | 22.92% | 37.76% | 30.56% | **0.022** |
| Disagree | 35.00% | 30.34% | 29.17% | 35.66% | 33.33% |  |
| Neither agree nor disagree | 30.00% | 23.60% | 20.83% | 20.28% | 23.33% |  |
| Agree | 7.50% | 13.48% | 14.58% | 4.90% | 8.89% |  |
| Strongly agree | 3.75% | 3.37% | 12.50% | 1.40% | 3.89% |  |
|  |  |  |  |  |  |  |
| Mean | 2.33 | 2.31 | 2.65 | 1.97 | 2.22 | **0.001** |
| Standard deviation | 1.04 | 1.13 | 1.33 | 0.95 | 1.09 |  |
| Range | (1-5) | (1-5) | (1-5) | (1-5) | (1-5) |  |
| **I am confident this treatment would be a suitable treatment for my back pain (1-5)** | | |  |  |  |  |
| Strongly disagree | 7.50% | 3.37% | 8.33% | 2.80% | 4.72% | **<.001** |
| Disagree | 7.50% | 5.62% | 12.50% | 0.70% | 5.00% |  |
| Neither agree nor disagree | 22.50% | 25.84% | 29.17% | 11.89% | 20.00% |  |
| Agree | 43.75% | 41.57% | 39.58% | 48.25% | 44.44% |  |
| Strongly agree | 18.75% | 23.60% | 10.42% | 36.36% | 25.83% |  |
|  |  |  |  |  |  |  |
| Mean | 3.59 | 3.76 | 3.31 | 4.15 | 3.82 | **<.001** |
| Standard deviation | 1.11 | 0.99 | 1.09 | 0.86 | 1.03 |  |
| Range | (1-5) | (1-5) | (1-5) | (1-5) | (1-5) |  |
|  |  |  |  |  |  |  |
| **Treatment 3 - Supervised sports-type physical activities** | | | | | | |
|  |  |  |  |  |  |  |
| **Taking/Having this treatment for back pain makes a lot of sense (1-5)** | |  |  |  |  |  |
| Strongly disagree | 6.25% | 4.49% | 14.58% | 4.20% | 6.11% | **0.001** |
| Disagree | 11.25% | 7.87% | 10.42% | 6.99% | 8.61% |  |
| Neither agree nor disagree | 28.75% | 29.21% | 29.17% | 14.69% | 23.33% |  |
| Agree | 35.00% | 41.57% | 37.50% | 39.16% | 38.61% |  |
| Strongly agree | 18.75% | 16.85% | 8.33% | 34.97% | 23.33% |  |
|  |  |  |  |  |  |  |
| Mean | 3.49 | 3.58 | 3.15 | 3.94 | 3.64 | **<.001** |
| Standard deviation | 1.11 | 1.01 | 1.18 | 1.08 | 1.11 |  |
| Range | (1-5) | (1-5) | (1-5) | (1-5) | (1-5) |  |
| **I think this treatment is pretty useless for people with back pain (1-5)*** | |  |  |  |  |  |
| Strongly disagree | 27.50% | 22.47% | 10.42% | 33.57% | 26.39% | **0.009** |
| Disagree | 27.50% | 28.09% | 27.08% | 32.17% | 29.44% |  |
| Neither agree nor disagree | 27.50% | 28.09% | 33.33% | 11.89% | 22.22% |  |
| Agree | 12.50% | 14.61% | 25.00% | 14.69% | 15.56% |  |
| Strongly agree | 5.00% | 6.74% | 4.17% | 7.69% | 6.39% |  |
|  |  |  |  |  |  |  |
| Mean | 2.40 | 2.55 | 2.85 | 2.31 | 2.46 | **0.045** |
| Standard deviation | 1.16 | 1.19 | 1.05 | 1.28 | 1.21 |  |
| Range | (1-5) | (1-5) | (1-5) | (1-5) | (1-5) |  |
| **I have concerns about taking/having this treatment for my back pain (1-5)*** | |  |  |  |  |  |
| Strongly disagree | 20.00% | 28.09% | 10.42% | 27.27% | 23.61% | **0.029** |
| Disagree | 28.75% | 20.22% | 25.00% | 34.27% | 28.33% |  |
| Neither agree nor disagree | 13.75% | 24.72% | 27.08% | 15.38% | 18.89% |  |
| Agree | 27.50% | 20.22% | 20.83% | 15.38% | 20.00% |  |
| Strongly agree | 10.00% | 6.74% | 16.67% | 7.69% | 9.17% |  |
|  |  |  |  |  |  |  |
| Mean | 2.79 | 2.57 | 3.08 | 2.42 | 2.63 | **0.011** |
| Standard deviation | 1.32 | 1.28 | 1.25 | 1.25 | 1.29 |  |
| Range | (1-5) | (1-5) | (1-5) | (1-5) | (1-5) |  |
| **I am confident this treatment would be a suitable treatment for my back pain (1-5)** | | |  |  |  |  |
| Strongly disagree | 13.75% | 4.49% | 14.58% | 6.29% | 8.61% | **<.001** |
| Disagree | 16.25% | 12.36% | 10.42% | 9.09% | 11.67% |  |
| Neither agree nor disagree | 18.75% | 21.35% | 39.58% | 11.19% | 19.17% |  |
| Agree | 35.00% | 43.82% | 25.00% | 41.26% | 38.33% |  |
| Strongly agree | 16.25% | 17.98% | 10.42% | 32.17% | 22.22% |  |
|  |  |  |  |  |  |  |
| Mean | 3.24 | 3.58 | 3.06 | 3.84 | 3.54 | **<.001** |
| Standard deviation | 1.30 | 1.06 | 1.17 | 1.16 | 1.20 |  |
| Range | (1-5) | (1-5) | (1-5) | (1-5) | (1-5) |  |
|  |  |  |  |  |  |  |
| **Treatment 4 - Physical manipulations** | | | | | | |
|  |  |  |  |  |  |  |
| **Taking/Having this treatment for back pain makes a lot of sense (1-5)** | |  |  |  |  |  |
| Strongly disagree | 0.00% | 4.49% | 10.42% | 0.70% | 2.78% | **<.001** |
| Disagree | 1.25% | 4.49% | 8.33% | 1.40% | 3.06% |  |
| Neither agree nor disagree | 6.25% | 13.48% | 25.00% | 12.59% | 13.06% |  |
| Agree | 46.25% | 39.33% | 43.75% | 30.77% | 38.06% |  |
| Strongly agree | 46.25% | 38.20% | 12.50% | 54.55% | 43.06% |  |
|  |  |  |  |  |  |  |
| Mean | 4.38 | 4.02 | 3.40 | 4.37 | 4.16 | **<.001** |
| Standard deviation | 0.66 | 1.06 | 1.14 | 0.81 | 0.95 |  |
| Range | (2-5) | (1-5) | (1-5) | (1-5) | (1-5) |  |
| **I think this treatment is pretty useless for people with back pain (1-5)*** | |  |  |  |  |  |
| Strongly disagree | 43.75% | 38.20% | 14.58% | 41.96% | 37.78% | **<.001** |
| Disagree | 27.50% | 22.47% | 16.67% | 25.17% | 23.89% |  |
| Neither agree nor disagree | 12.50% | 14.61% | 47.92% | 12.59% | 17.78% |  |
| Agree | 12.50% | 17.98% | 14.58% | 9.09% | 12.78% |  |
| Strongly agree | 3.75% | 6.74% | 6.25% | 11.19% | 7.78% |  |
|  |  |  |  |  |  |  |
| Mean | 2.05 | 2.33 | 2.81 | 2.22 | 2.29 | **0.012** |
| Standard deviation | 1.19 | 1.33 | 1.07 | 1.37 | 1.30 |  |
| Range | (1-5) | (1-5) | (1-5) | (1-5) | (1-5) |  |
| **I have concerns about taking/having this treatment for my back pain (1-5)*** | |  |  |  |  |  |
| Strongly disagree | 38.75% | 30.34% | 12.50% | 39.86% | 33.61% | **<.001** |
| Disagree | 36.25% | 30.34% | 27.08% | 32.87% | 32.22% |  |
| Neither agree nor disagree | 17.50% | 20.22% | 27.08% | 17.48% | 19.44% |  |
| Agree | 6.25% | 16.85% | 14.58% | 7.69% | 10.56% |  |
| Strongly agree | 1.25% | 2.25% | 18.75% | 2.10% | 4.17% |  |
|  |  |  |  |  |  |  |
| Mean | 1.95 | 2.30 | 3.00 | 1.99 | 2.19 | **<.001** |
| Standard deviation | 0.97 | 1.14 | 1.30 | 1.04 | 1.14 |  |
| Range | (1-5) | (1-5) | (1-5) | (1-5) | (1-5) |  |
| **I am confident this treatment would be a suitable treatment for my back pain (1-5)** | | |  |  |  |  |
| Strongly disagree | 0.00% | 3.37% | 10.42% | 1.40% | 2.78% | **<.001** |
| Disagree | 1.25% | 7.87% | 18.75% | 3.50% | 6.11% |  |
| Neither agree nor disagree | 8.75% | 7.87% | 27.08% | 13.29% | 12.78% |  |
| Agree | 50.00% | 43.82% | 33.33% | 40.56% | 42.50% |  |
| Strongly agree | 40.00% | 37.08% | 10.42% | 41.26% | 35.83% |  |
|  |  |  |  |  |  |  |
| Mean | 4.29 | 4.03 | 3.15 | 4.17 | 4.03 | **<.001** |
| Standard deviation | 0.68 | 1.04 | 1.17 | 0.89 | 0.99 |  |
| Range | (2-5) | (1-5) | (1-5) | (1-5) | (1-5) |  |
|  |  |  |  |  |  |  |
| **Treatment 5 - Self-management courses** | | | | | | |
|  |  |  |  |  |  |  |
| **Taking/Having this treatment for back pain makes a lot of sense (1-5)** | |  |  |  |  |  |
| Strongly disagree | 0.00% | 1.12% | 4.17% | 0.70% | 1.11% | **<.001** |
| Disagree | 1.25% | 0.00% | 0.00% | 0.70% | 0.56% |  |
| Neither agree nor disagree | 11.25% | 17.98% | 25.00% | 6.99% | 13.06% |  |
| Agree | 63.75% | 50.56% | 41.67% | 43.36% | 49.44% |  |
| Strongly agree | 23.75% | 30.34% | 29.17% | 48.25% | 35.83% |  |
|  |  |  |  |  |  |  |
| Mean | 4.10 | 4.09 | 3.92 | 4.38 | 4.18 | **0.001** |
| Standard deviation | 0.63 | 0.76 | 0.96 | 0.71 | 0.76 |  |
| Range | (2-5) | (1-5) | (1-5) | (1-5) | (1-5) |  |
| **I think this treatment is pretty useless for people with back pain (1-5)*** | |  |  |  |  |  |
| Strongly disagree | 23.75% | 34.83% | 27.08% | 38.46% | 32.78% | **0.012** |
| Disagree | 37.50% | 26.97% | 18.75% | 30.77% | 29.72% |  |
| Neither agree nor disagree | 21.25% | 17.98% | 27.08% | 10.49% | 16.94% |  |
| Agree | 15.00% | 12.36% | 22.92% | 9.79% | 13.33% |  |
| Strongly agree | 2.50% | 7.87% | 4.17% | 10.49% | 7.22% |  |
|  |  |  |  |  |  |  |
| Mean | 2.35 | 2.31 | 2.58 | 2.23 | 2.33 | 0.414 |
| Standard deviation | 1.08 | 1.28 | 1.23 | 1.34 | 1.26 |  |
| Range | (1-5) | (1-5) | (1-5) | (1-5) | (1-5) |  |
| **I have concerns about taking/having this treatment for my back pain (1-5)*** | |  |  |  |  |  |
| Strongly disagree | 30.00% | 35.96% | 25.00% | 45.45% | 36.94% | **0.001** |
| Disagree | 40.00% | 33.71% | 31.25% | 39.16% | 36.94% |  |
| Neither agree nor disagree | 25.00% | 20.22% | 18.75% | 11.89% | 17.78% |  |
| Agree | 3.75% | 7.87% | 18.75% | 2.80% | 6.39% |  |
| Strongly agree | 1.25% | 2.25% | 6.25% | 0.70% | 1.94% |  |
|  |  |  |  |  |  |  |
| Mean | 2.06 | 2.07 | 2.50 | 1.74 | 1.99 | **<.001** |
| Standard deviation | 0.90 | 1.04 | 1.24 | 0.83 | 0.99 |  |
| Range | (1-5) | (1-5) | (1-5) | (1-5) | (1-5) |  |
| **I am confident this treatment would be a suitable treatment for my back pain (1-5)** | | |  |  |  |  |
| Strongly disagree | 1.25% | 0.00% | 6.25% | 1.40% | 1.67% | **0.017** |
| Disagree | 5.00% | 2.25% | 6.25% | 0.70% | 2.78% |  |
| Neither agree nor disagree | 20.00% | 14.61% | 20.83% | 11.19% | 15.28% |  |
| Agree | 53.75% | 50.56% | 41.67% | 48.95% | 49.44% |  |
| Strongly agree | 20.00% | 32.58% | 25.00% | 37.76% | 30.83% |  |
|  |  |  |  |  |  |  |
| Mean | 3.86 | 4.13 | 3.73 | 4.21 | 4.05 | **0.001** |
| Standard deviation | 0.84 | 0.74 | 1.11 | 0.78 | 0.85 |  |
| Range | (1-5) | (2-5) | (1-5) | (1-5) | (1-5) |  |
|  |  |  |  |  |  |  |
| **Treatment 6 - Psychotherapy** | | | | | | |
|  |  |  |  |  |  |  |
| **Taking/Having this treatment for back pain makes a lot of sense (1-5)** | |  |  |  |  |  |
| Strongly disagree | 2.50% | 1.12% | 4.17% | 2.10% | 2.22% | **0.009** |
| Disagree | 12.50% | 5.62% | 10.42% | 2.80% | 6.67% |  |
| Neither agree nor disagree | 28.75% | 25.84% | 39.58% | 23.78% | 27.50% |  |
| Agree | 45.00% | 38.20% | 27.08% | 43.36% | 40.28% |  |
| Strongly agree | 11.25% | 29.21% | 18.75% | 27.97% | 23.33% |  |
|  |  |  |  |  |  |  |
| Mean | 3.50 | 3.89 | 3.46 | 3.92 | 3.76 | **0.001** |
| Standard deviation | 0.94 | 0.93 | 1.05 | 0.90 | 0.96 |  |
| Range | (1-5) | (1-5) | (1-5) | (1-5) | (1-5) |  |
| **I think this treatment is pretty useless for people with back pain (1-5)*** | |  |  |  |  |  |
| Strongly disagree | 17.50% | 23.60% | 16.67% | 31.47% | 24.44% | 0.309 |
| Disagree | 30.00% | 22.47% | 27.08% | 25.87% | 26.11% |  |
| Neither agree nor disagree | 36.25% | 30.34% | 31.25% | 22.38% | 28.61% |  |
| Agree | 12.50% | 14.61% | 14.58% | 11.19% | 12.78% |  |
| Strongly agree | 3.75% | 8.99% | 10.42% | 9.09% | 8.06% |  |
|  |  |  |  |  |  |  |
| Mean | 2.55 | 2.63 | 2.75 | 2.41 | 2.54 | 0.302 |
| Standard deviation | 1.04 | 1.25 | 1.21 | 1.29 | 1.22 |  |
| Range | (1-5) | (1-5) | (1-5) | (1-5) | (1-5) |  |
| **I have concerns about taking/having this treatment for my back pain (1-5)*** | |  |  |  |  |  |
| Strongly disagree | 20.00% | 31.46% | 25.00% | 37.76% | 30.56% | **0.078** |
| Disagree | 30.00% | 29.21% | 31.25% | 29.37% | 29.72% |  |
| Neither agree nor disagree | 32.50% | 28.09% | 20.83% | 23.08% | 26.11% |  |
| Agree | 15.00% | 6.74% | 10.42% | 6.99% | 9.17% |  |
| Strongly agree | 2.50% | 4.49% | 12.50% | 2.80% | 4.44% |  |
|  |  |  |  |  |  |  |
| Mean | 2.50 | 2.24 | 2.54 | 2.08 | 2.27 | **0.015** |
| Standard deviation | 1.06 | 1.11 | 1.32 | 1.07 | 1.12 |  |
| Range | (1-5) | (1-5) | (1-5) | (1-5) | (1-5) |  |
| **I am confident this treatment would be a suitable treatment for my back pain (1-5)** | | |  |  |  |  |
| Strongly disagree | 8.75% | 2.25% | 14.58% | 1.40% | 5.00% | **0.001** |
| Disagree | 8.75% | 6.74% | 8.33% | 5.59% | 6.94% |  |
| Neither agree nor disagree | 36.25% | 28.09% | 27.08% | 23.78% | 28.06% |  |
| Agree | 33.75% | 29.21% | 35.42% | 42.66% | 36.39% |  |
| Strongly agree | 12.50% | 33.71% | 14.58% | 26.57% | 23.61% |  |
|  |  |  |  |  |  |  |
| Mean | 3.33 | 3.85 | 3.27 | 3.87 | 3.67 | **<.001** |
| Standard deviation | 1.09 | 1.04 | 1.25 | 0.92 | 1.07 |  |
| Range | (1-5) | (1-5) | (1-5) | (1-5) | (1-5) |  |
|  |  |  |  |  |  |  |

Notes: * the lower the score is, more appreciated is the treatment. For each treatment, Cronbach’s alphas were equal to 0.818, 0.737, 0.766, 0.738, 0.652, and 0.712, respectively.

^1^The p-values refer to tests between classes using one-way analysis of variance, Kruskal-Wallis H test, Bartlett’s test for equality of variances, Fisher's exact test, and Chi2 test of independence.

| **Four-item Low Back Pain Treatment Beliefs Questionnaire (details)** | **Class 1** | **Class 2** | **Class 3** | **Class 4** | **Class 5** | **Class 6** | **Total** | **P-value**^1^ |
| --- | --- | --- | --- | --- | --- | --- | --- | --- |
|  |  |  |  |  |  |  |  |  |
| **Observations** | 40 | 47 | 37 | 61 | 139 | 36 | 360 |  |
|  | 11.11% | 13.06% | 10.28% | 16.94% | 38.61% | 10.00% | 100.00% |  |
|  |  |  |  |  |  |  |  |  |
| **Treatment 1 - Corticosteroid injections** | | | | | | | | |
|  |  |  |  |  |  |  |  |  |
| **Taking/Having this treatment for back pain makes a lot of sense (1-5)** | | | |  |  |  |  |  |
| Strongly disagree | 2.50% | 38.30% | 8.11% | 9.84% | 20.86% | 33.33% | 19.17% | **<0.001** |
| Disagree | 5.00% | 21.28% | 8.11% | 11.48% | 20.86% | 8.33% | 15.00% |  |
| Neither agree nor disagree | 25.00% | 31.91% | 13.51% | 32.79% | 25.90% | 13.89% | 25.28% |  |
| Agree | 40.00% | 6.38% | 29.73% | 36.07% | 23.02% | 36.11% | 26.94% |  |
| Strongly agree | 27.50% | 2.13% | 40.54% | 9.84% | 9.35% | 8.33% | 13.61% |  |
|  |  |  |  |  |  |  |  |  |
| Mean | 3.85 | 2.13 | 3.86 | 3.25 | 2.79 | 2.78 | **3.01** | **<0.001** |
| Standard deviation | 0.98 | 1.08 | 1.27 | 1.11 | 1.27 | 1.46 | 1.32 |  |
| Range | (1-5) | (1-5) | (1-5) | (1-5) | (1-5) | (1-5) | (1-5) |  |
| **I think this treatment is pretty useless for people with back pain (1-5)*** | | | |  |  |  |  |  |
| Strongly disagree | 25.00% | 14.89% | 43.24% | 19.67% | 21.58% | 27.78% | 23.61% | **0.017** |
| Disagree | 40.00% | 21.28% | 27.03% | 29.51% | 20.86% | 13.89% | 24.44% |  |
| Neither agree nor disagree | 15.00% | 42.55% | 13.51% | 29.51% | 25.90% | 25.00% | 26.11% |  |
| Agree | 15.00% | 4.26% | 10.81% | 11.48% | 19.42% | 16.67% | 14.44% |  |
| Strongly agree | 5.00% | 17.02% | 5.41% | 9.84% | 12.23% | 16.67% | 11.39% |  |
|  |  |  |  |  |  |  |  |  |
| Mean | 2.35 | 2.87 | 2.08 | 2.62 | 2.80 | 2.81 | **2.66** | **0.022** |
| Standard deviation | 1.17 | 1.24 | 1.23 | 1.21 | 1.31 | 1.45 | 1.29 |  |
| Range | (1-5) | (1-5) | (1-5) | (1-5) | (1-5) | (1-5) | (1-5) |  |
| **I have concerns about taking/having this treatment for my back pain (1-5)*** | | | |  |  |  |  |  |
| Strongly disagree | 22.50% | 0.00% | 29.73% | 13.11% | 11.51% | 33.33% | 15.56% | **<0.001** |
| Disagree | 25.00% | 6.38% | 18.92% | 22.95% | 12.23% | 11.11% | 15.28% |  |
| Neither agree nor disagree | 15.00% | 17.02% | 21.62% | 14.75% | 14.39% | 16.67% | 15.83% |  |
| Agree | 30.00% | 21.28% | 18.92% | 26.23% | 35.25% | 13.89% | 27.50% |  |
| Strongly agree | 7.50% | 55.32% | 10.81% | 22.95% | 26.62% | 25.00% | 25.83% |  |
|  |  |  |  |  |  |  |  |  |
| Mean | 2.75 | 4.26 | 2.62 | 3.23 | 3.53 | 2.86 | **3.33** | **<0.001** |
| Standard deviation | 1.32 | 0.97 | 1.38 | 1.38 | 1.31 | 1.62 | 1.41 |  |
| Range | (1-5) | (1-5) | (1-5) | (1-5) | (1-5) | (1-5) | (1-5) |  |
| **I am confident this treatment would be a suitable treatment for my back pain (1-5)** | | | | |  |  |  |  |
| Strongly disagree | 0.00% | 48.94% | 10.81% | 19.67% | 28.06% | 30.56% | 24.72% | **<0.001** |
| Disagree | 7.50% | 8.51% | 2.70% | 11.48% | 14.39% | 16.67% | 11.39% |  |
| Neither agree nor disagree | 27.50% | 36.17% | 16.22% | 26.23% | 26.62% | 16.67% | 25.83% |  |
| Agree | 40.00% | 6.38% | 37.84% | 27.87% | 17.27% | 22.22% | 22.78% |  |
| Strongly agree | 25.00% | 0.00% | 32.43% | 14.75% | 13.67% | 13.89% | 15.28% |  |
|  |  |  |  |  |  |  |  |  |
| Mean | 3.83 | 2.00 | 3.78 | 3.07 | 2.74 | 2.72 | **2.93** | **<0.001** |
| Standard deviation | 0.90 | 1.06 | 1.25 | 1.34 | 1.39 | 1.47 | 1.39 |  |
| Range | (2-5) | (1-4) | (1-5) | (1-5) | (1-5) | (1-5) | (1-5) |  |
|  |  |  |  |  |  |  |  |  |
| **Treatment 2 - Supervised body-mind physical activities** | | | | | | | | |
|  |  |  |  |  |  |  |  |  |
| **Taking/Having this treatment for back pain makes a lot of sense (1-5)** | | | |  |  |  |  |  |
| Strongly disagree | 0.00% | 2.13% | 10.81% | 1.64% | 2.88% | 2.78% | 3.06% | **0.016** |
| Disagree | 7.50% | 2.13% | 5.41% | 6.56% | 2.88% | 8.33% | 4.72% |  |
| Neither agree nor disagree | 22.50% | 8.51% | 21.62% | 34.43% | 17.99% | 33.33% | 21.94% |  |
| Agree | 40.00% | 53.19% | 40.54% | 37.70% | 37.41% | 38.89% | 40.28% |  |
| Strongly agree | 30.00% | 34.04% | 21.62% | 19.67% | 38.85% | 16.67% | 30.00% |  |
|  |  |  |  |  |  |  |  |  |
| Mean | 3.93 | 4.15 | 3.57 | 3.67 | 4.06 | 3.58 | **3.89** | **0.003** |
| Standard deviation | 0.92 | 0.83 | 1.21 | 0.93 | 0.97 | 0.97 | 0.99 |  |
| Range | (2-5) | (1-5) | (1-5) | (1-5) | (1-5) | (1-5) | (1-5) |  |
| **I think this treatment is pretty useless for people with back pain (1-5)*** | | | |  |  |  |  |  |
| Strongly disagree | 30.00% | 21.28% | 27.03% | 21.31% | 31.65% | 19.44% | 26.67% | 0.715 |
| Disagree | 27.50% | 42.55% | 24.32% | 39.34% | 28.78% | 25.00% | 31.39% |  |
| Neither agree nor disagree | 25.00% | 17.02% | 18.92% | 21.31% | 15.83% | 27.78% | 19.44% |  |
| Agree | 12.50% | 14.89% | 21.62% | 14.75% | 14.39% | 22.22% | 15.83% |  |
| Strongly agree | 5.00% | 4.26% | 8.11% | 3.28% | 9.35% | 5.56% | 6.67% |  |
|  |  |  |  |  |  |  |  |  |
| Mean | 2.35 | 2.38 | 2.59 | 2.39 | 2.41 | 2.69 | **2.44** | 0.760 |
| Standard deviation | 1.19 | 1.11 | 1.32 | 1.08 | 1.32 | 1.19 | 1.23 |  |
| Range | (1-5) | (1-5) | (1-5) | (1-5) | (1-5) | (1-5) | (1-5) |  |
| **I have concerns about taking/having this treatment for my back pain (1-5)*** | | | |  |  |  |  |  |
| Strongly disagree | 32.50% | 27.66% | 24.32% | 26.23% | 35.97% | 25.00% | 30.56% | 0.522 |
| Disagree | 27.50% | 40.43% | 32.43% | 39.34% | 32.37% | 25.00% | 33.33% |  |
| Neither agree nor disagree | 22.50% | 21.28% | 21.62% | 24.59% | 23.74% | 25.00% | 23.33% |  |
| Agree | 12.50% | 8.51% | 10.81% | 6.56% | 6.47% | 16.67% | 8.89% |  |
| Strongly agree | 5.00% | 2.13% | 10.81% | 3.28% | 1.44% | 8.33% | 3.89% |  |
|  |  |  |  |  |  |  |  |  |
| Mean | 2.30 | 2.17 | 2.51 | 2.21 | 2.05 | 2.58 | **2.22** | **0.067** |
| Standard deviation | 1.20 | 1.01 | 1.28 | 1.02 | 1.00 | 1.27 | 1.09 |  |
| Range | (1-5) | (1-5) | (1-5) | (1-5) | (1-5) | (1-5) | (1-5) |  |
| **I am confident this treatment would be a suitable treatment for my back pain (1-5)** | | | | |  |  |  |  |
| Strongly disagree | 0.00% | 4.26% | 8.11% | 8.20% | 4.32% | 2.78% | 4.72% | **0.002** |
| Disagree | 5.00% | 2.13% | 5.41% | 11.48% | 0.72% | 13.89% | 5.00% |  |
| Neither agree nor disagree | 27.50% | 12.77% | 18.92% | 19.67% | 16.55% | 36.11% | 20.00% |  |
| Agree | 40.00% | 44.68% | 48.65% | 42.62% | 46.76% | 38.89% | 44.44% |  |
| Strongly agree | 27.50% | 36.17% | 18.92% | 18.03% | 31.65% | 8.33% | 25.83% |  |
|  |  |  |  |  |  |  |  |  |
| Mean | 3.90 | 4.06 | 3.65 | 3.51 | 4.01 | 3.36 | **3.82** | **<0.001** |
| Standard deviation | 0.87 | 0.99 | 1.11 | 1.16 | 0.95 | 0.93 | 1.03 |  |
| Range | (2-5) | (1-5) | (1-5) | (1-5) | (1-5) | (1-5) | (1-5) |  |
|  |  |  |  |  |  |  |  |  |
| **Treatment 3 - Supervised sports-type physical activities** | | | | | | | | |
|  |  |  |  |  |  |  |  |  |
| **Taking/Having this treatment for back pain makes a lot of sense (1-5)** | | | |  |  |  |  |  |
| Strongly disagree | 0.00% | 4.26% | 13.51% | 6.56% | 4.32% | 13.89% | 6.11% | **0.014** |
| Disagree | 7.50% | 4.26% | 10.81% | 13.11% | 7.19% | 11.11% | 8.61% |  |
| Neither agree nor disagree | 27.50% | 17.02% | 32.43% | 29.51% | 17.99% | 27.78% | 23.33% |  |
| Agree | 42.50% | 51.06% | 32.43% | 34.43% | 36.69% | 38.89% | 38.61% |  |
| Strongly agree | 22.50% | 23.40% | 10.81% | 16.39% | 33.81% | 8.33% | 23.33% |  |
|  |  |  |  |  |  |  |  |  |
| Mean | 3.80 | 3.85 | 3.16 | 3.41 | 3.88 | 3.17 | **3.64** | **<0.001** |
| Standard deviation | 0.88 | 0.98 | 1.19 | 1.12 | 1.09 | 1.18 | 1.11 |  |
| Range | (2-5) | (1-5) | (1-5) | (1-5) | (1-5) | (1-5) | (1-5) |  |
| **I think this treatment is pretty useless for people with back pain (1-5)*** | | | |  |  |  |  |  |
| Strongly disagree | 22.50% | 23.40% | 18.92% | 27.87% | 33.09% | 13.89% | 26.39% | 0.250 |
| Disagree | 37.50% | 44.68% | 24.32% | 24.59% | 27.34% | 22.22% | 29.44% |  |
| Neither agree nor disagree | 20.00% | 14.89% | 29.73% | 27.87% | 17.27% | 36.11% | 22.22% |  |
| Agree | 12.50% | 12.77% | 18.92% | 14.75% | 14.39% | 25.00% | 15.56% |  |
| Strongly agree | 7.50% | 4.26% | 8.11% | 4.92% | 7.91% | 2.78% | 6.39% |  |
|  |  |  |  |  |  |  |  |  |
| Mean | 2.45 | 2.30 | 2.73 | 2.44 | 2.37 | 2.81 | **2.46** | 0.269 |
| Standard deviation | 1.20 | 1.10 | 1.22 | 1.19 | 1.29 | 1.06 | 1.21 |  |
| Range | (1-5) | (1-5) | (1-5) | (1-5) | (1-5) | (1-5) | (1-5) |  |
| **I have concerns about taking/having this treatment for my back pain (1-5)*** | | | |  |  |  |  |  |
| Strongly disagree | 22.50% | 17.02% | 24.32% | 21.31% | 30.22% | 11.11% | 23.61% | **0.008** |
| Disagree | 32.50% | 46.81% | 13.51% | 29.51% | 25.18% | 25.00% | 28.33% |  |
| Neither agree nor disagree | 12.50% | 17.02% | 27.03% | 8.20% | 22.30% | 25.00% | 18.89% |  |
| Agree | 22.50% | 6.38% | 24.32% | 31.15% | 16.55% | 25.00% | 20.00% |  |
| Strongly agree | 10.00% | 12.77% | 10.81% | 9.84% | 5.76% | 13.89% | 9.17% |  |
|  |  |  |  |  |  |  |  |  |
| Mean | 2.65 | 2.51 | 2.84 | 2.79 | 2.42 | 3.06 | **2.63** | **0.082** |
| Standard deviation | 1.33 | 1.23 | 1.34 | 1.36 | 1.24 | 1.24 | 1.29 |  |
| Range | (1-5) | (1-5) | (1-5) | (1-5) | (1-5) | (1-5) | (1-5) |  |
| **I am confident this treatment would be a suitable treatment for my back pain (1-5)** | | | | |  |  |  |  |
| Strongly disagree | 0.00% | 4.26% | 10.81% | 16.39% | 6.47% | 16.67% | 8.61% | **<0.001** |
| Disagree | 10.00% | 10.64% | 16.22% | 18.03% | 9.35% | 8.33% | 11.67% |  |
| Neither agree nor disagree | 17.50% | 12.77% | 32.43% | 19.67% | 13.67% | 36.11% | 19.17% |  |
| Agree | 60.00% | 46.81% | 27.03% | 31.15% | 38.13% | 27.78% | 38.33% |  |
| Strongly agree | 12.50% | 25.53% | 13.51% | 14.75% | 32.37% | 11.11% | 22.22% |  |
|  | 100.00% | 100.00% | 100.00% | 100.00% | 100.00% | 100.00% | 100.00% |  |
| Mean | 3.75 | 3.79 | 3.16 | 3.10 | 3.81 | 3.08 | **3.54** | **<0.001** |
| Standard deviation | 0.81 | 1.08 | 1.19 | 1.33 | 1.18 | 1.23 | 1.20 |  |
| Range | (2-5) | (1-5) | (1-5) | (1-5) | (1-5) | (1-5) | (1-5) |  |
|  |  |  |  |  |  |  |  |  |
| **Treatment 4 - Physical manipulations** | | | | | | | | |
|  |  |  |  |  |  |  |  |  |
| **Taking/Having this treatment for back pain makes a lot of sense (1-5)** | | | |  |  |  |  |  |
| Strongly disagree | 2.50% | 2.13% | 5.41% | 0.00% | 2.16% | 8.33% | 2.78% | **0.013** |
| Disagree | 5.00% | 2.13% | 5.41% | 1.64% | 1.44% | 8.33% | 3.06% |  |
| Neither agree nor disagree | 10.00% | 8.51% | 18.92% | 8.20% | 12.95% | 25.00% | 13.06% |  |
| Agree | 40.00% | 40.43% | 40.54% | 45.90% | 31.65% | 41.67% | 38.06% |  |
| Strongly agree | 42.50% | 46.81% | 29.73% | 44.26% | 51.80% | 16.67% | 43.06% |  |
|  |  |  |  |  |  |  |  |  |
| Mean | 4.15 | 4.28 | 3.84 | 4.33 | 4.29 | 3.50 | **4.16** |  |
| Standard deviation | 0.98 | 0.88 | 1.09 | 0.70 | 0.90 | 1.13 | 0.95 | **<0.001** |
| Range | (1-5) | (1-5) | (1-5) | (2-5) | (1-5) | (1-5) | (1-5) |  |
| **I think this treatment is pretty useless for people with back pain (1-5)*** | | | |  |  |  |  |  |
| Strongly disagree | 40.00% | 44.68% | 21.62% | 40.98% | 42.45% | 19.44% | 37.78% | **0.003** |
| Disagree | 27.50% | 29.79% | 32.43% | 26.23% | 20.14% | 13.89% | 23.89% |  |
| Neither agree nor disagree | 15.00% | 12.77% | 21.62% | 14.75% | 13.67% | 44.44% | 17.78% |  |
| Agree | 15.00% | 4.26% | 18.92% | 16.39% | 10.79% | 16.67% | 12.78% |  |
| Strongly agree | 2.50% | 8.51% | 5.41% | 1.64% | 12.95% | 5.56% | 7.78% |  |
|  |  |  |  |  |  |  |  |  |
| Mean | 2.13 | 2.02 | 2.54 | 2.11 | 2.32 | 2.75 | **2.29** | **0.083** |
| Standard deviation | 1.18 | 1.24 | 1.19 | 1.17 | 1.44 | 1.13 | 1.30 |  |
| Range | (1-5) | (1-5) | (1-5) | (1-5) | (1-5) | (1-5) | (1-5) |  |
| **I have concerns about taking/having this treatment for my back pain (1-5)*** | | | |  |  |  |  |  |
| Strongly disagree | 27.50% | 31.91% | 24.32% | 39.34% | 40.29% | 16.67% | 33.61% | **0.004** |
| Disagree | 37.50% | 48.94% | 32.43% | 34.43% | 25.18% | 27.78% | 32.22% |  |
| Neither agree nor disagree | 15.00% | 12.77% | 16.22% | 19.67% | 21.58% | 27.78% | 19.44% |  |
| Agree | 17.50% | 2.13% | 21.62% | 6.56% | 10.07% | 11.11% | 10.56% |  |
| Strongly agree | 2.50% | 4.26% | 5.41% | 0.00% | 2.88% | 16.67% | 4.17% |  |
|  |  |  |  |  |  |  |  |  |
| Mean | 2.30 | 1.98 | 2.51 | 1.93 | 2.10 | 2.83 | **2.19** | **<0.001** |
| Standard deviation | 1.14 | 0.97 | 1.24 | 0.93 | 1.13 | 1.32 | 1.14 |  |
| Range | (1-5) | (1-5) | (1-5) | (1-4) | (1-5) | (1-5) | (1-5) |  |
| **I am confident this treatment would be a suitable treatment for my back pain (1-5)** | | | | |  |  |  |  |
| Strongly disagree | 2.50% | 2.13% | 5.41% | 0.00% | 2.16% | 8.33% | 2.78% | **0.010** |
| Disagree | 7.50% | 4.26% | 8.11% | 1.64% | 5.04% | 16.67% | 6.11% |  |
| Neither agree nor disagree | 5.00% | 8.51% | 13.51% | 11.48% | 13.67% | 25.00% | 12.78% |  |
| Agree | 50.00% | 46.81% | 51.35% | 47.54% | 35.97% | 36.11% | 42.50% |  |
| Strongly agree | 35.00% | 38.30% | 21.62% | 39.34% | 43.17% | 13.89% | 35.83% |  |
|  |  |  |  |  |  |  |  |  |
| Mean | 4.08 | 4.15 | 3.76 | 4.25 | 4.13 | 3.31 | **4.03** | **<0.001** |
| Standard deviation | 0.97 | 0.91 | 1.06 | 0.72 | 0.98 | 1.17 | 0.99 |  |
| Range | (1-5) | (1-5) | (1-5) | (0-5) | (1-5) | (1-5) | (1-5) |  |
|  |  |  |  |  |  |  |  |  |
| **Treatment 5 - Self-management courses** | | | | | | | | |
|  |  |  |  |  |  |  |  |  |
| **Taking/Having this treatment for back pain makes a lot of sense (1-5)** | | | |  |  |  |  |  |
| Strongly disagree | 0.00% | 0.00% | 0.00% | 0.00% | 1.44% | 5.56% | 1.11% | **0.002** |
| Disagree | 2.50% | 0.00% | 0.00% | 1.64% | 0.00% | 0.00% | 0.56% |  |
| Neither agree nor disagree | 12.50% | 6.38% | 21.62% | 14.75% | 9.35% | 25.00% | 13.06% |  |
| Agree | 60.00% | 57.45% | 43.24% | 62.30% | 41.01% | 44.44% | 49.44% |  |
| Strongly agree | 25.00% | 36.17% | 35.14% | 21.31% | 48.20% | 25.00% | 35.83% |  |
|  |  |  |  |  |  |  |  |  |
| Mean | 4.08 | 4.30 | 4.14 | 4.03 | 4.35 | 3.83 | **4.18** | **0.002** |
| Standard deviation | 0.69 | 0.59 | 0.75 | 0.66 | 0.77 | 1.00 | 0.76 |  |
| Range | (2-5) | (3-5) | (3-5) | (2-5) | (1-5) | (1-5) | (1-5) |  |
| **I think this treatment is pretty useless for people with back pain (1-5)*** | | | |  |  |  |  |  |
| Strongly disagree | 37.50% | 23.40% | 24.32% | 22.95% | 41.73% | 30.56% | 32.78% | **0.008** |
| Disagree | 32.50% | 46.81% | 29.73% | 32.79% | 25.18% | 16.67% | 29.72% |  |
| Neither agree nor disagree | 15.00% | 14.89% | 21.62% | 24.59% | 11.51% | 25.00% | 16.94% |  |
| Agree | 15.00% | 8.51% | 8.11% | 16.39% | 11.51% | 25.00% | 13.33% |  |
| Strongly agree | 0.00% | 6.38% | 16.22% | 3.28% | 10.07% | 2.78% | 7.22% |  |
|  |  |  |  |  |  |  |  |  |
| Mean | 2.08 | 2.28 | 2.62 | 2.44 | 2.23 | 2.53 | **2.33** | 0.309 |
| Standard deviation | 1.07 | 1.12 | 1.38 | 1.12 | 1.36 | 1.25 | 1.26 |  |
| Range | (1-4) | (1-5) | (1-5) | (1-5) | (1-5) | (1-5) | (1-5) |  |
| **I have concerns about taking/having this treatment for my back pain (1-5)*** | | | |  |  |  |  |  |
| Strongly disagree | 30.00% | 29.79% | 29.73% | 34.43% | 46.76% | 27.78% | 36.94% | **0.002** |
| Disagree | 40.00% | 46.81% | 29.73% | 42.62% | 35.25% | 25.00% | 36.94% |  |
| Neither agree nor disagree | 15.00% | 17.02% | 27.03% | 19.67% | 15.11% | 19.44% | 17.78% |  |
| Agree | 15.00% | 4.26% | 8.11% | 1.64% | 2.88% | 19.44% | 6.39% |  |
| Strongly agree | 0.00% | 2.13% | 5.41% | 1.64% | 0.00% | 8.33% | 1.94% |  |
|  |  |  |  |  |  |  |  | **<0.001** |
| Mean | 2.15 | 2.02 | 2.30 | 1.93 | 1.74 | 2.56 | **1.99** |  |
| Standard deviation | 1.03 | 0.92 | 1.15 | 0.87 | 0.82 | 1.32 | 0.99 |  |
| Range | (1-4) | (1-5) | (1-5) | (1-5) | (1-4) | (1-5) | (1-5) |  |
| **I am confident this treatment would be a suitable treatment for my back pain (1-5)** | | | | |  |  |  |  |
| Strongly disagree | 0.00% | 0.00% | 0.00% | 1.64% | 1.44% | 8.33% | 1.67% | **0.090** |
| Disagree | 5.00% | 0.00% | 2.70% | 6.56% | 0.72% | 5.56% | 2.78% |  |
| Neither agree nor disagree | 10.00% | 12.77% | 18.92% | 22.95% | 12.23% | 19.44% | 15.28% |  |
| Agree | 57.50% | 55.32% | 48.65% | 47.54% | 47.48% | 44.44% | 49.44% |  |
| Strongly agree | 27.50% | 31.91% | 29.73% | 21.31% | 38.13% | 22.22% | 30.83% |  |
|  |  |  |  |  |  |  |  | **0.002** |
| Mean | 4.08 | 4.19 | 4.05 | 3.80 | 4.20 | 3.67 | **4.05** |  |
| Standard deviation | 0.76 | 0.65 | 0.78 | 0.91 | 0.79 | 1.15 | 0.85 |  |
| Range | (2-5) | (3-5) | (2-5) | (1-5) | (1-5) | (1-5) | (1-5) |  |
|  |  |  |  |  |  |  |  |  |
| **Treatment 6 - Psychotherapy** | | | | | | | | |
|  |  |  |  |  |  |  |  |  |
| **Taking/Having this treatment for back pain makes a lot of sense (1-5)** | | | |  |  |  |  |  |
| Strongly disagree | 0.00% | 0.00% | 5.41% | 1.64% | 2.16% | 5.56% | 2.22% | **<0.001** |
| Disagree | 12.50% | 8.51% | 10.81% | 8.20% | 1.44% | 11.11% | 6.67% |  |
| Neither agree nor disagree | 25.00% | 36.17% | 13.51% | 32.79% | 23.74% | 38.89% | 27.50% |  |
| Agree | 45.00% | 46.81% | 27.03% | 49.18% | 40.29% | 25.00% | 40.28% |  |
| Strongly agree | 17.50% | 8.51% | 43.24% | 8.20% | 32.37% | 19.44% | 23.33% |  |
|  |  |  |  |  |  |  |  |  |
| Mean | 3.68 | 3.55 | 3.92 | 3.54 | 3.99 | 3.42 | **3.76** | **0.001** |
| Standard deviation | 0.92 | 0.77 | 1.23 | 0.83 | 0.90 | 1.11 | 0.96 |  |
| Range | (2-5) | (2-5) | (1-5) | (1-5) | (1-5) | (1-5) | (1-5) |  |
| **I think this treatment is pretty useless for people with back pain (1-5)*** | | | |  |  |  |  |  |
| Strongly disagree | 20.00% | 17.02% | 27.03% | 14.75% | 33.09% | 19.44% | 24.44% | **0.016** |
| Disagree | 27.50% | 38.30% | 21.62% | 32.79% | 20.14% | 25.00% | 26.11% |  |
| Neither agree nor disagree | 40.00% | 31.91% | 10.81% | 32.79% | 25.90% | 33.33% | 28.61% |  |
| Agree | 10.00% | 8.51% | 16.22% | 14.75% | 12.23% | 16.67% | 12.78% |  |
| Strongly agree | 2.50% | 4.26% | 24.32% | 4.92% | 8.63% | 5.56% | 8.06% |  |
|  |  |  |  |  |  |  |  |  |
| Mean | 2.48 | 2.45 | 2.89 | 2.62 | 2.43 | 2.64 | **2.54** | 0.405 |
| Standard deviation | 1.01 | 1.02 | 1.58 | 1.07 | 1.30 | 1.15 | 1.22 |  |
| Range | (1-5) | (1-5) | (1-5) | (1-5) | (1-5) | (1-5) | (1-5) |  |
| **I have concerns about taking/having this treatment for my back pain (1-5)*** | | | |  |  |  |  |  |
| Strongly disagree | 27.50% | 23.40% | 35.14% | 18.03% | 38.13% | 30.56% | 30.56% | **0.057** |
| Disagree | 22.50% | 29.79% | 29.73% | 36.07% | 30.22% | 25.00% | 29.72% |  |
| Neither agree nor disagree | 32.50% | 34.04% | 13.51% | 29.51% | 24.46% | 22.22% | 26.11% |  |
| Agree | 10.00% | 8.51% | 10.81% | 14.75% | 5.76% | 11.11% | 9.17% |  |
| Strongly agree | 7.50% | 4.26% | 10.81% | 1.64% | 1.44% | 11.11% | 4.44% |  |
|  |  |  |  |  |  |  |  |  |
| Mean | 2.48 | 2.40 | 2.32 | 2.46 | 2.02 | 2.47 | **2.27** | **0.037** |
| Standard deviation | 1.22 | 1.08 | 1.36 | 1.01 | 1.00 | 1.34 | 1.12 |  |
| Range | (1-5) | (1-5) | (1-5) | (1-5) | (1-5) | (1-5) | (1-5) |  |
| **I am confident this treatment would be a suitable treatment for my back pain (1-5)** | | | | |  |  |  |  |
| Strongly disagree | 5.00% | 4.26% | 8.11% | 6.56% | 1.44% | 13.89% | 5.00% | **0.014** |
| Disagree | 7.50% | 12.77% | 5.41% | 9.84% | 3.60% | 8.33% | 6.94% |  |
| Neither agree nor disagree | 30.00% | 29.79% | 18.92% | 37.70% | 25.18% | 27.78% | 28.06% |  |
| Agree | 32.50% | 42.55% | 29.73% | 32.79% | 39.57% | 33.33% | 36.39% |  |
| Strongly agree | 25.00% | 10.64% | 37.84% | 13.11% | 30.22% | 16.67% | 23.61% |  |
|  |  |  |  |  |  |  |  |  |
| Mean | 3.65 | 3.43 | 3.84 | 3.36 | 3.94 | 3.31 | **3.67** | **<0.001** |
| Standard deviation | 1.10 | 0.99 | 1.24 | 1.05 | 0.91 | 1.26 | 1.07 |  |
| Range | (1-5) | (1-5) | (1-5) | (1-5) | (1-5) | (1-5) | (1-5) |  |
|  |  |  |  |  |  |  |  |  |

Notes: * the lower the score is, more appreciated is the treatment. For each treatment, Cronbach’s alphas were equal to 0.818, 0.737, 0.766, 0.738, 0.652, and 0.712, respectively.

^1^The p-values refer to tests between classes using one-way analysis of variance, Kruskal-Wallis H test, Bartlett’s test for equality of variances, Fisher's exact test, and Chi2 test of independence.

**References**

1. Dima A, Lewith GT, Little P, Moss-Morris R, Foster NE, Bishop FL. Identifying patients’ beliefs about treatments for chronic low back pain in primary care: a focus group study. *Br J Gen Pract*. 2013;63(612):e490-498. doi:10.3399/bjgp13X669211

2. Dima A, Lewith GT, Little P, et al. Patients’ treatment beliefs in low back pain: development and validation of a questionnaire in primary care. *Pain*. 2015;156(8):1489-1500. doi:10.1097/j.pain.0000000000000193
